# 5-Methoxyflavone suppresses nasopharyngeal carcinoma proliferation through regulating p53/SREBP/SCD signaling-mediated lipid synthesis and ferroptosis

**DOI:** 10.1186/s12896-026-01163-9

**Published:** 2026-05-18

**Authors:** Yiwen Cui, Yan Zhao, Rong Li, Mingyong Li, Tongyuan Deng, Bo Bao, Yingying Hu

**Affiliations:** 1 Department of Pathophysiology, School of Basic Medical Sciences, Guangdong Medical University, Zhanjiang, Guangdong, 524023 China; 2 Department of Head and Neck Oncology, Affiliated Hospital of Guangdong Medical University, Zhanjiang, Guangdong, 524023 China; 3 The People’s Hospital of Gaozhou, Gaozhou, Guangdong, 525200 China

**Keywords:** Nasopharyngeal carcinoma, 5-Methoxyflavone, Cell proliferation, Lipid metabolism, Ferroptosis

## Abstract

**Background:**

Nasopharyngeal carcinoma (NPC) is a highly prevalent malignant epithelial tumor in southern China. Although early-stage NPC patients have a favorable prognosis with concurrent chemoradiotherapy, the limited efficacy of current clinical regimens remains a crucial obstacle in the treatment of advanced NPC patients. 5-Methoxyflavone, a methylated flavonoid, has been reported to exhibit diverse roles in anti-cancer activity and lipid modulation. However, its potential role in NPC remains unclear. This study aims to investigate the effect of 5-Methoxyflavone on NPC proliferation and its underlying mechanism.

**Methods:**

MTT, colony formation, EdU assays, and flow cytometry were performed with various concentrations of 5-Methoxyflavone (0, 40, 60, 80 and 100 µM) to investigate its effect on NPC cell proliferation. A HONE1 xenograft model was established for in vivo validation. Immunoprecipitation and dual-luciferase reporter assay were employed to confirm 5-Methoxyflavone’s regulatory function on the p53/SREBPs pathway. Lipid synthesis was determined by measuring total cholesterol and triglycerides using ELISA and visualizing lipid droplets via Oil Red O staining. Ferroptosis was evaluated by detecting intracellular ferrous ions, lipid ROS, and glutathione (GSH) levels.

**Results:**

5-Methoxyflavone suppressed NPC cell proliferation in a concentration-dependent manner in vitro. In addition, it profoundly inhibited tumor xenograft growth in mice without causing obvious adverse toxicity. Mechanistically, 5-Methoxyflavone deubiquitinated and stabilized p53 via inhibiting MDM2 expression, leading to suppression of SREBPs and their downstream lipid synthesis-related genes, thus inhibiting lipid synthesis in NPC cells. p53 depletion counteracted 5-Methoxyflavone’s inhibitory effect, while SREBP1 knockdown restored proliferation in p53-silenced cells. Furthermore, 5-Methoxyflavone-stimulated downregulation of SREBP1/SCD signaling triggered ferroptosis in NPC cells by promoting iron accumulation, lipid peroxidation, and GSH depletion. Overexpression of SCD reversed the ferroptosis-inducing effects of 5-Methoxyflavone.

**Conclusions:**

Our findings reveal that 5-Methoxyflavone inhibits NPC proliferation by blocking lipid synthesis and inducing ferroptosis via the p53/SREBPs/SCD signaling pathway, providing evidence for the development of innovative drugs for NPC treatment.

**Supplementary Information:**

The online version contains supplementary material available at https://doi.org/10.1186/s12896-026-01163-9.

## Background

Nasopharyngeal carcinoma (NPC) is a malignant epithelial tumor originating from the nasopharynx mucosal lining, with a particularly high prevalence in Southern China and Southeast Asia [1]. In 2022, there were 120,416 new cases and 73,476 deaths of NPC worldwide, with China recording the highest incidence at 51,010 newly diagnosed cases [2]. Combined radio-chemotherapy is the standard upfront treatment for early or locally advanced NPC patients, and yields a satisfying 5-year survival rate. However, 8%-10% of patients will develop local recurrence, and 10%-20% will experience distant metastases [3, 4]. To date, no standardized treatment is available for recurrent or metastatic NPC. Although cisplatin-based chemotherapy remains the mainstay therapy for recurrent and metastatic NPC, its benefit seems obscure. Zhang and his colleagues reported that the median progression-free survival was only 7 months in NPC patients with locally advanced and metastatic disease treated with gemcitabine plus platinum [5]. In recent years, immune checkpoint inhibitors, such as PD-1 antibodies, have emerged as an alternative treatment for recurrent or metastatic NPC following the failure of prior chemotherapy. However, the objective response rates are modest, ranging from approximately 20% to 34% [6–8]. Moreover, EGFR-tyrosine kinase inhibitors (e.g., gefitinib, cetuximab), Epstein-Barr virus (EBV) vaccines, and adoptive immunotherapy show variable efficacy in clinical trials and require further evaluation [9–11]. Therefore, the quest for innovative drugs with potent anticancer efficiency and low toxicity is imperative in NPC clinical management.

Reprogramming of lipid metabolism, featured by increased de novo lipogenesis, is one of the most important hallmarks of malignant tumors [12]. Elevated lipids not only serve as an energy source but also provide the essential material for membrane building and signal transduction, thereby facilitating the rapid proliferation of cancer cells. Emerging clinical and experimental evidence indicates that NPC cells undergo exacerbated de novo lipogenesis to drive their malignant progression [13, 14]. A lipidomics study highlighted substantial alterations in lipid profiles between NPC patients and healthy controls, as well as between patients with and without lymph node or distant metastasis [15]. Elevated serum cholesterol and free fatty acids were strongly associated with recurrence, metastasis, and reduced survival in NPC patients [16, 17]. Consistent with these findings, NPC cells exhibited a more pronounced accumulation of lipid droplets (LDs), which are crucial intracellular energy reservoirs composed of neutral lipids, compared to normal nasopharyngeal epithelial cells [18]. Yin et al. demonstrated that NPC cells internalize LDs via macropinocytosis to facilitate their proliferation and metastasis [19]. Additionally, LDs accumulation contributed to the radiotherapy resistance in NPC [20]. Sterol regulatory element-binding proteins (SREBPs), including SREBP1 and SREBP2, are central lipogenic transcription factors. They bind to the sterol regulatory element (SRE) in the promoters of lipid synthesis enzymes, such as fatty acid synthase (FASN), 3-hydroxy-3-methylglutaryl-CoA reductase (HMGCR), 3-hydroxy-3-methylglutaryl-CoA synthase1 (HMGCS1), and stearoyl-CoA desaturase (SCD). SREBP1 preferentially controls fatty acid synthesis, while SREBP2 regulates cholesterol synthesis [21]. Growing evidence suggests that oncogenes often promote and rely on SREBP activity to drive NPC cell growth. For instance, EBV-encoded latent membrane protein 1 (LMP1) activated SREBP1/FASN-mediated lipid synthesis and LDs formation via mTOR signaling to promote the proliferation of NPC cells [22]. Leptin enhanced NPC cell proliferation by upregulating SREBP1 and its downstream targets, FASN and SCD [23]. Similarly, LINC02570 promoted NPC cell growth by adsorbing miR-4649-3p to upregulate SREBP1 and FASN [24]. Therefore, targeting the SREBPs pathways may represent a promising anti-cancer strategy in NPC.

Ferroptosis is an iron-dependent cell death driven by membrane lipid peroxidation [25]. Lipid peroxides, generated by the attack of reactive oxygen species (ROS) on polyunsaturated fatty acids (PUFA) in the cellular membrane, are the foundation of oxidative stress-induced ferroptosis [26]. Antioxidant enzymes that mitigate ROS accumulation and detoxify lipid peroxides, such as solute carrier family 7 member 11 (SLC7A11) and glutathione peroxidase 4 (GPX4), can inhibit ferroptosis. It is increasingly recognized that aberrant ferroptosis is associated with the progression of NPC. Yuan L et al. reported that EBV infection reduced NPC cell susceptibility to ferroptosis by upregulating SLC7A11 and GPX4. Elevated GPX4 levels correlated with unfavorable clinical outcomes in NPC patients, and GPX4 ablation suppressed NPC cell proliferation [27]. Natural compounds like allicin, luteolin, and solasodine inhibited NPC cell proliferation via promoting ferroptosis [28–30]. Recent evidence has highlighted the pivotal role of lipids in ferroptosis regulation. For example, SREBP1 promoted SCD-mediated production of monounsaturated fatty acids (MUFAs), which are resistant to peroxidation [31, 32]. Activation of HMGCS1 and HMGCR, two key enzymes in the mevalonate pathway, mediated ferroptosis resistance and promoted NPC cell proliferation and metastasis [33, 34]. Thus, modulating lipid metabolism to induce ferroptosis is a potential strategy for NPC therapy.

Flavonoids are natural products renowned for their anti-cancer, anti-inflammatory, and anti-oxidant effects. Research indicates that methylated flavonoids exhibit superior bioactivity and bioavailability compared to non-methylated flavonoids [35]. Among these, 5-Methoxyflavone is a notable methylated flavonoid. Numerous studies have demonstrated its anti-tumor effects in various tumors. Specifically, 5-Methoxyflavone upregulated BAX, caspase-8, and caspase-3, enhancing apoptosis in leukemia cells [36]. It also induced G2/M cell cycle arrest, apoptosis, and autophagy in colon cancer [37]. Our prior study demonstrated that 5-Methoxyflavone triggered G0/G1 phase arrest in lung adenocarcinoma cells via the PI3K/AKT/GSK3β/β-catenin/Cyclin D1 signaling [38]. Recent studies further revealed that 5-Methoxyflavone alleviated fat deposition in non-alcoholic fatty liver disease and mitigated oxidative damage in liver cells by modulating lipid metabolism [39]. Collectively, 5-Methoxyflavone exhibits anti-cancer and lipid metabolism regulatory effects, positioning it as a promising anti-tumor drug candidate. However, its potential role in NPC remains to be investigated.

Herein, our study elucidated for the first time that 5-Methoxyflavone exhibited a potent anti-proliferative effect on NPC cells both in vivo and in vitro. Mechanistic analyses revealed that it hindered MDM2-mediated ubiquitination degradation of p53, thereby enhancing the suppression of SREBPs and their downstream lipid synthesis-related genes, ultimately blocking lipid synthesis in NPC cells. Furthermore, downregulation of SREBP1/SCD signaling induced ferroptosis in NPC cells. Our findings provide a new experimental basis for developing innovative drugs for NPC treatment.

## Materials and methods

### Cell culture

The normal nasopharyngeal epithelial cell line NP460 and the nasopharyngeal carcinoma cell line 5-8F were obtained from the Cancer Center, Integrated Hospital of Traditional Chinese Medicine of Southern Medical University. The nasopharyngeal carcinoma cell line HONE1 was purchased from BeNa Culture Collection. Cells were cultured in RPMI 1640 medium (Gibco) supplemented with 10% fetal bovine serum (Zeta, Paris, France) at 37 °C in a humidified atmosphere containing 5% CO_2_.

### Reagents and antibodies

5-Methoxyflavone (TCI #42079–78-7, Shanghai, China), cycloheximide (MCE #HY-12320, Shanghai, China), Pifithrin-α (MCE #HY-15484, Shanghai, China). Antibodies used in this study: MDM2 (Proteintech #19058–1-AP, Wuhan, China), p-MDM2 (Abcam #ab170880, Cambridge, UK), p53 (Proteintech #10442–1-AP, Wuhan, China), GAPDH (Proteintech #10494–1-AP, Wuhan, China), β-actin (SAB #52901, Maryland, USA), Ubiquitin (Proteintech #10201–2-AP, Wuhan, China), SREBP1 (Abcam #ab313881, Cambridge, UK), SREBP2 (Abcam #ab30682, Cambridge, UK), FASN (Beyotime #AF5168, Shanghai, China), HMGCR (Beyotime #AF7119, Shanghai, China), HMGCS1 (Abcam #ab317626, Cambridge, UK), SCD(Beyotime #AF7944, Shanghai, China), SLC7A11(Proteintech #26864–1-AP, Wuhan, China), GPX4 (ABclonal #A1933, Wuhan, China).

### Cell viability assay

The proliferative capacity of NPC cells was assessed using an MTT assay (Beyotime Biotechnology, Shanghai, China). Cells were seeded in 96-well plates at 3000 cells per well and incubated overnight. They were then treated with different concentrations of 5-Methoxyflavone for 48 hours. MTT solution (5 mg/mL) was added, and cells were incubated at 37 °C for an additional 4 hours. After discarding the medium, DMSO solution (150 μL/well) was added, and the optical density (OD) was measured at a wavelength of 490 nm using a microplate reader (BioTek, VT, USA).

### Colony formation assay

Cells were inoculated in 6-well plates at a density of 500 cells per well and stimulated with varying concentrations of 5-Methoxyflavone for 7 consecutive days. Colonies were fixed with 4% paraformaldehyde for 15 minutes and then stained with crystal violet for 5 minutes. Images were taken using a smartphone, and the number of colonies was quantified.

## EdU assay

The Cell-Light™ EdU Apollo567 In Vitro Kit (RiboBio, Guangzhou, China) was used to assess the EdU incorporation rate following the manufacturer’s protocol. Cells were seeded in 96-well plates at a density of 5000 cells per well and allowed to adhere overnight. They were then treated with varying concentrations of 5-Methoxyflavone for 48 hours. EdU solution A was added to each well and incubated for 2 hours at 37 °C. Subsequently, the cells were fixed with 4% formaldehyde for 30 minutes and permeabilized with 0.1% Triton X-100 for 20 minutes. Following this, the cells were incubated with EdU reagent for 30 min at room temperature and stained with DAPI for 15 minutes. Images were taken using a fluorescence microscope (Olympus, Tokyo, Japan).

### Cell cycle analysis

The cell cycle was analyzed using a commercial kit (Leagene, Beijing, China). Cells were seeded in 6-well plates and treated with varying concentrations of 5-Methoxyflavone for 48 hours. Then, the cells were collected by trypsinization, washed with PBS, and fixed with 70% ethanol overnight at 4 °C. The following day, the cells were incubated with propidium iodide for 30 min at 37 °C and analyzed immediately by a flow cytometer (BD, Franklin Lakes, NJ, USA). The cell proportion in cycle phases was determined using MODFIT software.

### Immunoprecipitation assay

A Co-IP Kit (Thermofish, USA) was utilized to detect the p53-ubiquitin complex. First, 10 μg of p53 antibody was added to 60 μl of protein A/G agarose beads and incubated for 2 hours at room temperature to form p53 antibody-coupled agarose beads. Whole-cell lysates were acquired using IP lysis buffer, and protein concentrations were determined using a BCA assay (Beyotime, Shanghai, China). The lysates were incubated with the p53 antibody-coupled agarose beads at 4 °C overnight. The following day, the beads were centrifuged and washed with lysis buffer. Finally, proteins were eluted with 40 μl of elution buffer, and western blot analysis was performed using ubiquitin and p53 antibodies.

### Immunofluorescence

Cells were washed with PBS and fixed with 4% paraformaldehyde for 15 minutes. They were then permeabilized with 0.3% Triton X-100 for 10 minutes and blocked with 5% BSA for 30 minutes. Subsequently, the cells were incubated overnight at 4 °C with the primary antibody. The next day, after washing with PBS, the cells were incubated with the secondary antibody for 1 hour at room temperature. Finally, nuclei were counterstained with DAPI for 10 minutes, and the cells were observed and photographed under a microscope.

### Measurement of total cholesterol (TC) and triglyceride (TG) levels

Cells were seeded in 6-well plates and treated with 5-Methoxyflavone (100 μM) for 48 hours. TC and TG levels were determined using a Total Cholesterol Assay Kit (Jiancheng, Nanjing, China) and a Triglyceride Assay Kit (Jiancheng, Nanjing, China), respectively, following the manufacturer’s instructions. The concentrations of TC and TG were calculated by measuring the OD value at 500 nm using a microplate reader (BioTek) and presented as mmol per gram of protein (mmol/g protein).

### Oil red O staining

Cells were seeded in 96-well plates and treated with 5-Methoxyflavone (100 μM) for 48 hours. Intracellular lipid droplets were detected using the Oil Red O Stain Kit (Solarbio, Beijing, China) following the manufacturer’s protocol.

### Dual-luciferase reporter assay

PGL6-based luciferase report plasmid containing SREBP motif-binding sequence (Wt-SREBP) and the renilla luciferase reporter plasmid were purchased from Beyotime Biotechnology. These vectors, along with p53 siRNA or a negative control, were co-transfected into NPC cells using jetPRIME Transfection Reagent (Polyplus, Strasbourg, France). After 48-hour treatment with or without 5-Methoxyflavone, firefly and renilla luciferase activity were measured by a Dual-Lumi Kit (Beyotime Biotechnology, Shanghai, China). The firefly luciferase activity was normalized to renilla luciferase activity.

### Small-interfering RNA (siRNA) and plasmid transfection

p53 siRNA and SREBP1siRNA, along with a negative control, were synthesized by Sangon Biotech Co., Ltd. (Shanghai, China). SCD overexpression plasmid and the empty vector control were also purchased from Sangon Biotech Co., Ltd. NPC cells were cultured in 6-well plates and transfected with siRNA or plasmid using the jetPRIME Transfection Reagent when they reached 60%-70% confluence. Western blot analysis was performed 48 hours post-transfection. The siRNA sequences are detailed in Table 1.

**Table 1:** siRNA sequences used in this study

	Sense	Antisense
si-p53-1	ACUACAACUACAUGUGUAA	UUACACAUGUAGUUGUAGU
si-p53-2	AGAUGUUCCGAGAGCUGAA	UUCAGCUCUCGGAACAUCU
si-SREBP1-1	GAAGACAUGCUUCAGCUUA	UAAGCUGAAGCAUGUCUUC
si-SREBP1-2	CCACUCCAUUGAAGAUGUA	UACAUCUUCAAUGGAGUGG
NC	UUCUCCGAACGUGUCACGU	ACGUGACACGUUCGGAGAA

### Iron assay

The FerroOrange Assay Kit (Dojingo, Tokyo, Japan) was employed to measure the intracellular ferrous ion (Fe^2+^) in NPC cells. Briefly, cells were seeded in 12-well plates and treated with varying concentrations of 5-Methoxyflavone for 48 hours. After washing with HBSS, 1 μM FerroOrange working solution was added, followed by incubation at 37 °C for 30 minutes. Then, the cells were observed using fluorescence microscopy (Olympus, Tokyo, Japan).

### Lipid peroxidation measurement

The BOP 581/591 C11 Probe Kit (Dojingo, Tokyo, Japan) was used to quantify Lipid ROS levels. Cells were stimulated with varying concentrations of 5-Methoxyflavone for 48 hours. Then the cells were incubated with 5 μM C11 probe for 30 minutes at 37 °C. Subsequently, the cells were trypsinized, washed with PBS, and suspended in PBS for flow cytometry analysis. Lipid ROS levels were analyzed using the FITC channel on a flow cytometer (BD).

### Determination of glutathione (GSH) levels

Cells were seeded in 6-well plates and treated with 5-Methoxyflavone for 48 hours. GSH levels were measured using a GSH assay kit (Beyotime, Shanghai, China) according to the manufacturer’s instructions.

### Measurement of malondialdehyde (MDA) levels

The MDA levels were measured using a Lipid Peroxidation MDA Assay Kit (Beyotime Biotechnology, Shanghai, China). Briefly, after 5-Methoxyflavone stimulation for 48 hours, cells were lysed, and protein concentration was measured. Then, the protein supernatant was mixed with the MDA working solution and heated at 100 °C for 15 minutes. Subsequently, 200 μL of the supernatant was transferred to a 96-well plate, and the OD value was measured at 532 nm using a microplate reader (BioTek). The MDA concentration was expressed as μmol per milligram of protein (μmol MDA/mg protein).

### Animal experiment

The animal experiment protocol was approved by the Animal Experimentation Ethics Committee of Guangdong Medical University. BALB/c nude mice (aged 3–4 weeks) were purchased from SPF (Beijing) Biotechnology. Briefly, HONE1 cells (1 × 10^7^) were subcutaneously inoculated into the right forelimb of mice. Then, they were randomly assigned into three groups (*n* = 12): Group 1 (solvent-treated control) received daily solvent gavage; Group 2 (5-Methoxyflavone treatment) was gavaged daily with 100 mg/kg 5-Methoxyflavone; Group 3 (DDP treatment) was intraperitoneally administered 3 mg/kg DDP weekly. The experiment lasted 20 days. Tumor volume and body weight were checked every 2 days, with tumor volume calculated using the formula (length × width^2^/2). At the endpoint, mice were anesthetized by intraperitoneal injection of tribromoethanol at a dose of 200 mg/kg and euthanized by cervical dislocation. Tumor tissues were collected for subsequent immunohistochemistry analysis. Additionally, blood samples were obtained to assess liver and kidney functions using an enzyme-linked immunosorbent assay.

### Immunohistochemistry (IHC) and hematoxylin-eosin (H&E) staining

For IHC assay, tumor tissue slides were deparaffinized and rehydrated. Antigen retrieval was conducted in citrate buffer for 3 minutes at 100 °C. Slides were blocked with 5% BSA and incubated with primary antibodies at 4 °C overnight. The next day, slides were cultured with secondary antibody and visualized using DAB. Immunohistochemical intensities were observed and photographed under a microscope (Olympus) at 200 × magnification. For H&E staining, a hematoxylin-eosin staining kit (Solarbio, Beijing, China) was employed to stain the nucleus and cytoplasm of tumor, liver, and kidney tissues.

### Enzyme-linked immunosorbent assay (ELISA)

The serum levels of alanine transaminase (ALT), aspartate transaminase (AST), and serum creatinine (SCr) in tumor samples were detected using ELISA kits (Mlbio, Shanghai, China) according to the manufacturer’s protocols. Concentrations were measured at 450 nm using a microplate reader (BioTek).

### Western blot

Cells were lysed with RIPA buffer containing protease and phosphatase inhibitors. Total protein concentrations were quantified using a BCA protein assay kit. Equal amounts of lysates were separated by SDS-PAGE and transferred to polyvinylidene difluoride membranes (Millipore, MA, USA). Membranes were blocked with 3% skimmed milk, then incubated with primary antibodies at 4 °C overnight. The next day, membranes were incubated with HRP-conjugated secondary antibody, and protein bands were visualized using a chemiluminescent reagent (Beyotime Biotechnology). Band intensities were quantified using ImageJ software, with GAPDH or β-actin as the internal reference.

### Reverse transcription and real-time quantitative PCR (RT-qPCR)

Total RNA was extracted using TRIzol® Reagent (TaKaRa Biotechnology, Shiga, Japan). cDNA was synthesized using a PrimeScript® RT Master Mix kit (TaKaRa Biotechnology, Shiga, Japan). QPCR analysis was conducted in triplicate using a StepOnePlus instrument system (Thermo Fisher Scientific) with TB Green Premix Ex Taq (TaKaRa Biotechnology, Shiga, Japan). GAPDH served as the internal reference, and gene expression was calculated using the 2^−ΔΔCt^ method. The PCR primers are detailed in Table 2.

**Table 2:** Primers used in this study

	Forward	Reverse
SREBP1	5′-GGAGCCATGGATTGCACTTT-3′	5′-TCAAATAGGCCAGGGAAGTCA-3′
SREBP2	5′-CGAATTGAAAGACCTGGTCATG-3′	5′-TCCTCAGAACGCCAGACTTGT-3′
FASN	5′-CACAGGGACAACCTGGAGTT-3′	5′-ACTCCACAGGTGGGAACAAG-3′
HMGCR	5′-CCAACCTACTACCTCAGCAAGCC-3′	5′-CCAGCCATTACGGTCCCACAC-3′
HMGCS1	5′-GATGTGGGAATTGTTGCCCTT-3′	5′-ATTGTCTCTGTTCCAACTTCCAG-3′
SCD	5′-CGACGTGGCTTTTTCTTCTC-3′	5′-CCTTCTCTTTGACAGCTGGG-3′
GAPDH	5′-ACACCCACTCCTCCACCTTTG-3′	5′-TCCACCACCCTGTTGCTGTAG-3′

### Statistical analyses

All data analyses were performed using GraphPad Prism 10.0 (GraphPad, La Jolla, CA, USA). Data were expressed as mean ± standard deviation (SD) of three independent experiments. Student’s t-test compared two groups, while one-way ANOVA and Dunnett’s tests compared more than two groups. A *p*-value < 0.05 was considered significant, and levels were denoted as follows: * *p* < 0.05; ** *p* < 0.01; and ***p < 0.001.

## Results

### 5-Methoxyflavone inhibited the proliferation of NPC cells in vitro

**Fig. 1:**
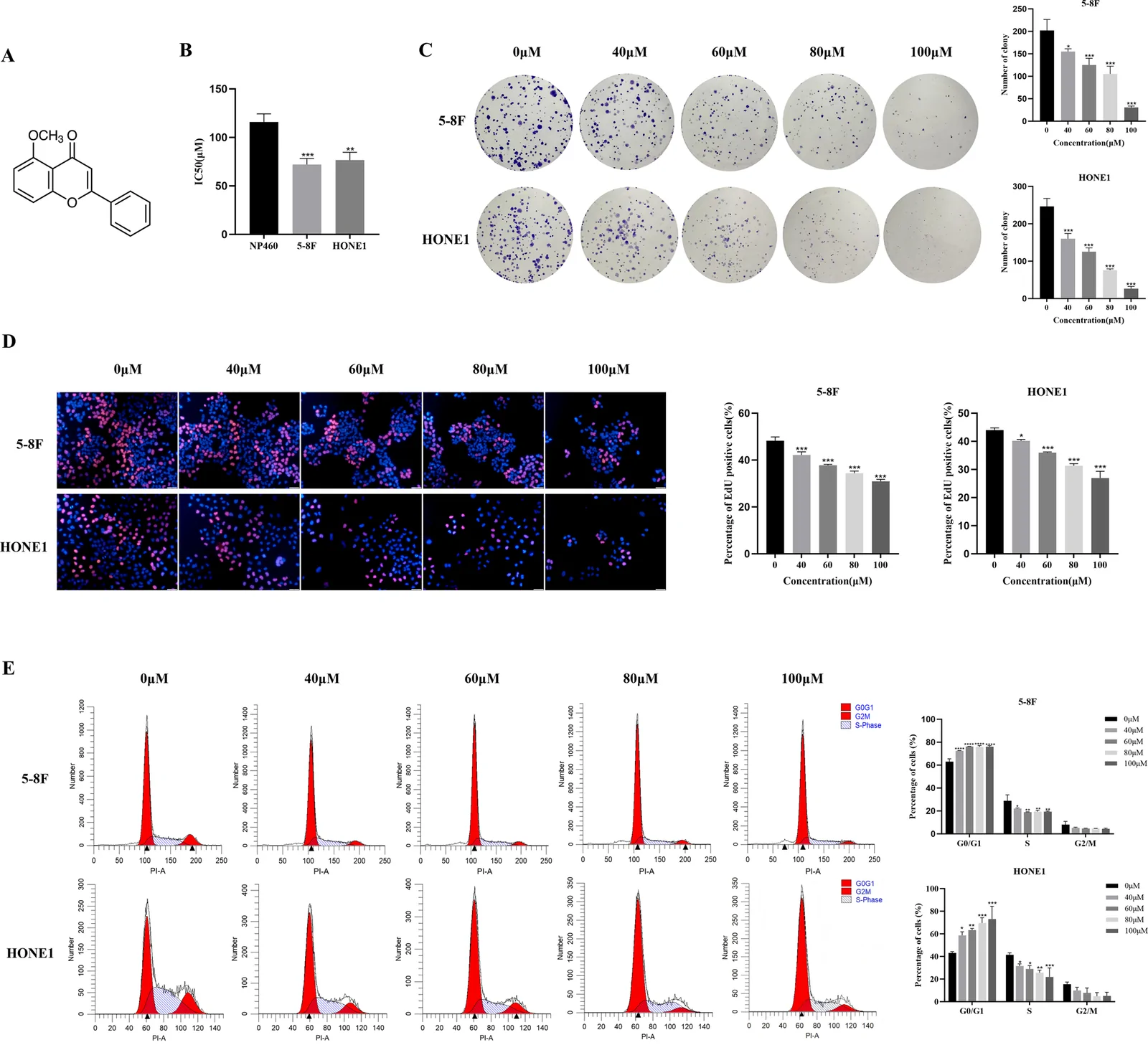
5-Methoxyflavone (5-MF) attenuated NPC cell proliferation in vitro. (**A**) The chemical structure of 5-MF. (**B**) NP460, 5-8F, and HONE1 cells were treated with 5-MF for 48 hours, and IC_50_ was determined by MTT assay. (**C**) Colony formation assay assessed NPC cell clonogenicity after 7 days of 5-MF treatment. Representative images of colonies and corresponding statistical analyses are displayed. (**D**) EdU assay evaluated EdU-positive cells after 48 hours of 5-MF treatment. Representative images and corresponding statistical analyses are displayed (red = EdU-positive cells; blue = DAPI-stained nuclei; scale bar = 50 μm). (**E**) Flow cytometry analyzed cell cycle distributions in NPC cells after 48 hours of 5-MF treatment. Representative images and corresponding statistical analyses are displayed. Data represent mean ± SD of three independent experiments. *p < 0.05, **p < 0.01,***p < 0.001 relative to the control (5-MF 0 μM) group

The chemical structure of 5-Methoxyflavone is illustrated in Fig. 1A. The half-maximal inhibitory concentration (IC_50_) values for 5-Methoxyflavone were 115.9 ± 8.3 μM for NP460 cells, 72.0 ± 6.2 μM for 5-8F cells, and 76.6 ± 7.9 μM for HONE1 cells, respectively (Fig. 1B). These values indicated that NPC cells were more sensitive to 5-Methoxyflavone than normal nasopharyngeal epithelial cells at equivalent concentrations. The colony-formation assay further revealed that 5-Methoxyflavone-treated NPC cells displayed a remarkably dose-dependent reduction in the number of colonies (Fig. 1C).

Additionally, EdU and flow cytometry assays were performed to investigate the impact of 5-Methoxyflavone on the cell cycle of NPC cells. A notable decrease in EdU-positive cells was observed in 5-Methoxyflavone-treated NPC cells compared to the control group (Fig. 1D). Consistent with that, 5-Methoxyflavone treatment led to a significant reduction in the proportion of S-phase cells, accompanied by a corresponding increase in G0/G1-phase cells, further confirming its cell-cycle arrest function (Fig. 1E). Therefore, we concluded that 5-Methoxyflavone inhibited the proliferation of NPC by inducing G0/G1 cell-cycle arrest.

### 5-Methoxyflavone hindered MDM2-mediated ubiquitination degradation of p53

**Fig. 2:**
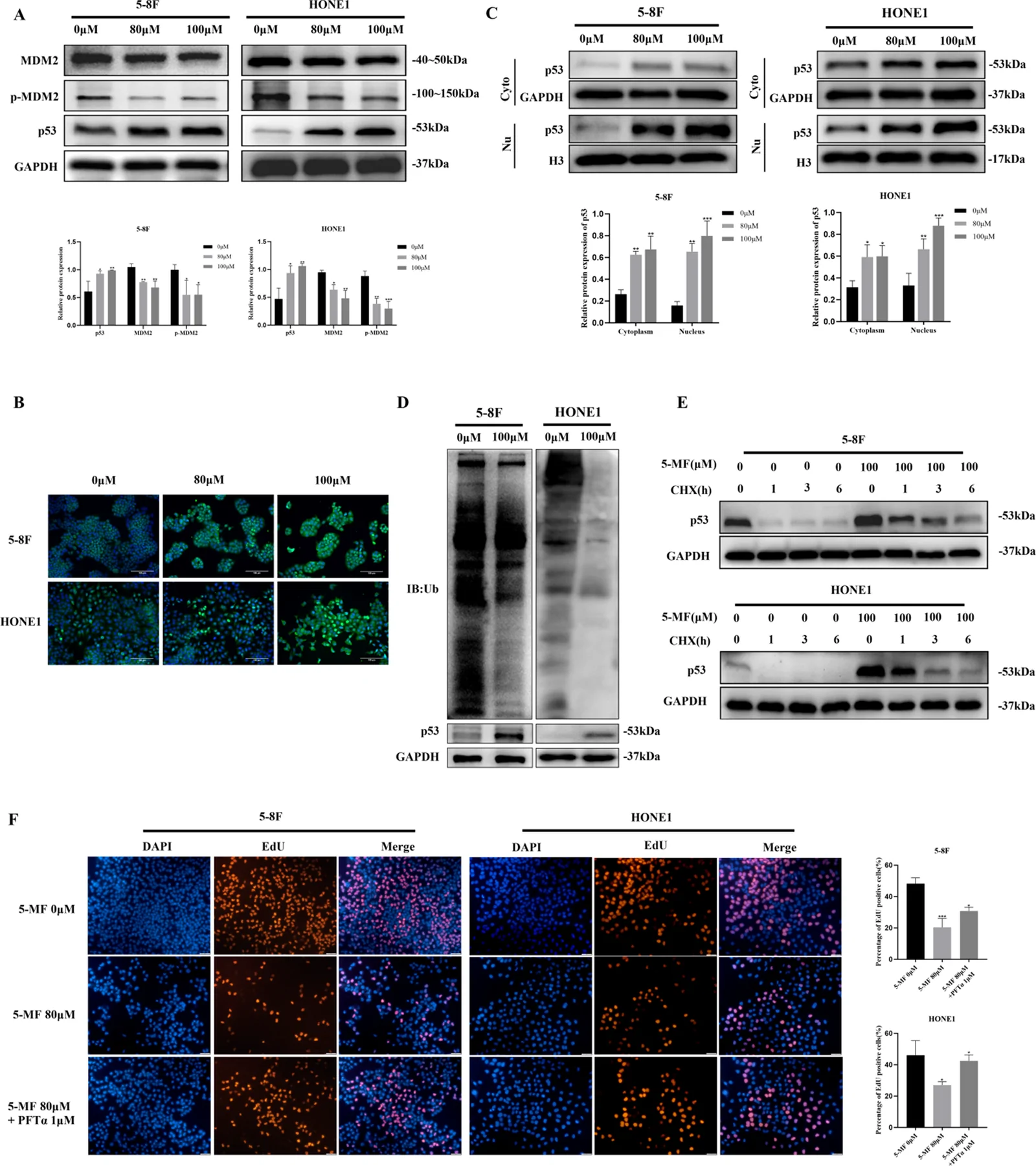
5-Methoxyflavone (5-MF) inhibited MDM2-mediated p53 ubiquitination and degradation. (**A**)Western blot showing the protein levels of MDM2, p-MDM2, and p53 following 48 hours of 5-MF treatment. (**B**) Immunostaining of p53 following 48 hours of 5-MF treatment (scale bar = 150 μm). (**C**) Western blot demonstrating p53 distribution in cytoplasm and nucleus following 48 hours of 5-MF stimulation. (**D**) NPC cells were stimulated with or without 5-MF for 48 hours, followed by immunoprecipitation targeting p53. p53 ubiquitination (ub) level was detected using an anti-ubiquitin antibody (ub). (**E**) NPC cells were treated with cycloheximide (CHX, 20 μM) in the presence or absence of 5-MF for 0, 1, 3, and 6 hours. Western blot showing p53 protein levels at each time point. (**F**) EdU assay illustrating EdU-positive cells in NPC cells treated with or without 5-MF for 48 hours after adding a p53 inhibitor, pifithrin-α (PFTα, 1 μM). (scale bar = 50 μm). Data represent mean ± SD of three independent experiments. *p < 0.05, **p < 0.01,***p < 0.001 relative to the control (5-MF 0 μM) group

p53 is a well-established tumor suppressor protein that prevents aberrant cell growth by inducing cell cycle arrest. Our study demonstrated that 5-Methoxyflavone remarkably upregulated p53 protein expression (Fig. 2A). In accordance with that, Immunofluorescence staining further confirmed a robust increase in p53 fluorescence intensity following 5-Methoxyflavone treatment (Fig. 2B). Notably, nuclear p53 expression was also substantially elevated, suggesting that 5-Methoxyflavone not only upregulated p53 but also facilitated its nuclear translocation (Fig. 2C).

Several milestones have shown that MDM2, the prime negative regulator of p53, mediates proteasomal degradation of p53 by directly ubiquitinating p53 through its E3 ligase activity [40]. Moreover, MDM2 promotes the nuclear export of p53, preventing p53 interactions with its targeted genes and attenuating its transcriptional ability (Wang et al., 2017). As anticipated, 5-Methoxyflavone treatment resulted in a substantial reduction in MDM2 and phosphorylated MDM2 levels (Fig. 2A) and p53 ubiquitination levels (Fig. 2D). Moreover, p53 protein degradation was significantly slower in 5-Methoxyflavone-treated NPC cells compared to controls after blocking protein synthesis by cycloheximide (a protein synthesis inhibitor) (Fig. 2E), indicating that 5-Methoxyflavone enhanced p53 stability and inhibited its proteasomal degradation, which may contribute to the inactivation of MDM2 in NPC cells.

Furthermore, in a rescue assay, the addition of a p53 inhibitor reversed the anti-proliferative effect of 5-Methoxyflavone by increasing the EdU-positive cells, further confirming that p53 is critical for 5-Methoxyflavone-induced cell growth arrest (Fig. 2F). Taken together, our data suggested that 5-Methoxyflavone hindered MDM2-mediated ubiquitination degradation of p53, thereby exerting its anti-proliferative effect in NPC cells.

### 5-Methoxyflavone suppressed lipid synthesis by inhibiting the SREBPs signaling pathway

**Fig. 3:**
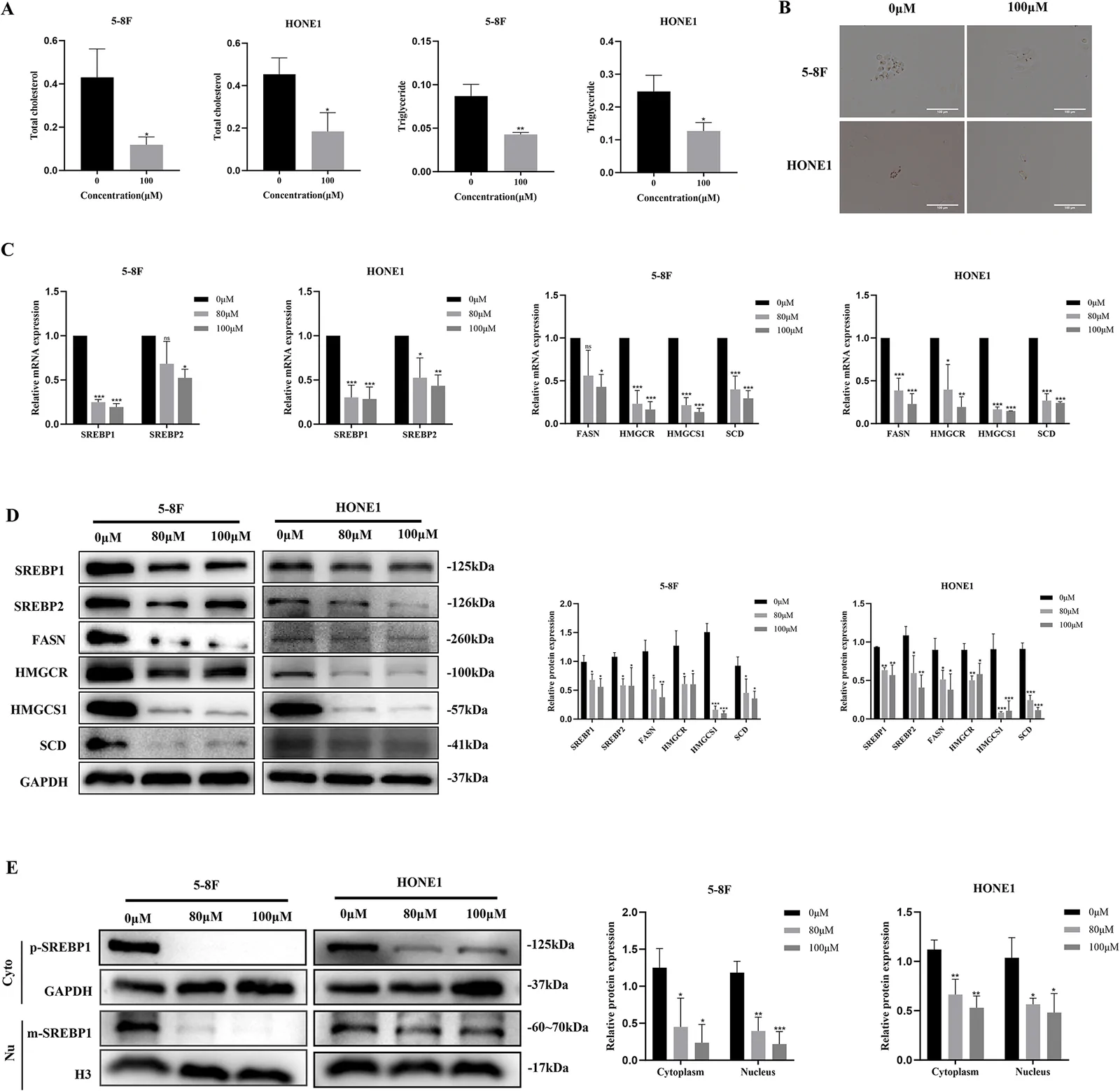
5-Methoxyflavone (5-MF) reduced lipid synthesis through the SREBPs signaling. (**A**) Total cholesterol (TC) and triglyceride (TG) levels in NPC cells after 48-hour 5-MF treatment. (**B**) Oil Red O-stained NPC cells after 48-hour 5-MF treatment (scale bar = 100 μm). (**C**) RT-qPCR analysis of SREBP1, SREBP2, FASN, HMGCR, HMGCS1, and SCD mRNA levels after 48-hour 5-MF treatment. (**D**) Western blot analysis of SREBP1, SREBP2, FASN, HMGCR, HMGCS1, and SCD protein levels after 48-hour 5-MF treatment. (**E**) Western blot analysis of cytoplasmic precursor SREBP1 (p-SREBP1) and nuclear mature SREBP1 (m-SREBP1) levels in NPC cells after 48-hour 5-MF treatment. Data represent mean ± SD of three independent experiments. *p < 0.05, **p < 0.01,***p < 0.001 relative to the control (5-MF 0 μM) group.

Lipids are essential to malignant tumors as increased lipids are required for plasma membrane synthesis and energy production to sustain aberrant, rapid cellular replication [41]. In this study, we found that 5-Methoxyflavone perturbed lipid homeostasis via significantly reducing total cholesterol and triglyceride concentrations in NPC cells (Fig. 3A). Additionally, Oil Red O staining also revealed dramatically fewer LDs in the 5-Methoxyflavone-treated cells compared to the control cells (Fig. 3B).

To elucidate the underlying mechanisms of 5-Methoxyflavone-induced lipid disturbances, RT-qPCR and western blot analysis were performed to detect the expression of the SREBPs pathway, which is critical in regulating lipid synthesis. Results showed that 5-Methoxyflavone significantly attenuated the mRNA and protein expressions of SREBP1/2. In line with this, 5-Methoxyflavone also strongly downregulated SREBPs target genes, including FASN, HMGCR, HMGCS1, and SCD (Figs. 3C, 3D). SREBPs are initially synthesized as inactive precursors, cleaved into their active mature form in the Golgi, and translocate to the nucleus to initiate the transcription of target genes. Accompanied by an obvious reduction in SREBP1 precursor level in cytoplasm, 5-Methoxyflavone-treated cells also displayed a marked decrease in the level of mature SREBP1 in the nucleus compared with the control cells (Fig. 3E). Altogether, these findings demonstrated a potent suppression function of 5-Methoxyflavone on lipid synthesis via inhibiting SREBP1/2 signaling pathway in NPC cells.

### 5-Methoxyflavone inhibited NPC cell proliferation through the p53/SREBPs signaling pathway

**Fig. 4:**
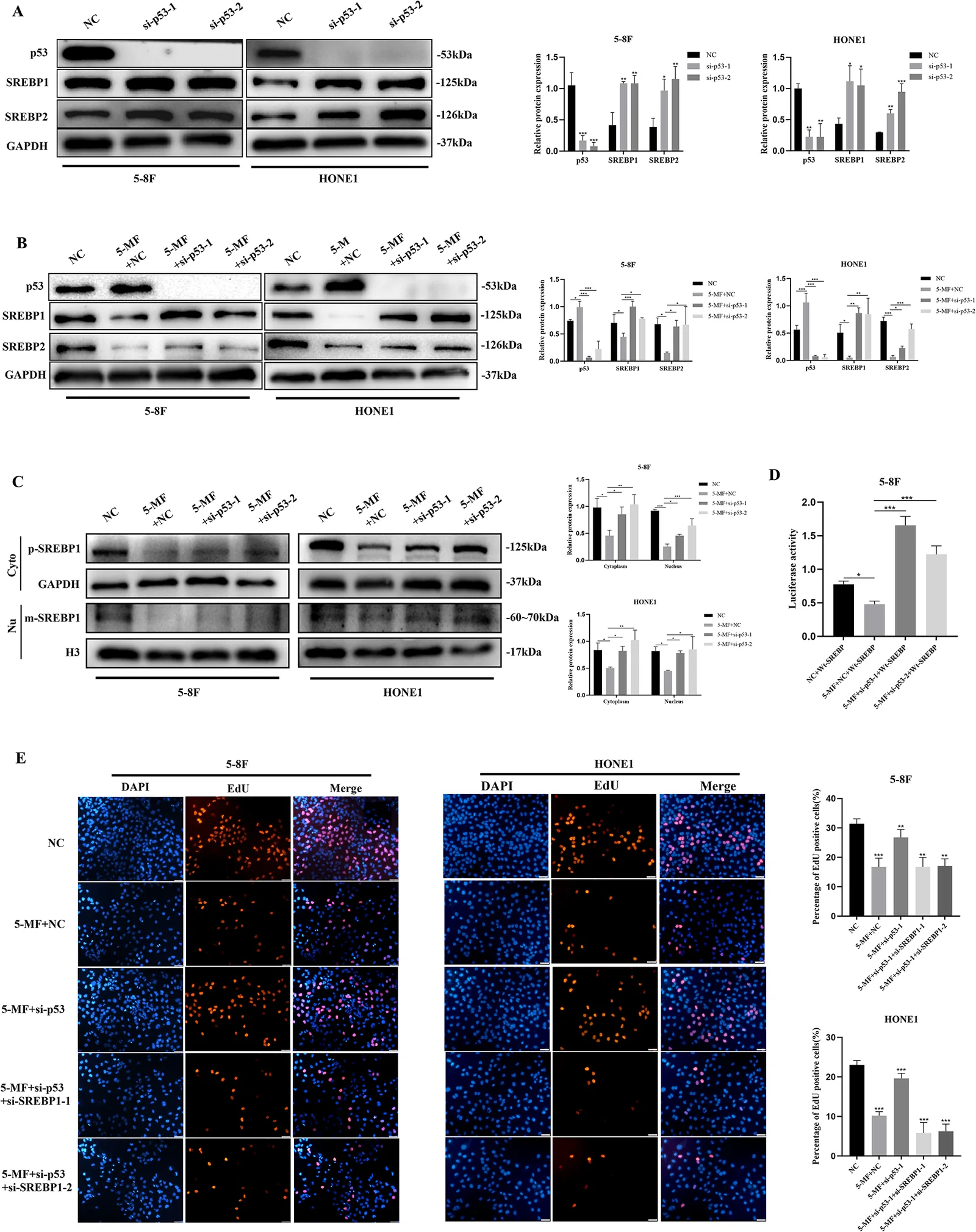
5-Methoxyflavone (5-MF) suppressed SREBPs signaling via upregulating p53. (**A**) Western blot assessed SREBP1 and SREBP2 protein levels after p53 knockdown. (**B**) Western blot evaluated SREBP1 and SREBP2 protein levels in 5-MF-treated (100 μM) cells after p53 knockdown. (**C**) Western blot measured p-SREBP1 and m-SREBP1 levels in 5-MF-treated (100 μM) cells after p53 silencing. (**D**) Dual-luciferase reporter assay determined wt-SREBP luciferase activities in 5-MF-treated (100 μM) cells after p53 silencing. (**E**) EdU assay detected EdU-positive cells after cotransfection with p53 siRNA and SREBP1 siRNA in 5-MF-treated (100 μM) cells. Representative images and corresponding statistical analyses are displayed (scale bar = 50 μm). Data represent mean ± SD of three independent experiments. *p < 0.05, **p < 0.01, ***p < 0.001 relative to the control (5-MF 0 μM) group except Fig. 4B, C and D

Studies have demonstrated that p53 inhibits cancer cell metabolic reprogramming by suppressing SREBPs transcriptional activity [42]. However, the p53-SREBPs regulatory relationship in NPC cells was unknown. Our findings revealed that p53 knockdown significantly increased SREBP1/2 protein levels, confirming p53's negative regulatory effect on SREBPs in NPC (Fig. 4A). Additionally, 5-Methoxyflavone-induced SREBP1/2 reduction was reversed after p53 knockdown (Fig. 4B). Meanwhile, p53 silencing also elevated mature SREBP1 levels in 5-Methoxyflavone-treated NPC cells (Fig. 4C). A dual-luciferase reporter assay revealed a significant decrease in WT-SREBPs reporter luciferase activity after 5-Methoxyflavone treatment, which was strikingly rescued by co-transfecting p53 siRNA (Fig. 4D). These results suggested that activation of p53 by 5-Methoxyflavone might explain its role in SREBPs suppression.

Next, we proceeded to confirm that p53/SREBPs signaling mediated the anti-proliferative function of 5-Methoxyflavone on NPC cells. As illustrated in Fig. 4E, p53 knockdown counteracted 5-Methoxyflavone’s inhibitory effect on NPC cell proliferation by increasing EdU-positive cells. Conversely, SREBP1 downregulation restored proliferation in p53-slicing cells (Fig. 4E). These rescue experiments indicated that 5-Methoxyflavone’s anti-proliferative effect on NPC cells depended on p53-mediated SREBPs downregulation.

### 5-Methoxyflavone triggered ferroptosis in NPC cells

**Fig. 5:**
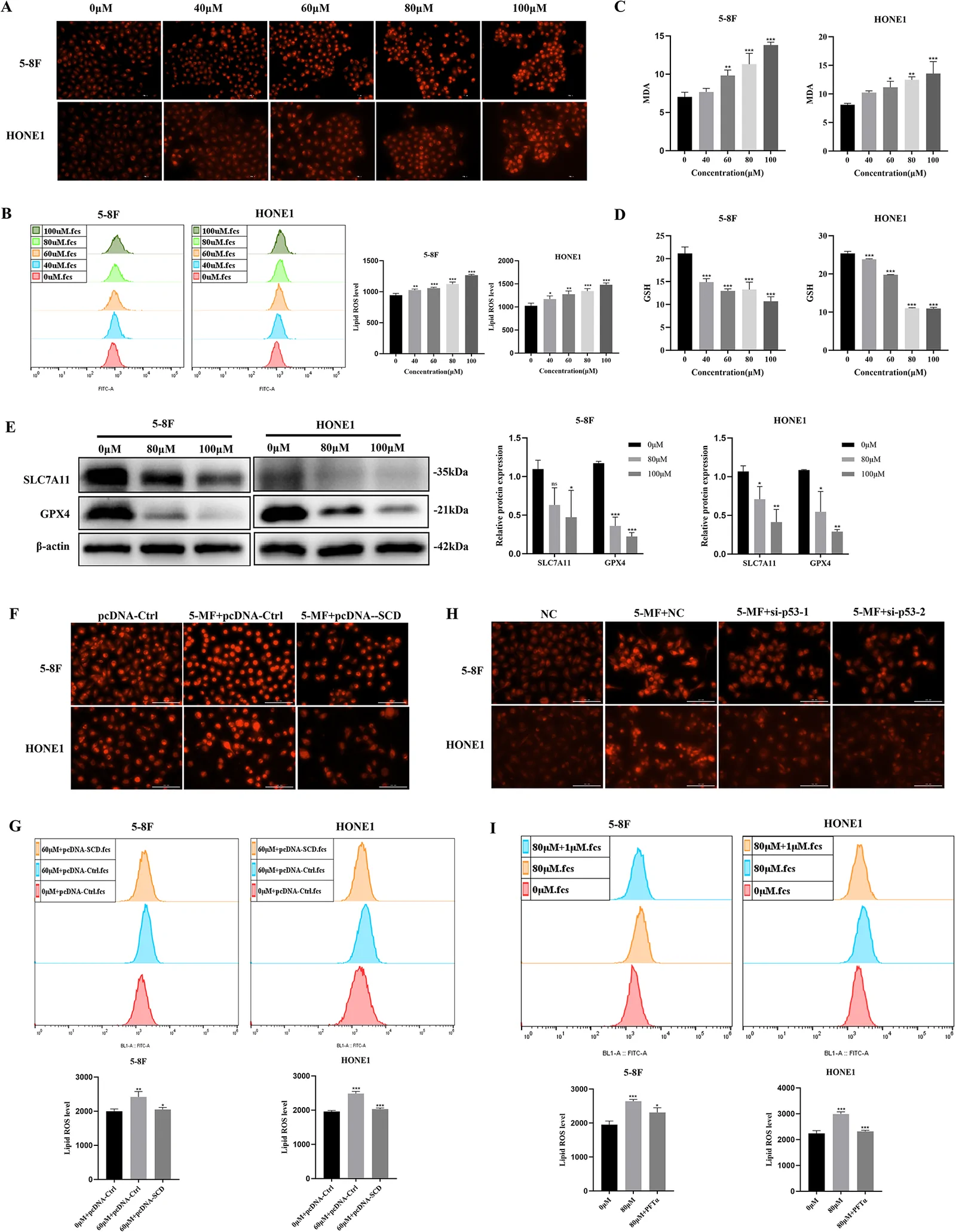
5-Methoxyflavone (5-MF) triggered ferroptosis in NPC cells. (**A**) Intracellular ferrous ion levels in NPC cells after 48 hours of 5-MF treatment (scale bar = 100 μm). (**B**) Lipid ROS levels in NPC cells after 48 hours of 5-MF treatment. (**C**) MDA levels in NPC cells after 48 hours of 5-MF treatment. (**D**) GSH levels in NPC cells after 48 hours of 5-MF treatment. (**E**) GPX4 and SLC7A11 protein expression in NPC cells following 48 hours of 5-MF treatment. (**F**) Intracellular ferrous ion levels in 5-MF-treated NPC cells after SCD overexpression (scale bar = 100 μm). (**G**) Lipid ROS levels in 5-MF-treated NPC cells after SCD overexpression. (**H**) Intracellular ferrous ion levels in 5-MF-treated NPC cells following p53 knockdown (scale bar = 100 μm). (**I**) Lipid ROS levels in 5-MF-treated NPC cells after p53 inhibition by pifithrin-α (PFTα, 1 μM). Data represent mean±SD of three independent experiments. *p < 0.05, **p < 0.01,***p < 0.001 relative to the control (5-MF 0 μM) group

Extensive evidence has revealed that lipid metabolism regulates ferroptosis during cancer initiation and development. Given that 5-Methoxyflavone influenced lipid synthesis, we further explored whether ferroptosis is an additional underlying mechanism of 5-Methoxyflavone-mediated cell proliferation inhibition in NPC cells. Iron accumulation, lipid peroxidation, and GSH depletion are critical processes during ferroptosis [43]. Our findings demonstrated that 5-Methoxyflavone dose-dependently increased intracellular ferrous ion levels in NPC cells (Fig. 5A). It also elevated lipid ROS (Fig. 5B) and MDA levels (Fig. 5C), which are indicators of lipid peroxidation. Conversely, GSH, a crucial intracellular non-enzymatic antioxidant that facilitates the removal of lipid hydroperoxides, was profoundly reduced by 5-Methoxyflavone (Fig. 5D). Additionally, 5-Methoxyflavone significantly lowered SLC7A11 and GPX4 expression, two critical suppressors of ferroptosis (Fig. 5E). Taken together, our data strongly indicated that 5-Methoxyflavone triggered ferroptosis in NPC cells.

SCD protects cancer cells from ferroptosis by producing MUFAs, which inhibit lipid peroxidation at the plasma membrane. Since our studies have confirmed the suppressive role of 5-Methoxyflavone on the SREBP1/SCD signaling pathway, we investigated whether 5-Methoxyflavone-induced ferroptosis was due to SCD suppression. Overexpression of SCD in NPC cells abolished 5-Methoxyflavone-induced ferrous ion accumulation and lipid peroxidation (Fig. 5F). Furthermore, p53 knockdown also reduced intracellular iron accumulation and lipid ROS in 5-Methoxyflavone-treated NPC cells (Fig. 5G). In summary, SCD induction or p53 silencing counteracted 5-Methoxyflavone-induced ferroptosis, indicating 5-Methoxyflavone promoted ferroptosis via the p53/SREBP1/SCD signaling pathway

### 5-Methoxyflavone inhibited NPC cell growth in vivo

**Fig. 6:**
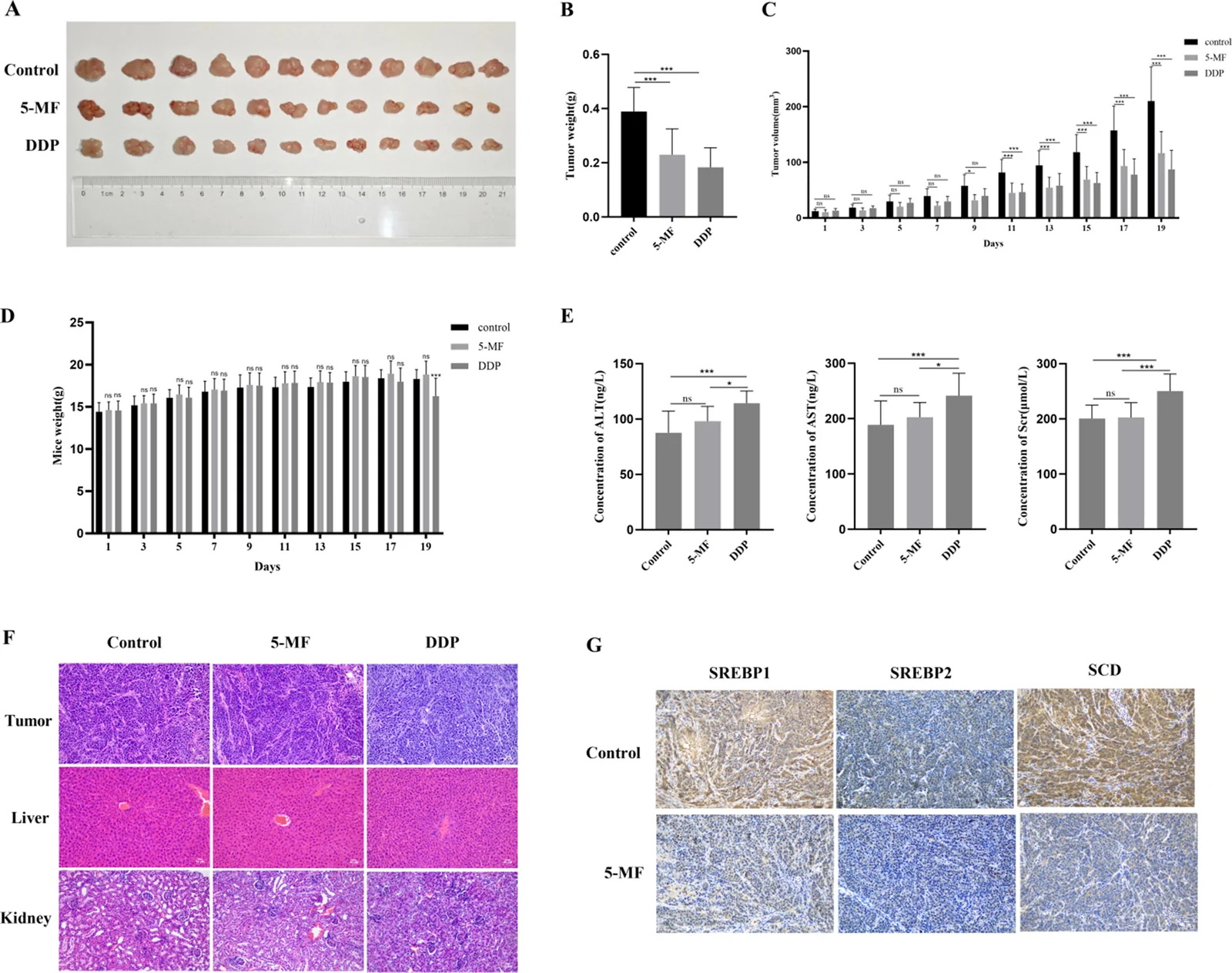
5-Methoxyflavone (5-MF) inhibited NPC cell proliferation in vivo. (**A**) Images of excised xenograft tumors in solvent control, 5-Methoxyflavone, and DDP groups after euthanasia at the endpoint (*n* = 12 per group). (**B**) Weight of excised xenograft tumors in the three groups. (**C**) Tumor volume of subcutaneous tumors in the three groups during the experiment. (**D**) Body weight of mice in the three groups throughout the experiment. (**E**) ELISA assays detected serum levels of AST, ALT, and scr in the three groups at the endpoint. (**F**) H&E staining images of tumor, liver, and kidney tissues (scale bar = 50 μm). (**G**) IHC images showing SREBP1, SREBP2, and SCD expression (scale bar = 50 μm). Data represent mean ± SD of three independent experiments. *p < 0.05, **p < 0.01,***p < 0.001 relative to the solvent control group except Fig. 6E

To further evaluate 5-Methoxyflavone’s role in NPC cell growth, HONE1 cells were injected into BALB/c nude mice and treated with solvent control, 100 mg/kg 5-Methoxyflavone, or 3 mg/kg DDP. The 5-Methoxyflavone-treated group showed a significant reduction in tumor volume (116.55±38.65 mm^3^ vs. 210.27 ± 69.99 mm^3^) and tumor weight (0.22±0.09 g vs. 0.38 ± 0.08 g) compared to the control group at the endpoint. However, neither tumor volume (116.55±38.65 mm^3^ vs. 87.37 ± 34.46 mm^3^) nor tumor weight (0.22±0.09 g vs. 0.18 ± 0.07 g) exhibited a statistically significant difference between the 5-Methoxyflavone-treated and DDP groups (Fig. 6A–C). Notably, no body weight loss and change in serum levels of AST, ALT, and SCr were noted in mice treated with or without 5-Methoxyflavone (Figs. 6D, 6E). This aligns with the H&E staining results, which confirmed no histological damage to kidney and liver tissues upon 5-Methoxyflavone treatment (Fig. 6F). Immunohistochemical analysis showed 5-Methoxyflavone attenuated SREBP1, SRBBP2, and SCD expression in tumors, consistent with in vitro findings (Fig. 6G). The observations indicated that 5-Methoxyflavone exerted an inhibitory effect on NPC cells’ proliferation in vivo through lipid synthesis suppression and ferroptosis induction without organ toxicity.

## Discussion

P53 is a widely recognized tumor suppressor that regulates cell proliferation, DNA repair, senescence, and cell death in cancers. Although p53 is the most frequently mutated gene, with missense mutations occurring in more than 50% of malignant tumors, its mutation in NPC is rare [44, 45]. Increasing evidence suggests that NPC cells inactivate p53 via various mechanisms. For instance, LMP1 induced radioresistance in NPC cells by inhibiting p53 phosphorylation [46]. Additionally, nuclear phosphoprotein 1 (NPM1) promoted NPC metastasis and stemness by recruiting the E3 ubiquitin ligase MDM2 to mediate ubiquitination-dependent proteasomal degradation of p53 [47]. Therefore, reactivating the p53 signaling pathway represents a promising alternative strategy for treating NPC. Ma et al. demonstrated that combining recombinant human adenovirus p53 with chemoradiotherapy significantly enhanced progression-free survival and the 3-year survival rate in recurrent patients with NPC, while also reducing adverse events such as leukopenia and oral mucositis [48]. More recently, several small-molecule drugs that upregulate p53 signaling in NPC are being evaluated [48–52]. Our study revealed for the first time that 5-Methoxyflavone effectively inhibited NPC cell growth in vivo and in vitro without notable renal or hepatotoxicity, underscoring its high efficacy and low toxicity in NPC therapy. Mechanistically, 5-Methoxyflavone suppressed the MDM2-mediated ubiquitinated degradation of p53 and upregulated p53 expression. Blocking p53 signaling reversed 5-Methoxyflavone’s suppressive impact on NPC cell proliferation, highlighting the critical role of p53 in 5-Methoxyflavone’s antitumor mechanism.

Abnormal lipid accumulation plays a crucial role in the malignant progression of NPC and is closely associated with the prognosis of NPC patients. Undoubtedly, SREBPs serve as a central hub for lipid synthesis. Mounting research indicates that NPC cells modulate SREBPs and their downstream lipogenic enzymes, leading to a significant increase in de novo lipid synthesis and rapid cell proliferation. Representatively, LMP activated the expression and maturation of SREBP1 and its downstream target FASN, promoting the NPC cell proliferation [22]. EBV-encoded RNAs (EBERs) upregulated the expression of low-density lipoprotein receptor (LDLR) and FASN, accelerating NPC cell proliferation [53]. Additionally, adipocytes under a hypoxic microenvironment overexpressed hypoxiainducible factor1α (HIF-1α), which subsequently increased lipid accumulation and promoted NPC proliferation via SCD [54]. Therefore, blocking SREBPs-mediated lipid synthesis emerges as a potential therapeutic strategy for NPC. Simvastatin, a lipid-lowering drug, concentration-dependently inhibited NPC cell proliferation by targeting mevalonate, a precursor of cholesterol [55]. Quercetin, a dietary polyphenolic flavonoid, decreased FASN expression, inducing cell cycle arrest in NPC cells [56]. Our research provided convincing evidence that 5-Methoxyflavone downregulated SREBPs and their downstream lipogenic enzymes, thereby reducing triglycerides, cholesterol, and LD levels, which contributed to its proliferation-suppressive effect in NPC cells.

As a pivotal tumor suppressor, p53 has been reported to hinder lipid biosynthesis through influencing SREBPs signaling. Specifically, p53 blocked SREBP2 maturation by transcriptionally inducing ABCA1, a cholesterol transporter gene, to inhibit the mevalonate pathway [57]. Downregulation of large tumor suppressor kinase 2 (LATS2) led to SREBP activation and excessive cholesterol accumulation, while p53 induction compensates for LATS2 loss to restrict SREBP2 activity [58]. Additionally, p53 protected against fatty liver by binding to SREBP1’s promoter, suppressing its transcriptional activity and downstream lipogenesis-related genes [59]. Thus, modulating the p53/SREBPs axis is a promising therapeutic target for cancer treatment [60–62]. In alignment with these studies, our results also validated the inhibitory effect of p53 on SREBPs in NPC cells. p53 knockdown reversed 5-Methoxyflavone’s inhibitory effects on SREBPs. Furthermore, SREBP1 inhibition eliminated cell proliferation caused by p53 compromise in 5-Methoxyflavone-treated cells. These findings suggested that the p53/SREBPs axis represented the molecular mechanism through which 5-Methoxyflavone inhibited lipid synthesis and consequently blocked NPC cell proliferation.

Recent studies have suggested that inhibiting ferroptosis contributes to proliferation, metastasis, radiotherapy resistance, and the formation of an immunosuppressive tumor microenvironment in NPC cells [63–65]. Consequently, drugs inducing ferroptosis are considered potential therapeutic agents in NPC treatment. Lipid metabolism has been reported to overcome ferroptosis via regulating lipid peroxidation [66]. For example, SREBP2 reduced intracellular iron deposition and lipid peroxidation by activating the transcription of the iron carrier Transferrin, thereby conferring resistance to ferroptosis inducers [67]. SREBP1 transcriptionally upregulated GPX4 to reduce phospholipid and cholesterol hydroperoxides [68]. Coenzyme Q10, a metabolite of the SREBP2-mediated mevalonate pathway, acted as an antioxidant to halt lipid peroxidation [69]. Additionally, phospholipid composition influences ferroptosis sensitivity. PUFAs are susceptible to peroxidation, as ROS can react with their tails on the cell membrane lipid bilayer to generate lipid peroxides. In contrast, MUFAs are less prone to oxidation. SCD converts saturated fatty acids into MUFAs, which replace PUFAs in the membrane phospholipids, thereby limiting lipid peroxidation and effectively rescuing cells from ferroptosis [70, 71]. Therefore, modulating lipid metabolism to induce ferroptosis in NPC cells represents an alternative therapeutic strategy. Intriguingly, our findings revealed that 5-Methoxyflavone increased intracellular iron and lipid peroxidation while reducing GSH levels, suggesting its potential to induce ferroptosis in NPC cells. Mechanistically, 5-Methoxyflavone downregulated SREBP1/SCD expression, and SCD overexpression counteracted 5-Methoxyflavone’s effects on iron accumulation and lipid peroxidation. Accordingly, 5-Methoxyflavone promoted ferroptosis through SREBP1/SCD signaling, representing another molecular mechanism underlying its inhibitory function on NPC cell proliferation.

Limitations still exist in this study. Although our study provided compelling evidence that 5-Methoxyflavone upregulated p53 signaling pathways to impede the proliferation of NPC cells, p53 interference only partially reversed 5-Methoxyflavone’s inhibitory effect on proliferation in the rescue experiment, suggesting other molecular mechanisms may be involved. Additionally, 5-Methoxyflavone also suppressed the expression of SLC7A11 and GPX4, two key ferroptosis suppressors. Since p53 can transcriptionally inhibit SLC7A11 expression and induce GPX4 degradation [72, 73], we inferred that 5-Methoxyflavone’s induction of ferroptosis may not be solely dependent on the SREBP1/SCD pathway but also function through the p53/SLC7A11/GPX4 signaling pathway.

## Conclusion

In summary, our study revealed that 5-Methoxyflavone significantly inhibited NPC cell proliferation by suppressing lipid synthesis and triggering ferroptosis via the p53/SREBPs/SCD signaling. More importantly, 5-Methoxyflavone exerted a potent proliferation-inhibiting effect without causing significant adverse consequences in mice. Our findings collectively highlight the promising potential of 5-Methoxyflavone as a novel therapeutic candidate for NPC treatment.

## Supplementary Information

Below is the link to the electronic supplementary material.

Supplementary Material 1

## Data Availability

The datasets used and/or analysed during the current study are available from the corresponding author on reasonable request.

## Abbreviation

LDs: Lipid droplets
EBERs: EBV-encoded RNAs
EBV: Epstein-Barr virus
FASN: Fatty acid synthase
GPX4: Glutathione peroxidase 4
GSH: Glutathione
HIF-1α: Hypoxiainducible factor1α
HMGCR: 3-hydroxy-3-methylglutaryl-CoA reductase
HMGCS1: Hydroxymethylglutaryl-CoA synthase 1
LATS2: Large tumor suppressor kinase 2
LMP1: EBV-encoded latent membrane protein 1
MUFAs: Monounsaturated fatty acids
NPC: Nasopharyngeal carcinoma
NPM1: Nuclear phosphoprotein 1
PUFAs: Polyunsaturated fatty acids
ROS: Reactive oxygen species
SCD: Stearoyl-CoA desaturase 1
SLC7A11: Solute carrier family 7 member 11
SREBPs: Sterol regulatory element-binding proteins.
