# Supplementary material for: 5-Methoxyflavone suppresses nasopharyngeal carcinoma proliferation through regulating p53/SREBP/SCD signaling-mediated lipid synthesis and ferroptosis
Source: BMC Biotechnol. 2026 May 18;26:130. doi: 10.1186/s12896-026-01163-9 (PMC13746537; doi:10.1186/s12896-026-01163-9)
Supplement: Supplementary file 1 [file 12896_2026_1163_MOESM1_ESM.pdf]

Figure 2A (from left to right: 0μM, 80μM,100μM)

**5-8F**

MDM2

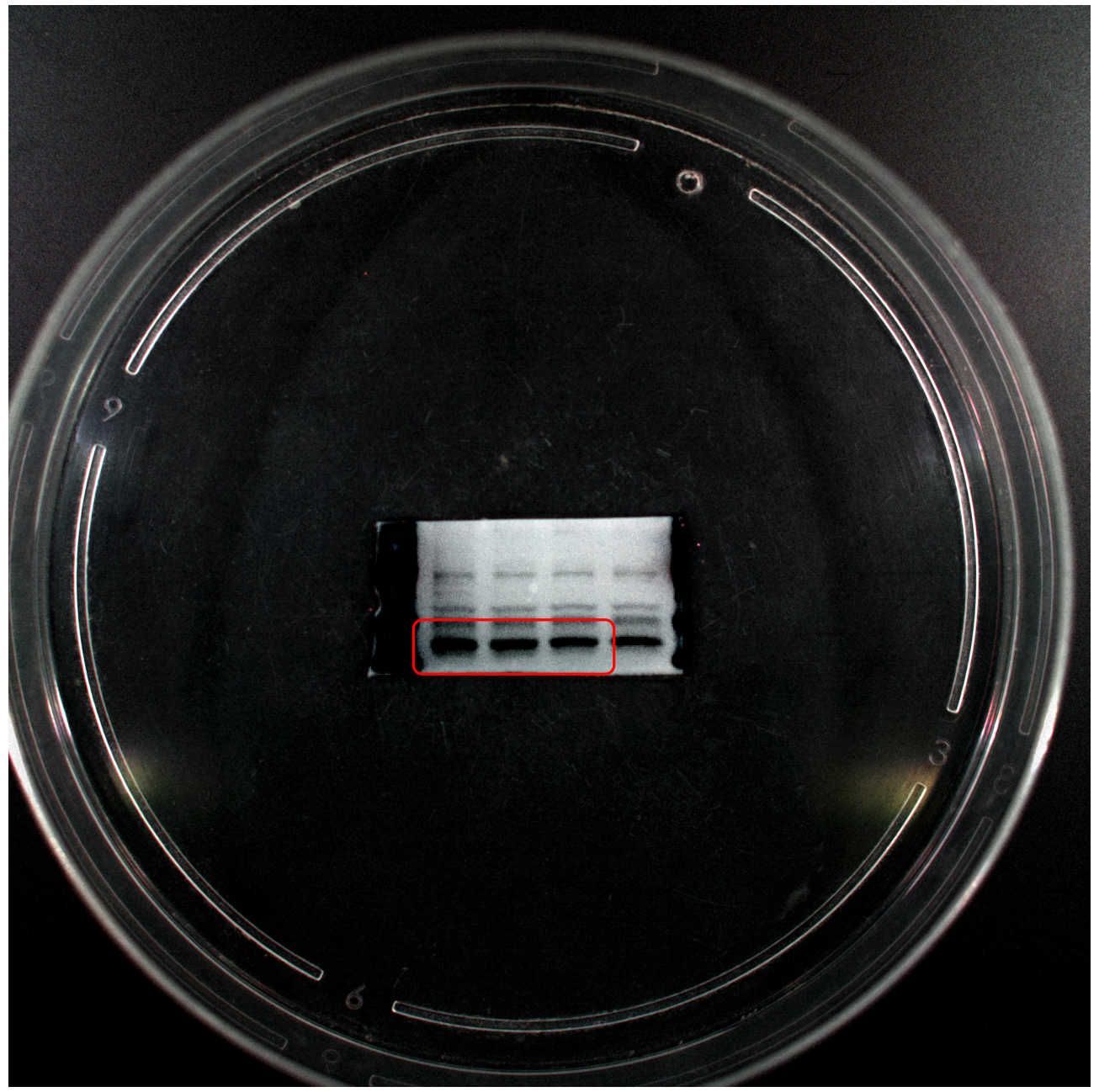

p-MDM2

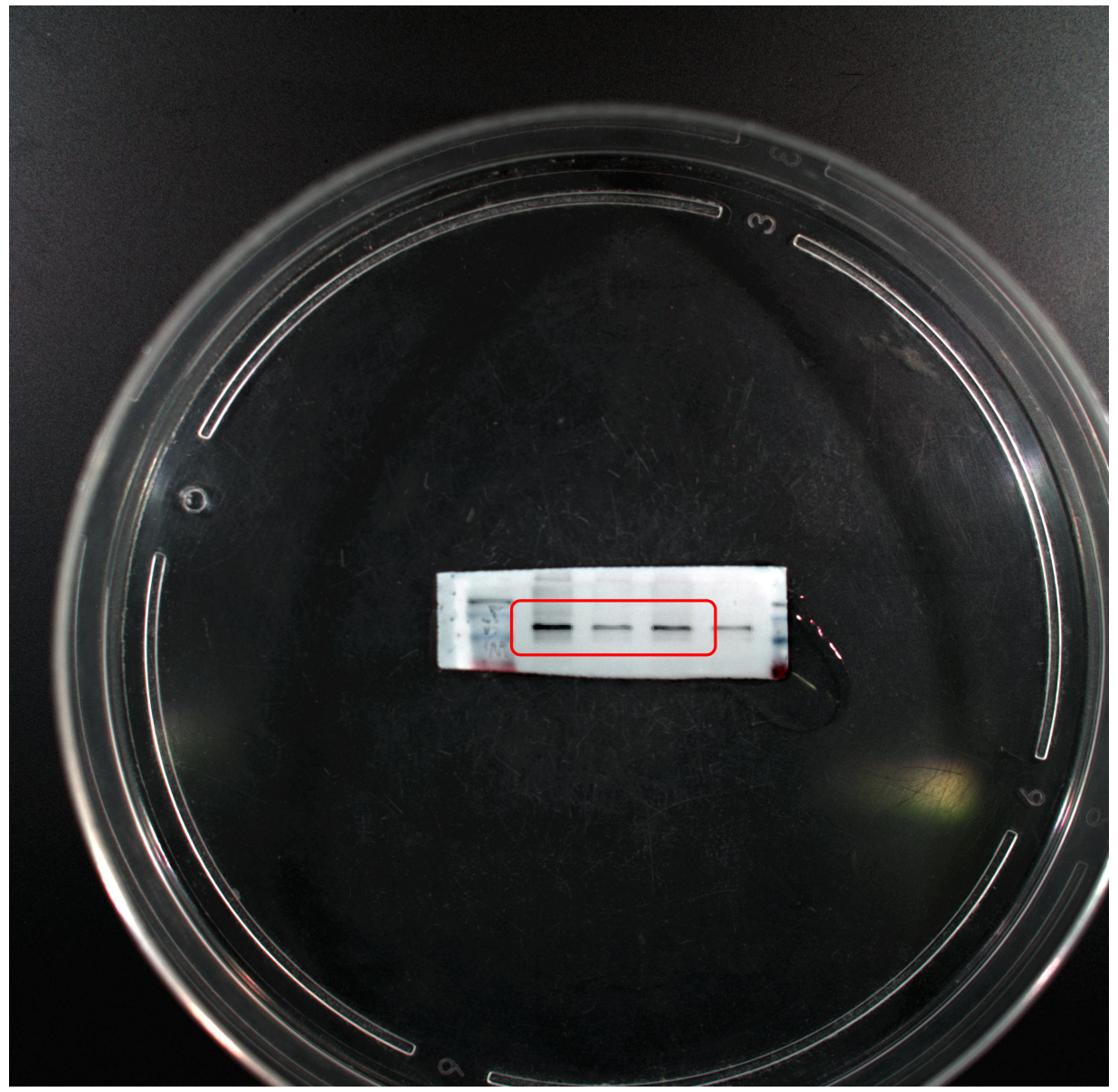

p53

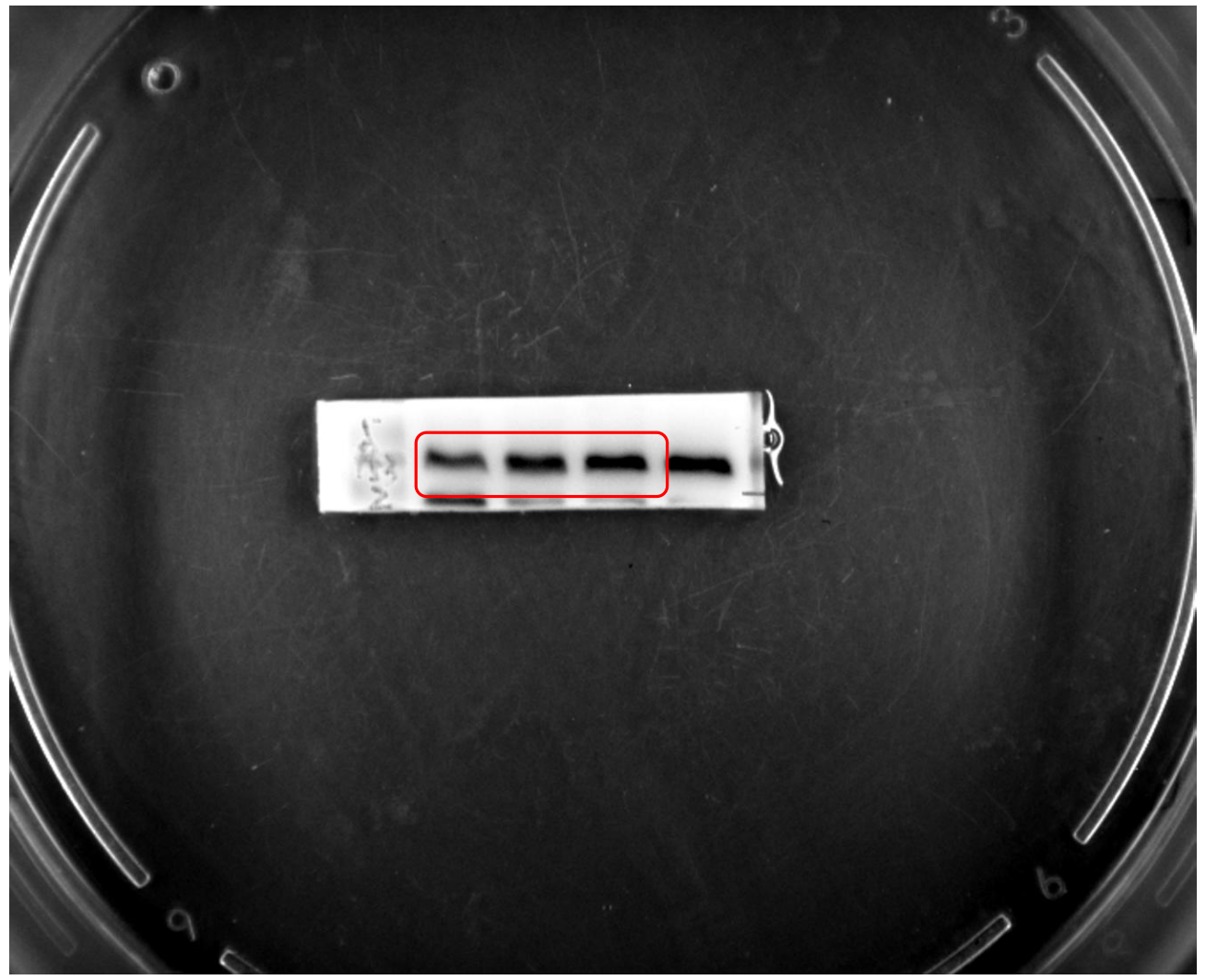

GAPDH

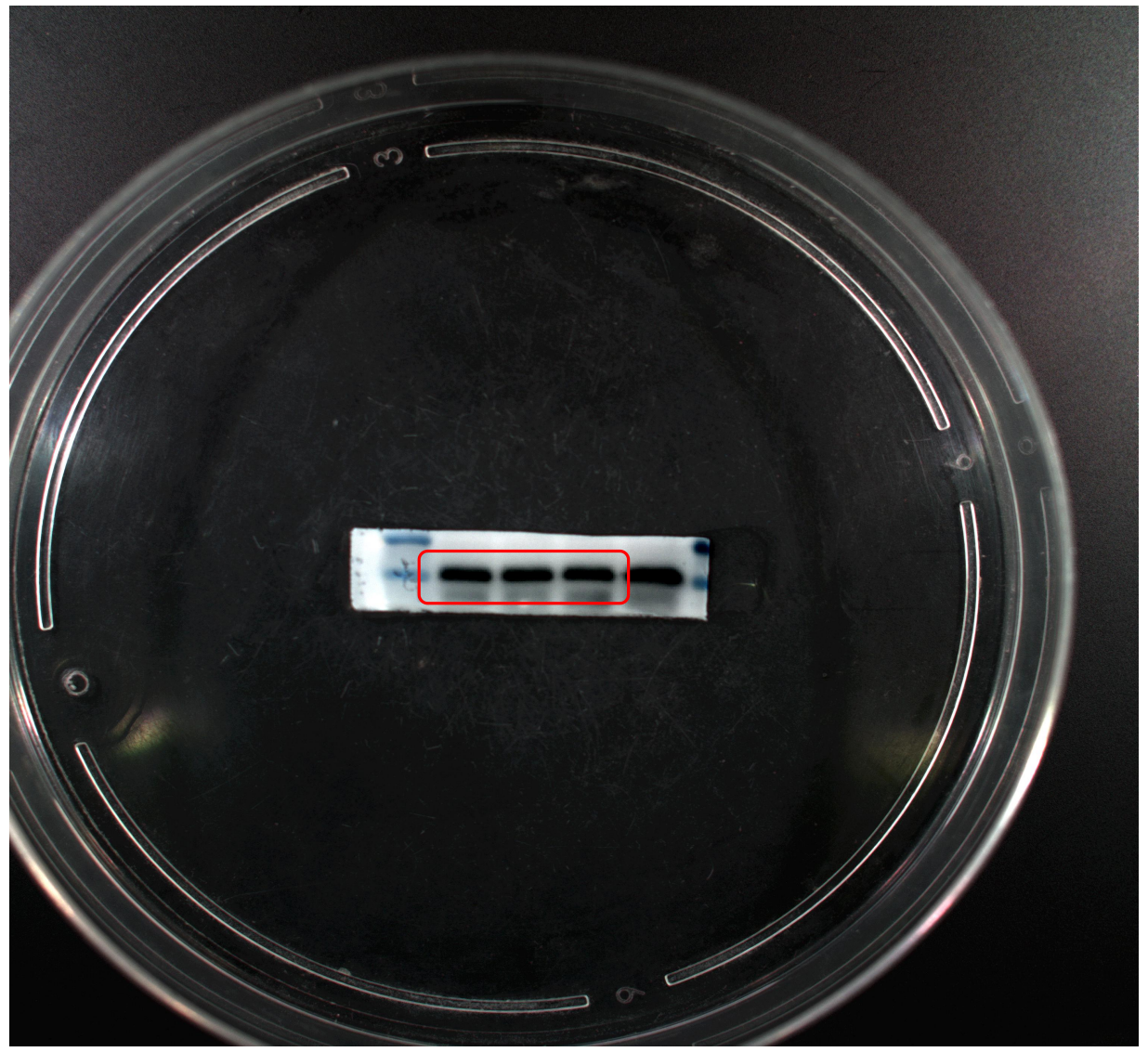

Figure 2A（from left to right: 0μM, 80μM,100μM）

**HONE1**

MDM2

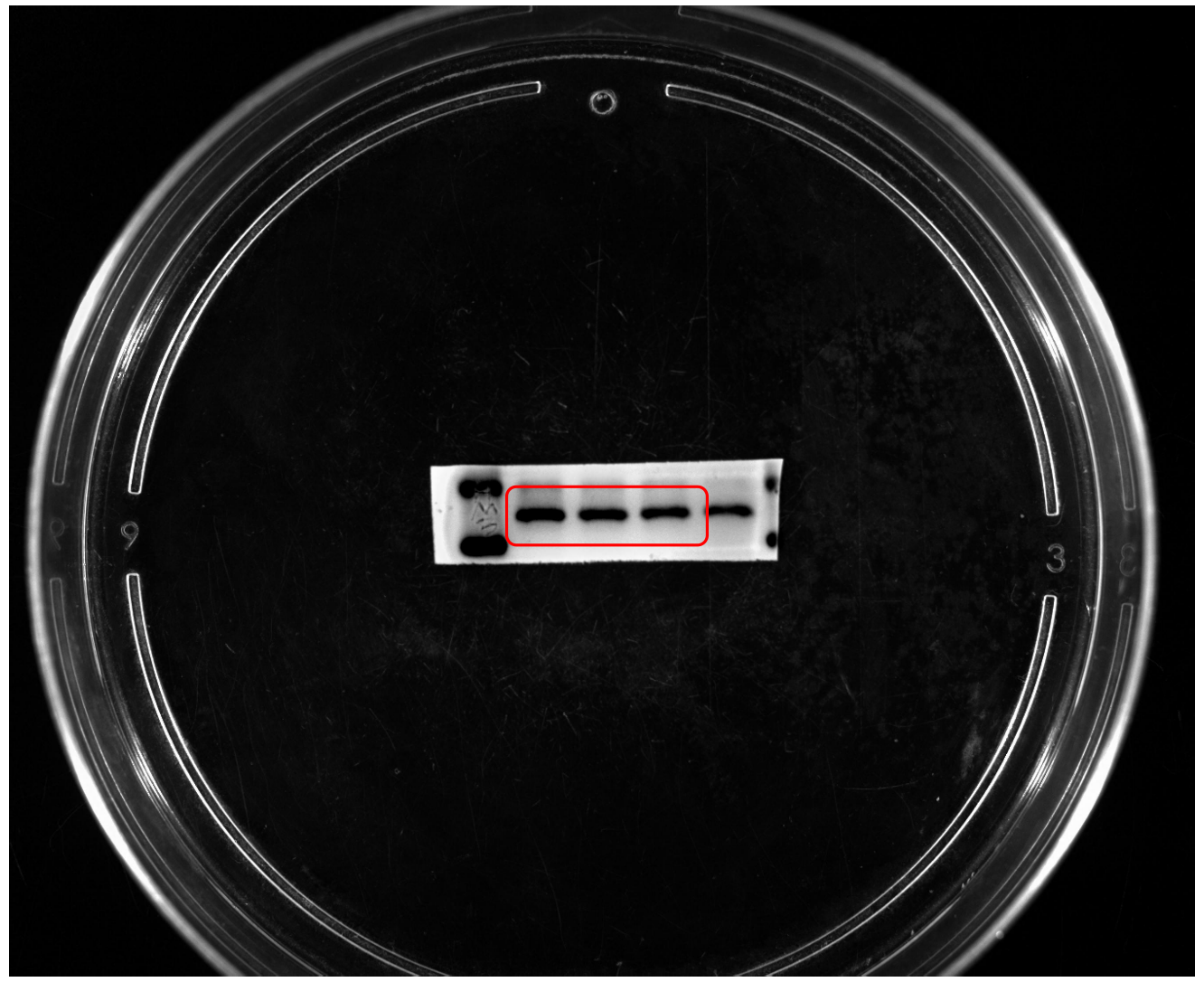

p-MDM2

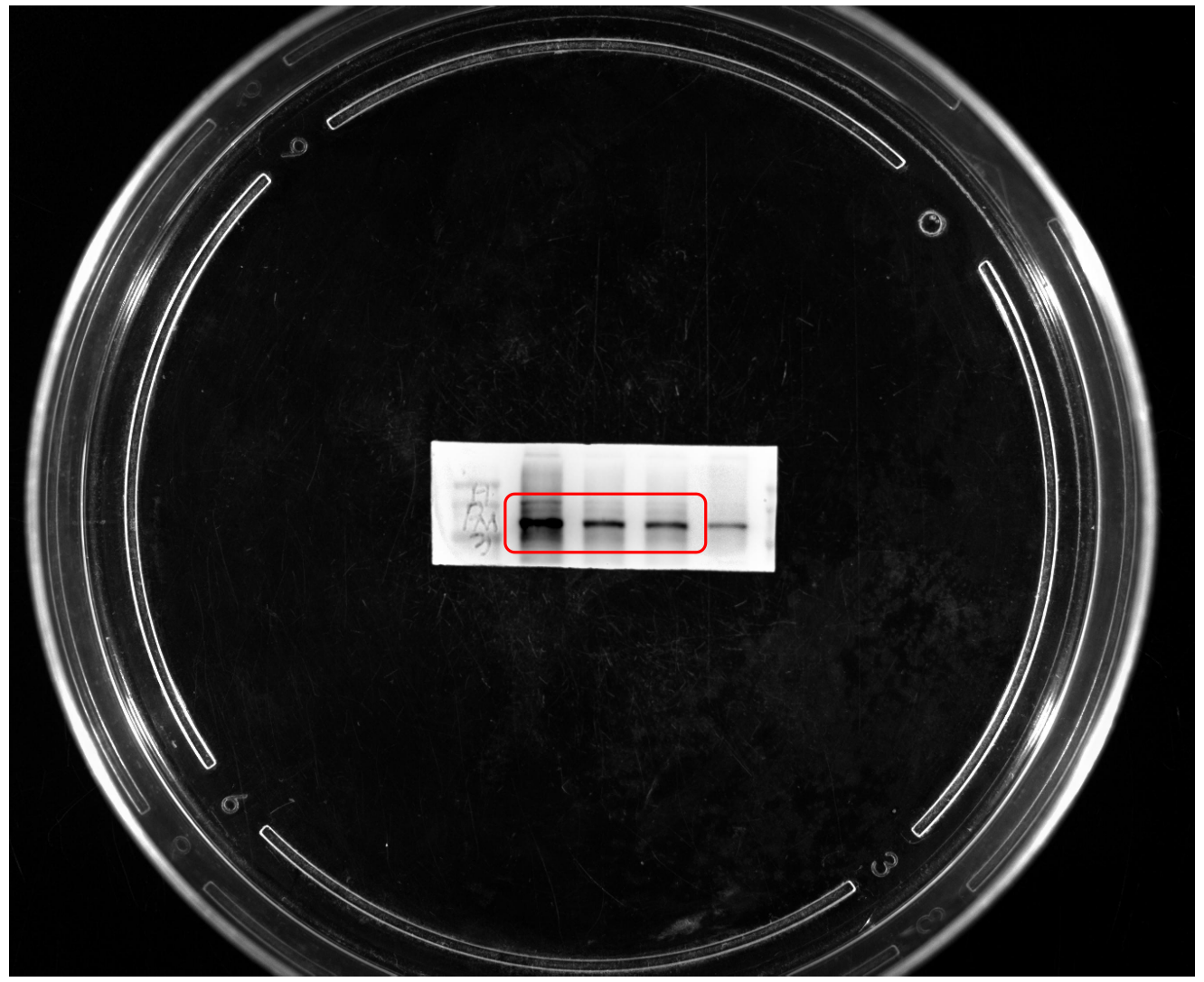

p53

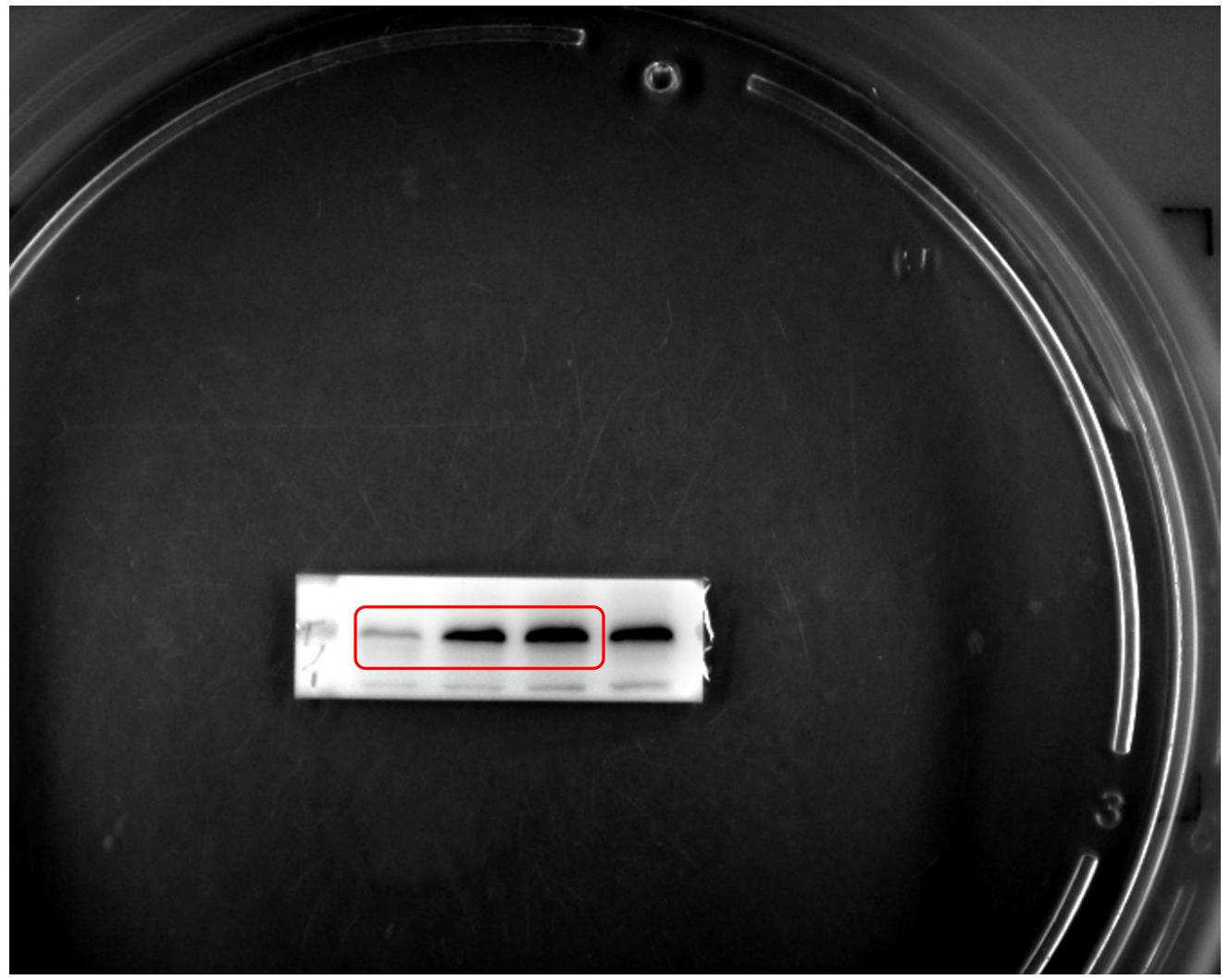

GAPDH

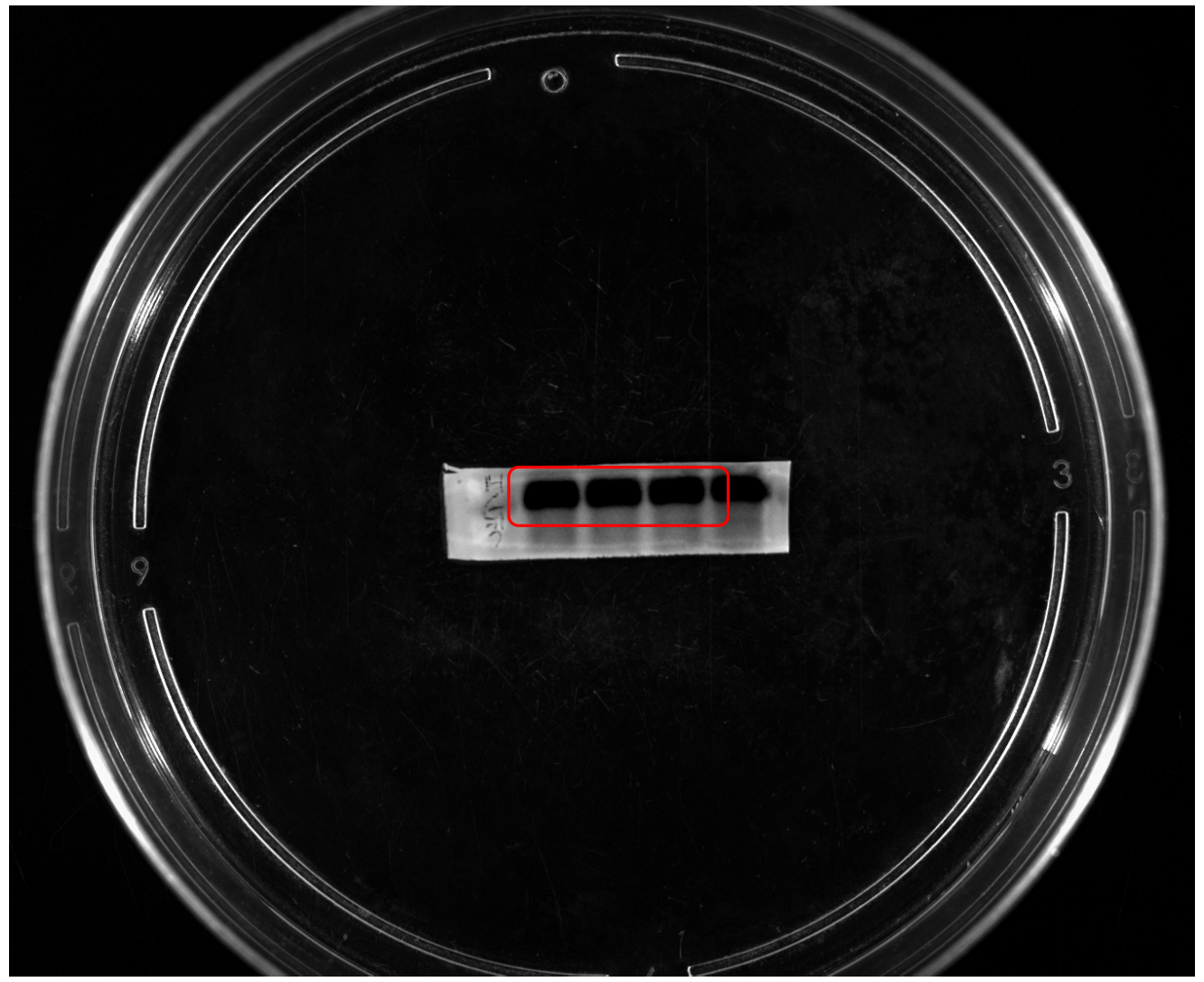

Figure 2C (from left to right: 0μM, 80μM,100μM)

**5-8F**

p53 (cytoplasm)

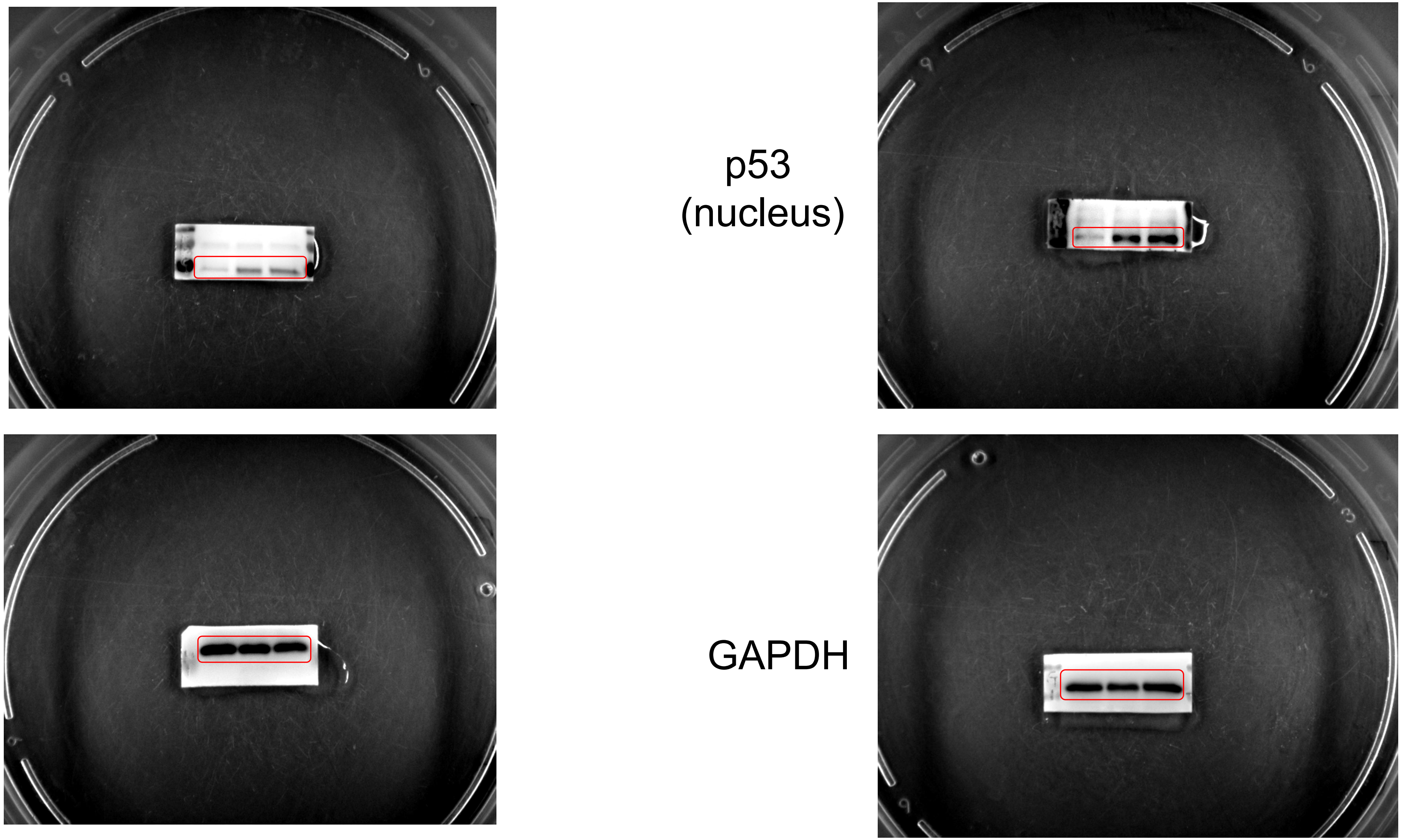

Figure 2C (from left to right: 0μM, 80μM,100μM)

**HONE1**

p53
(cytoplasm)

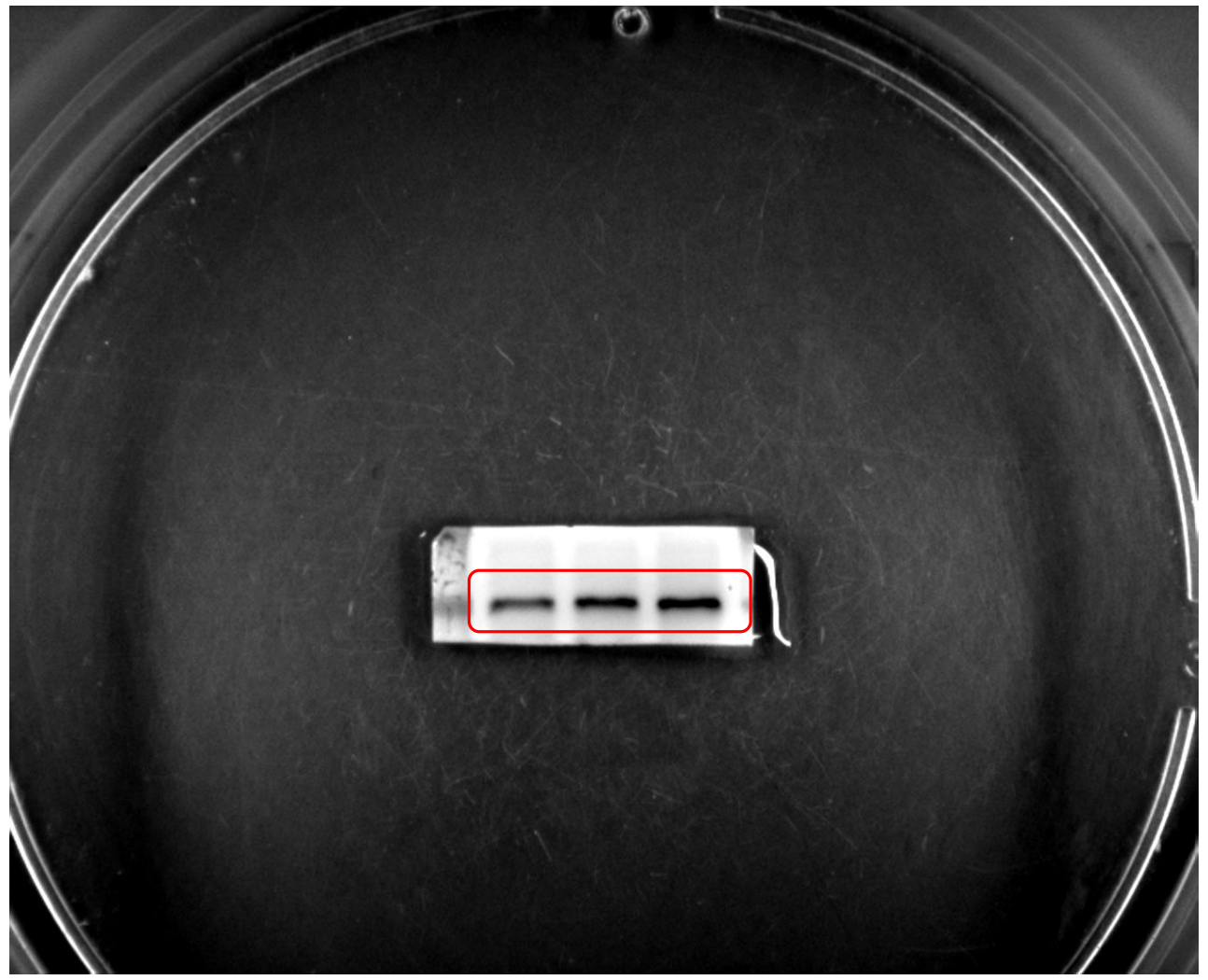

p53
(nucleus)

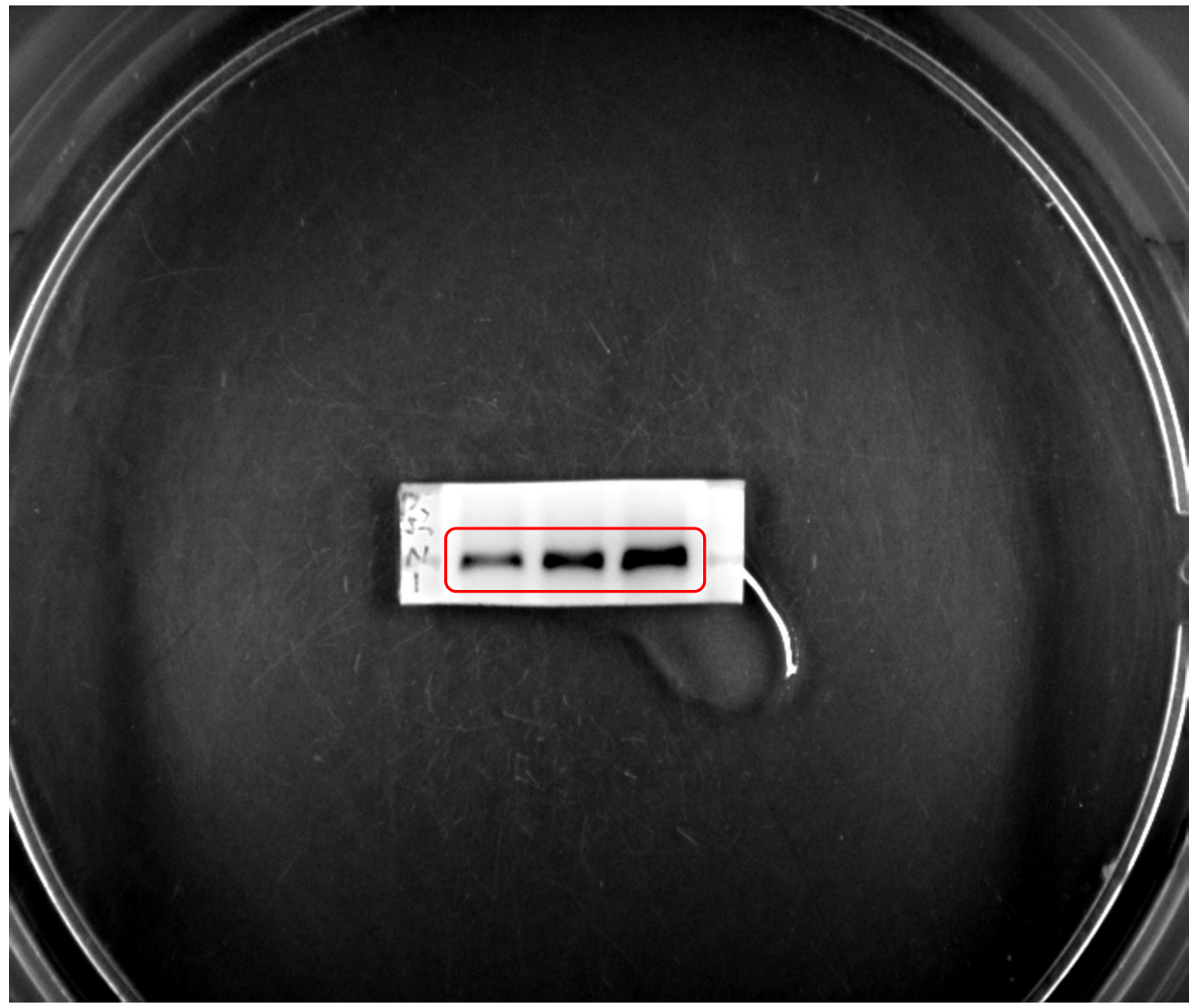

H3

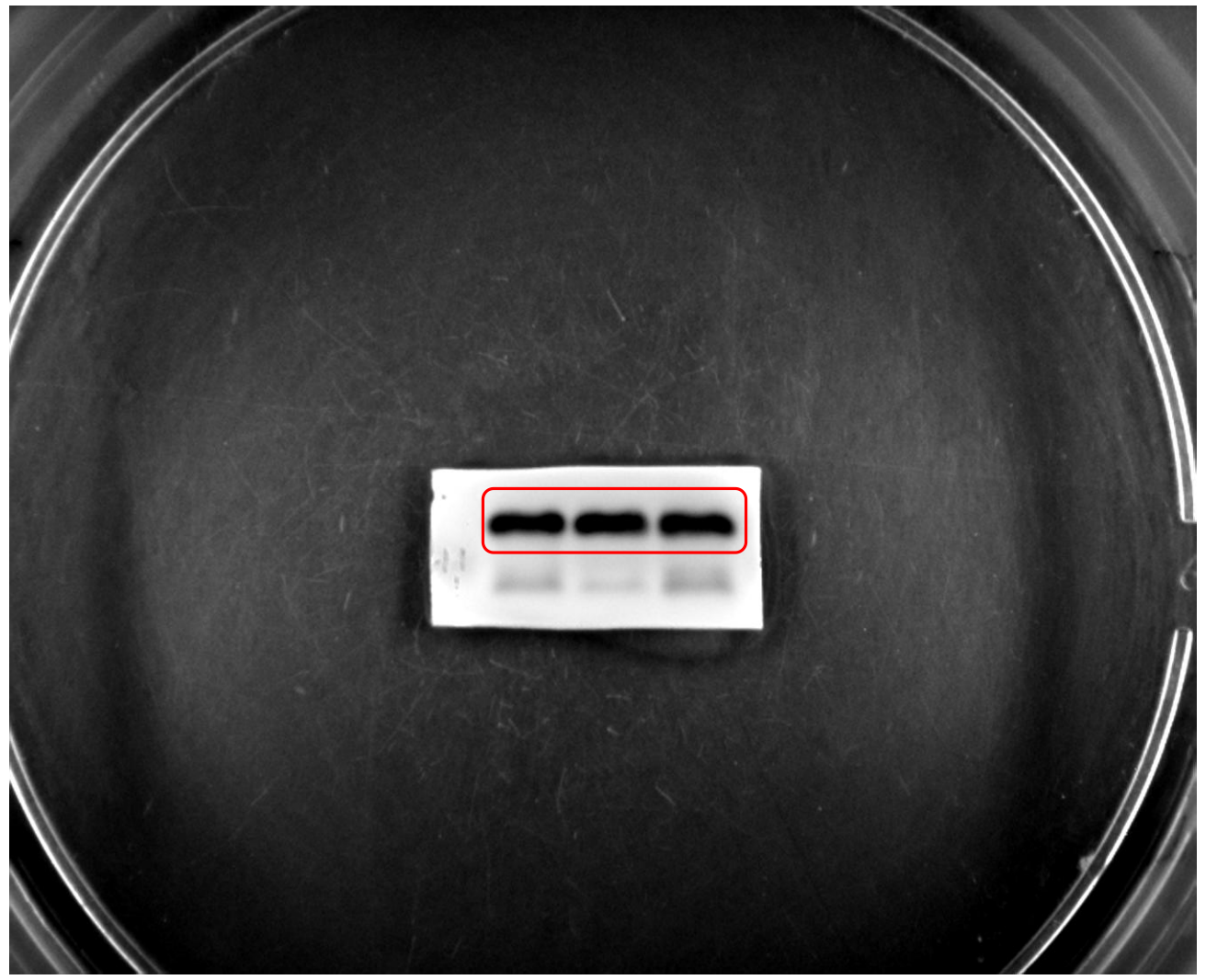

GAPDH

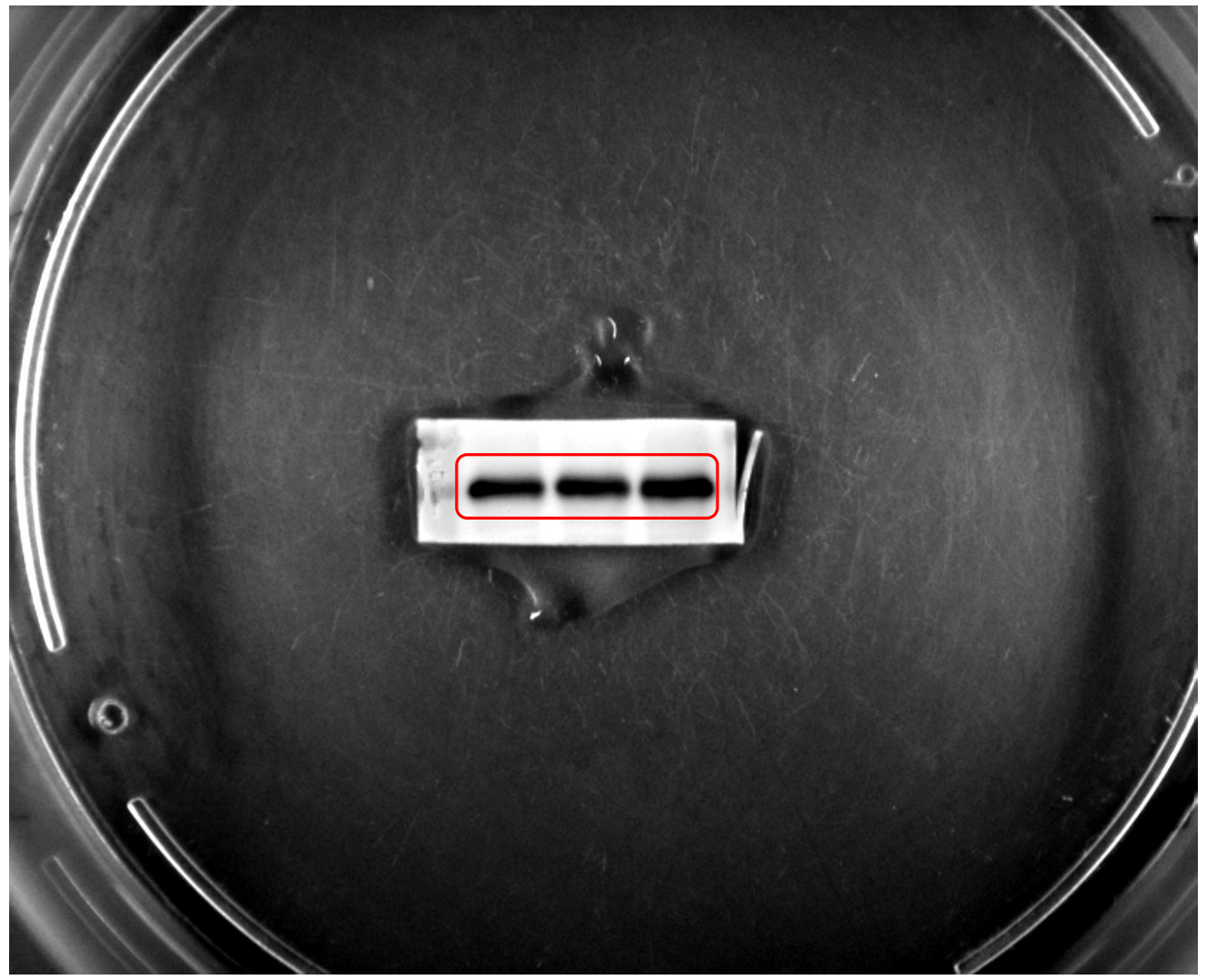

Figure 2D (from left to right: 0μM, 100μM)

**5-8F**

Ub

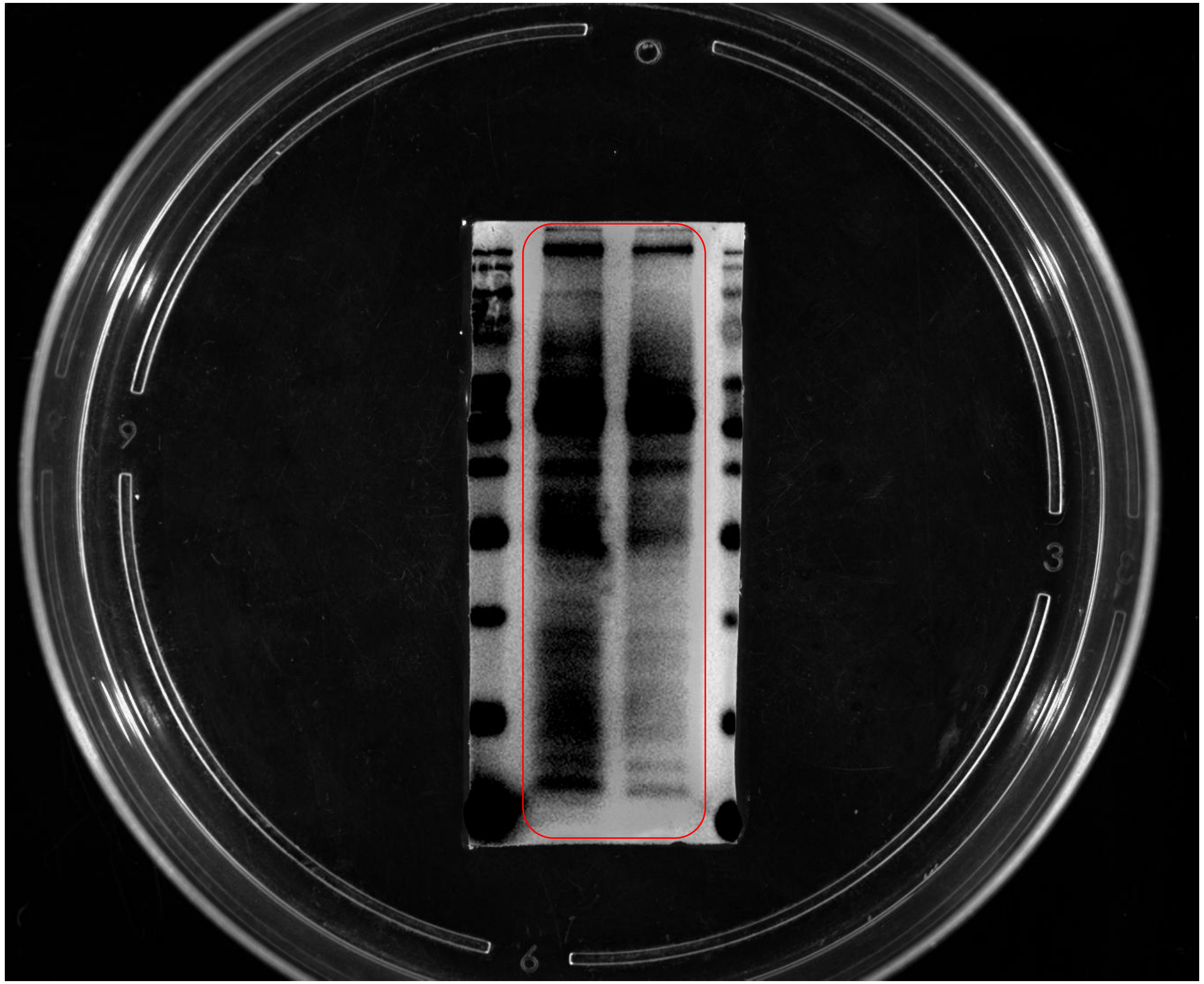

p53

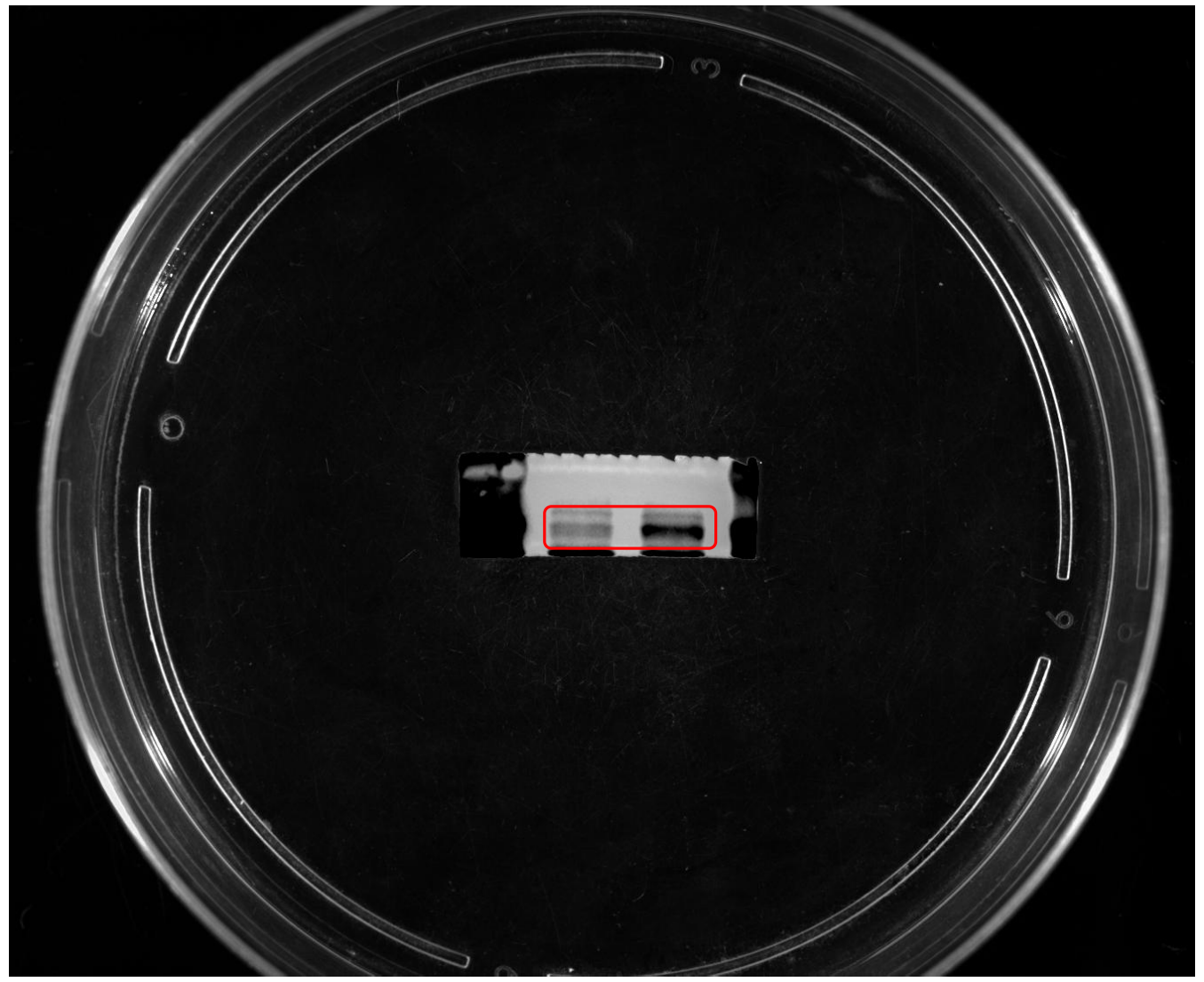

GAPDH

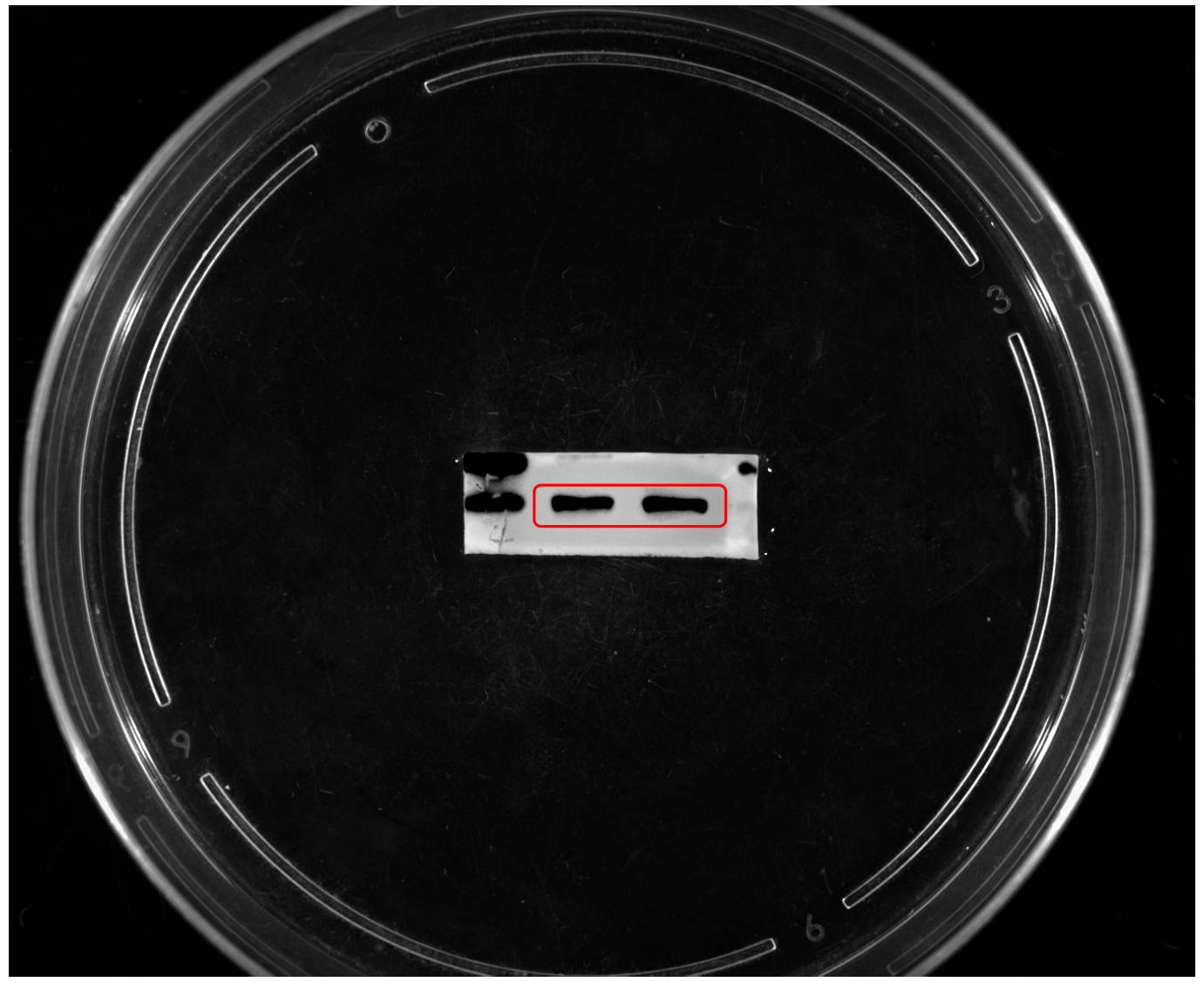

Figure 2D (from left to right: 0μM, 100μM)

**HONE1**

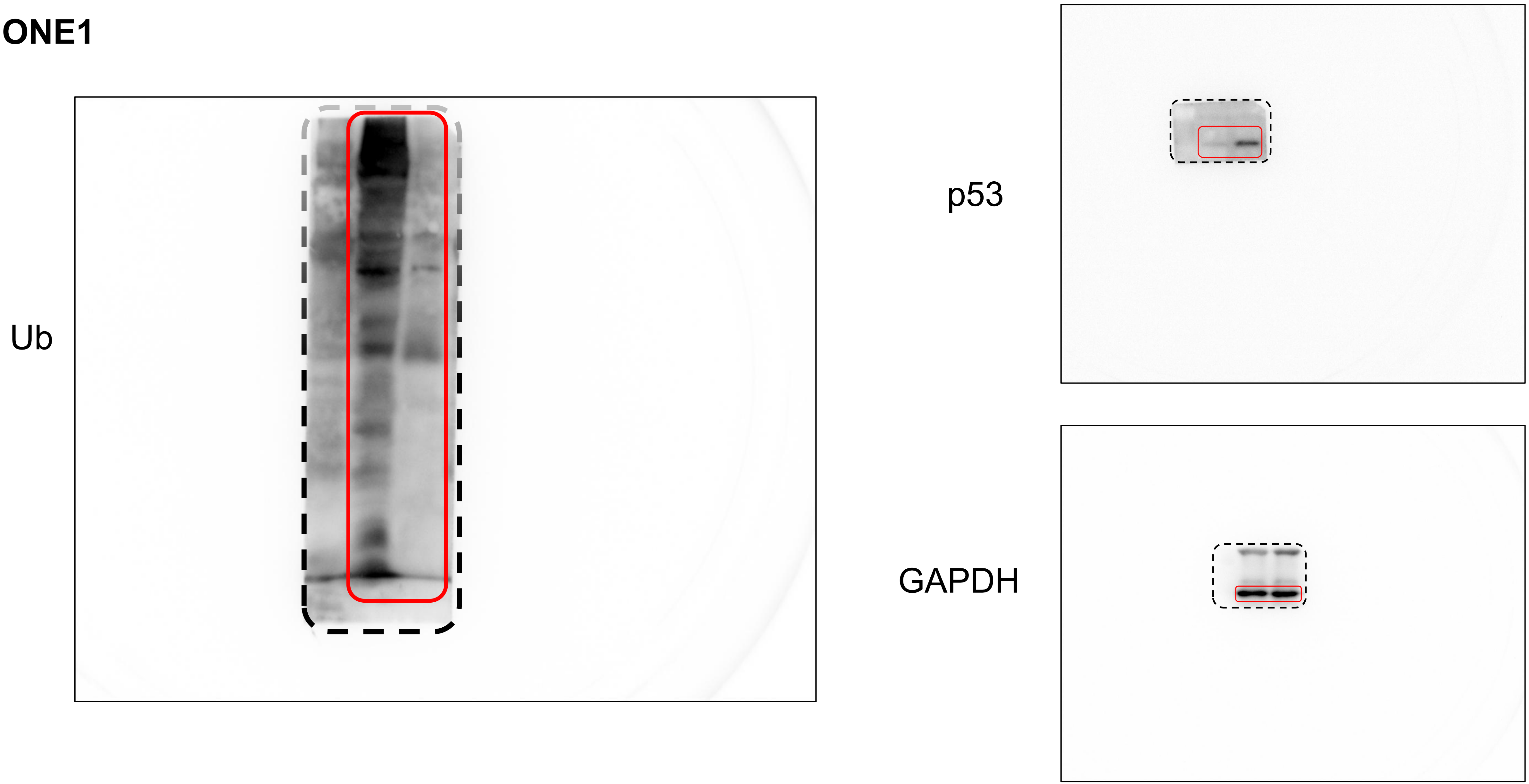

Figure 2E

**5-8F**

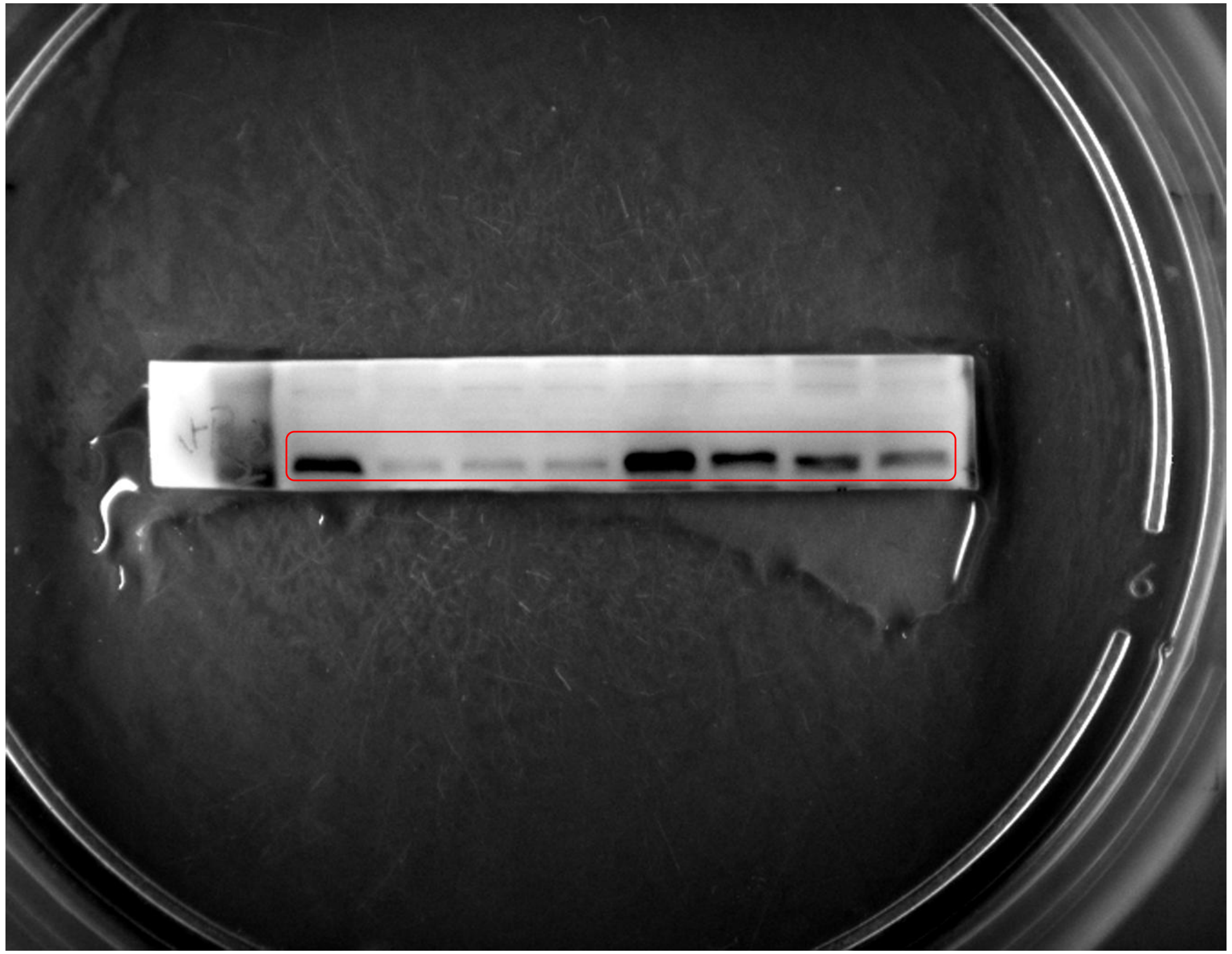


p53

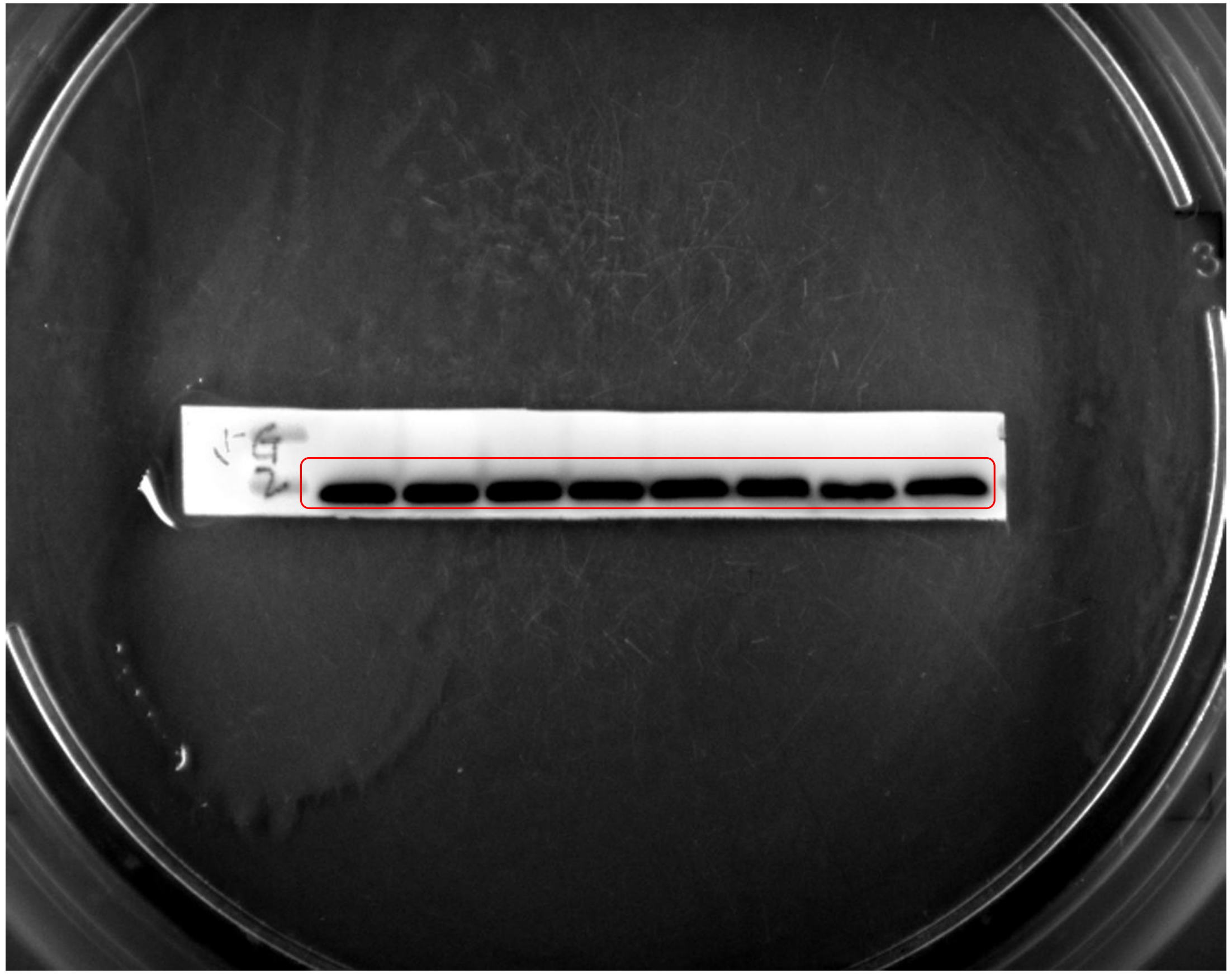


GAPDH

# Figure 2E

## HONE1

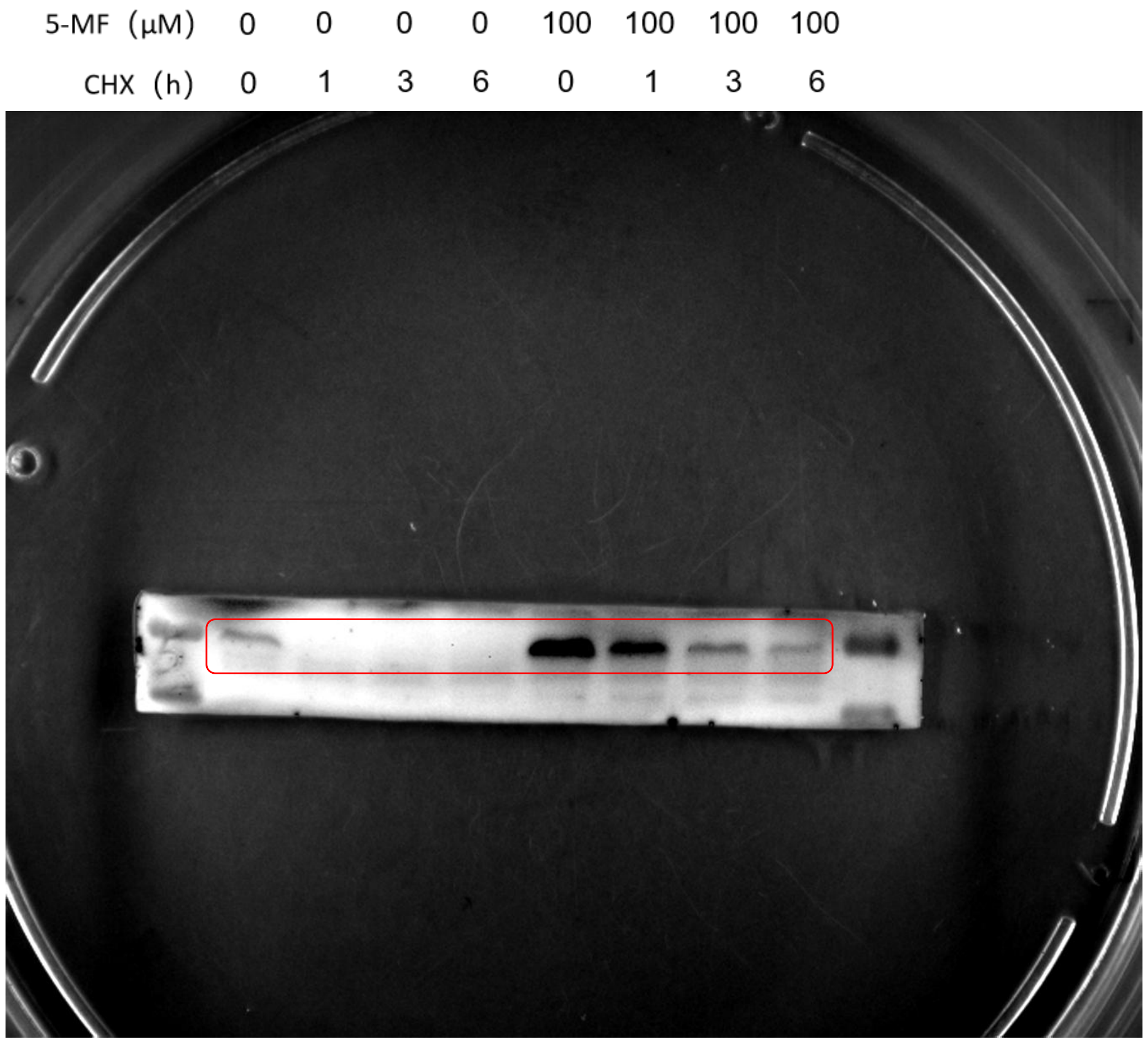


p53

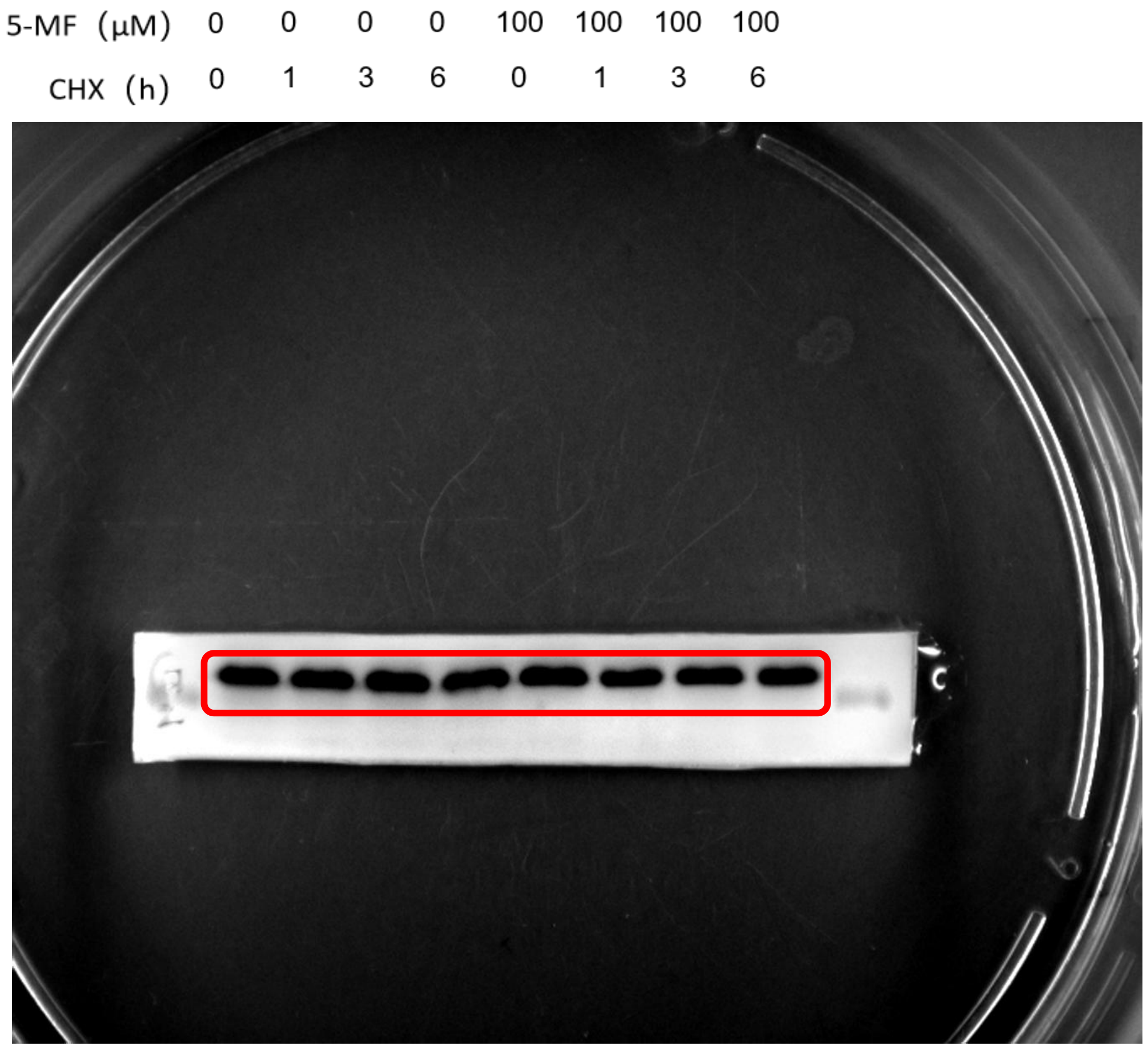


GAPDH

Figure 3D (from left to right: 0μM, 80μM,100μM)

**5-8F**

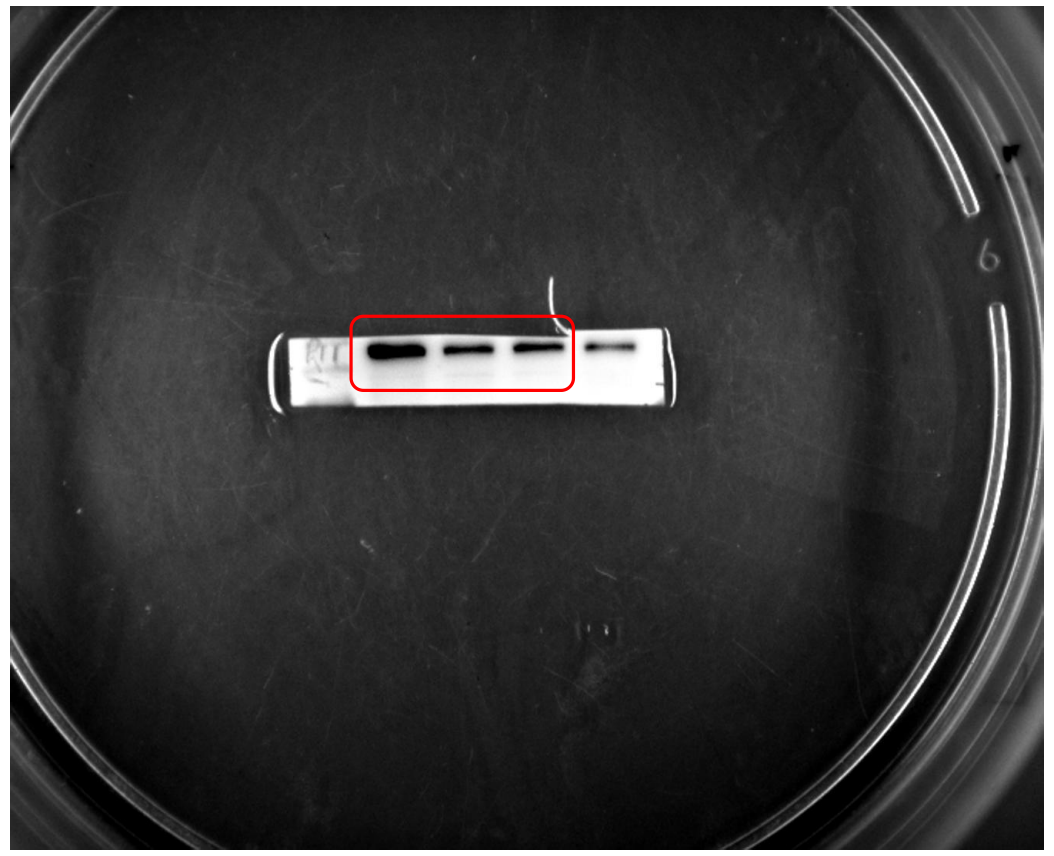

SREBP1

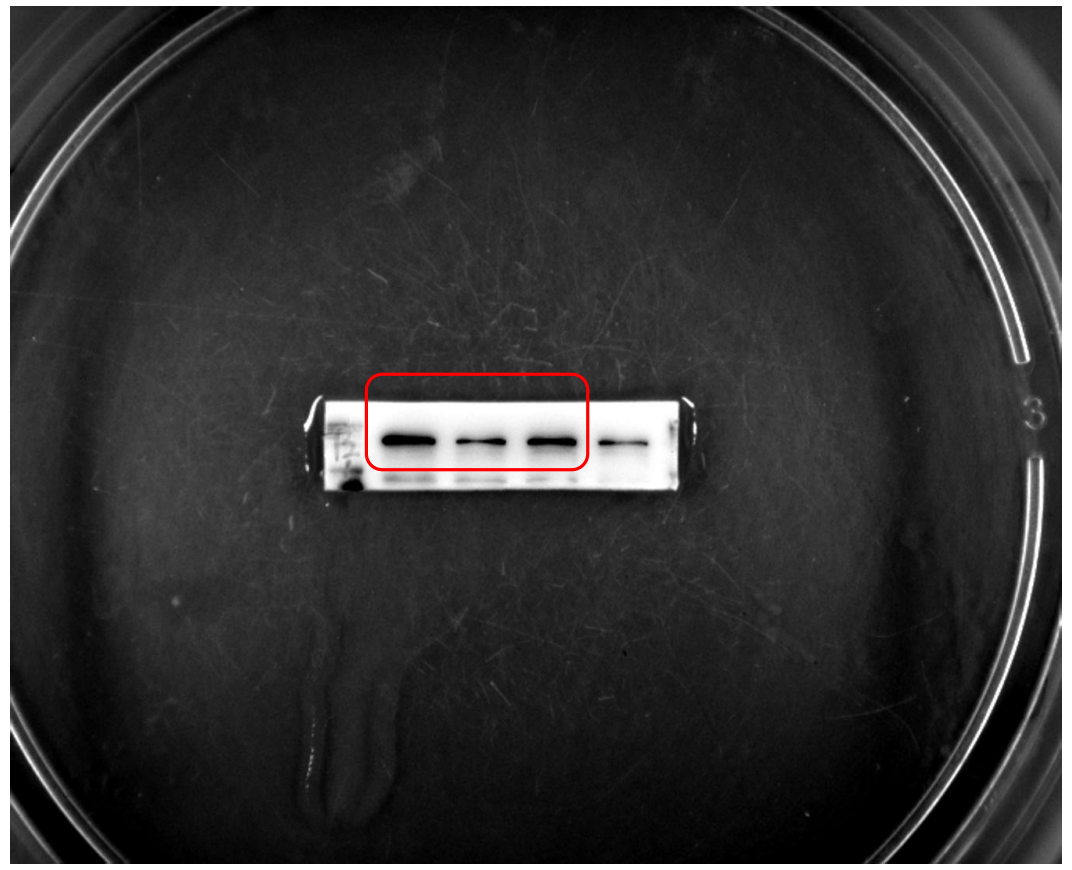

SREBP2

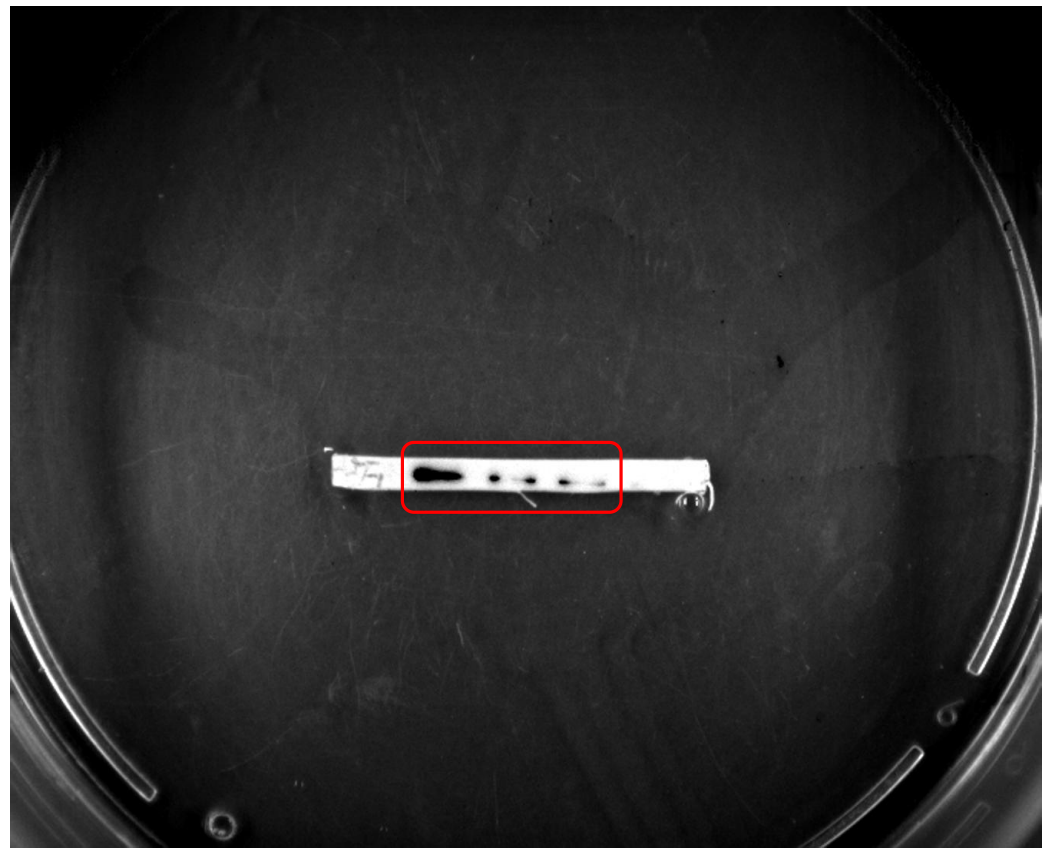

FASN

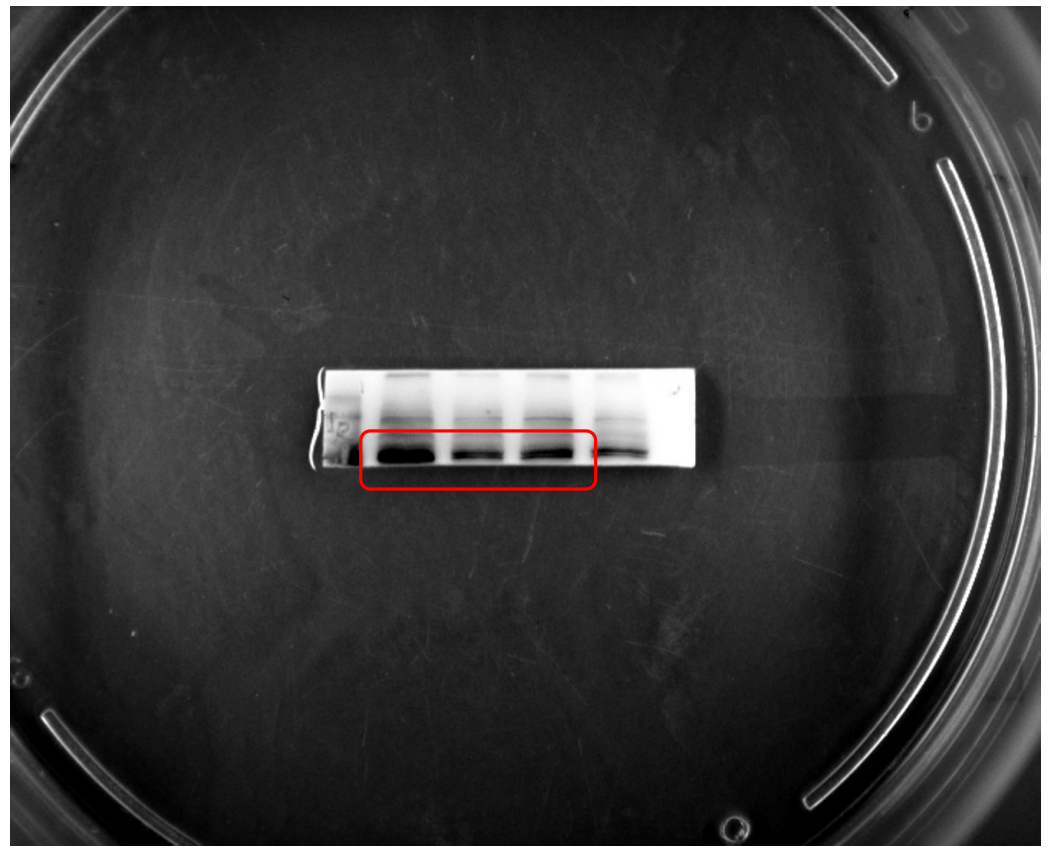

HMGCR

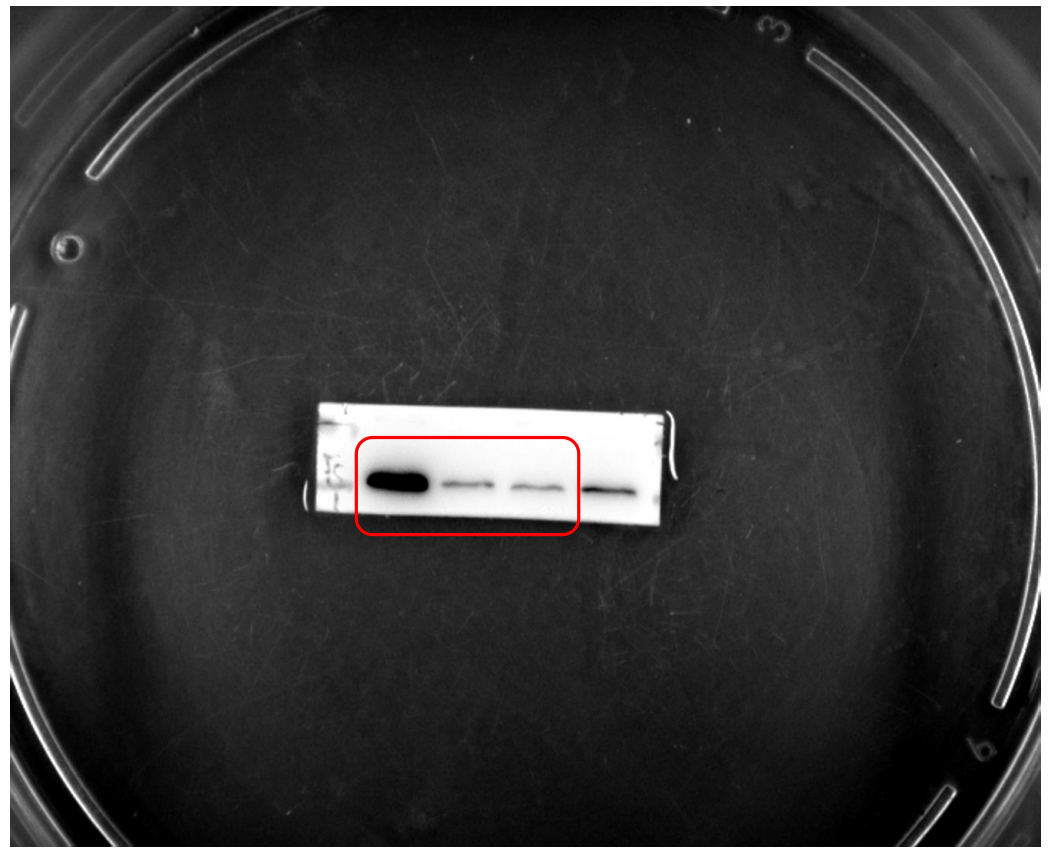

HMGCS1

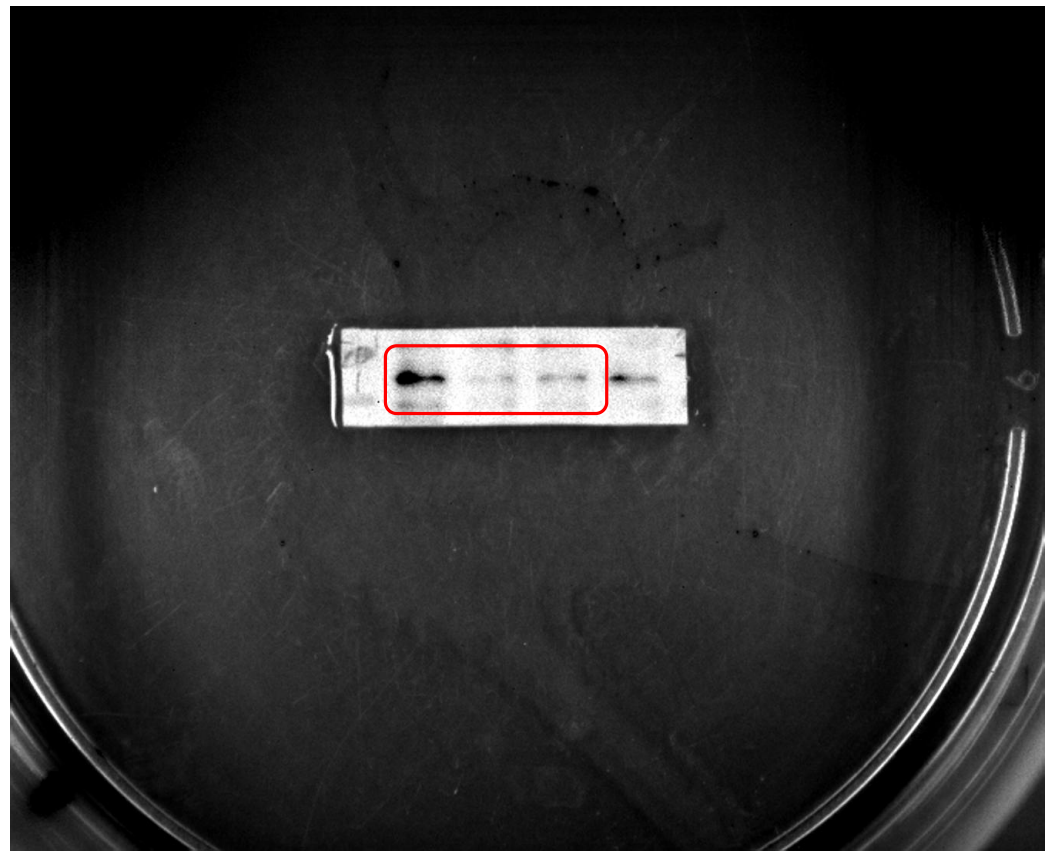

SCD

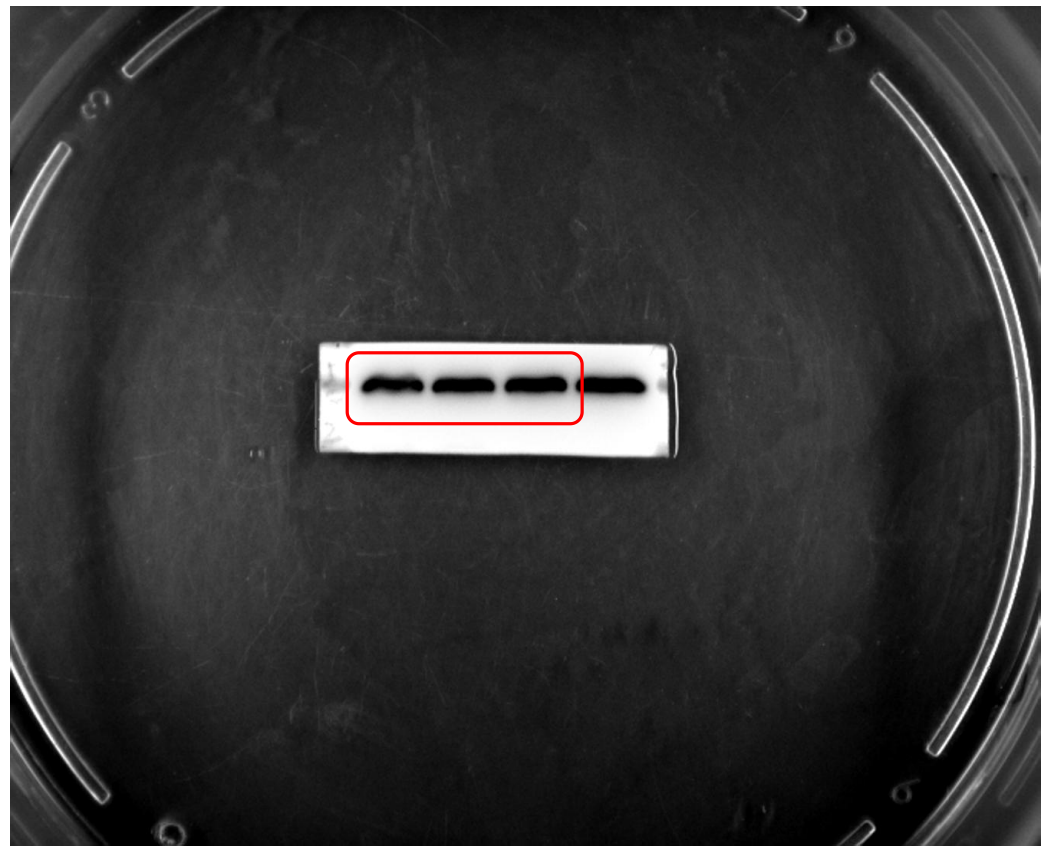

GAPDH

Figure 3D (from left to right: 0μM, 80μM,100μM)

**HONE1**

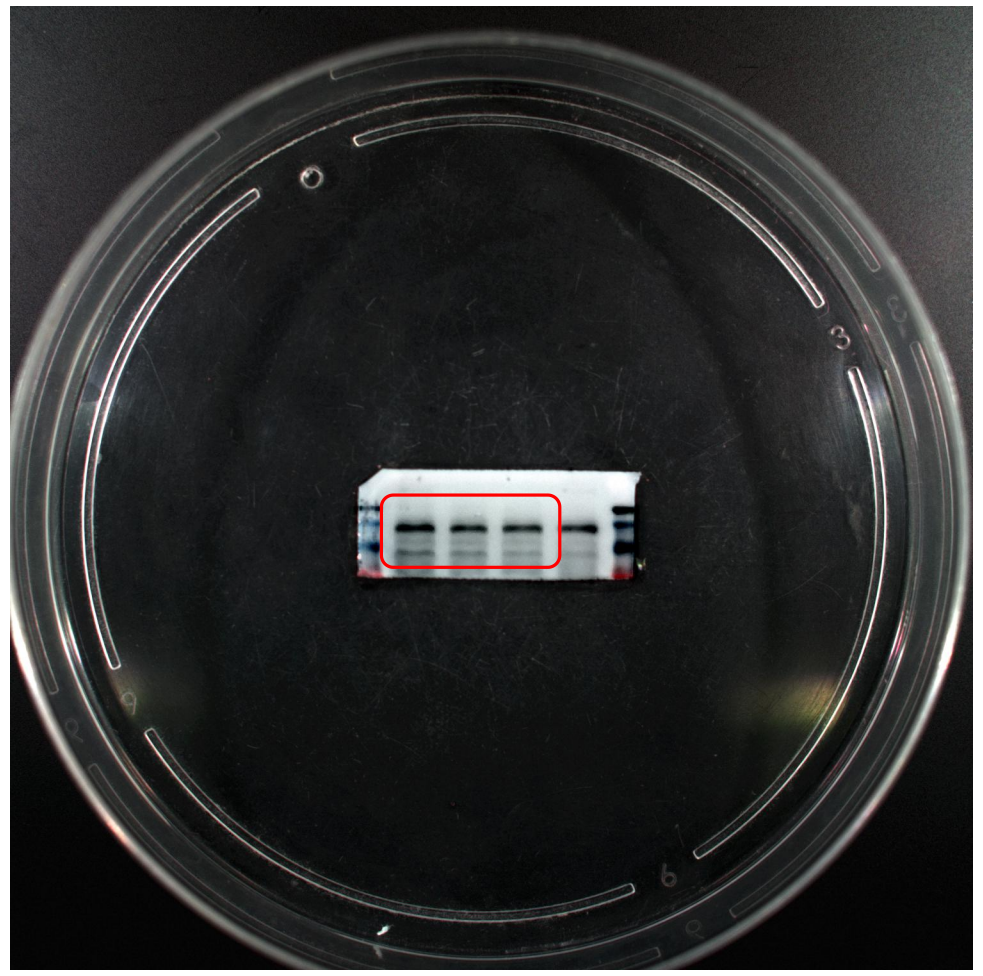

SREBP1

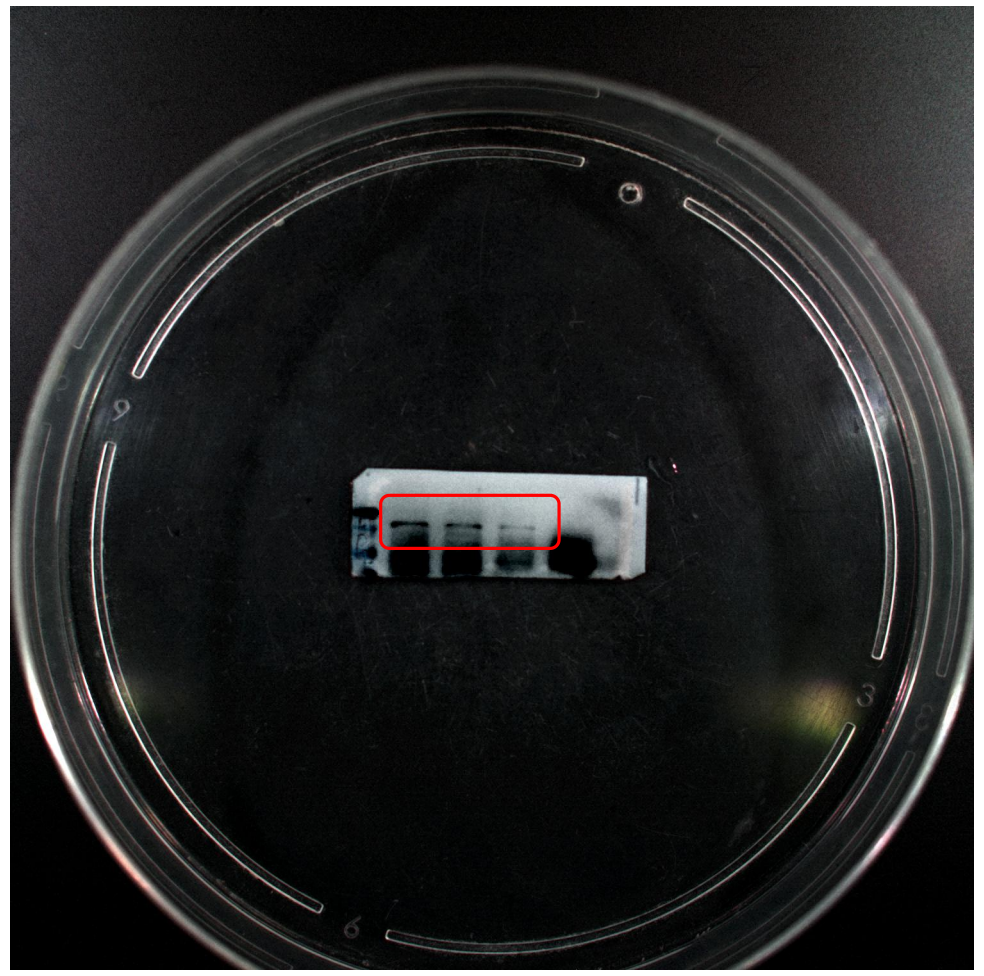

SREBP2

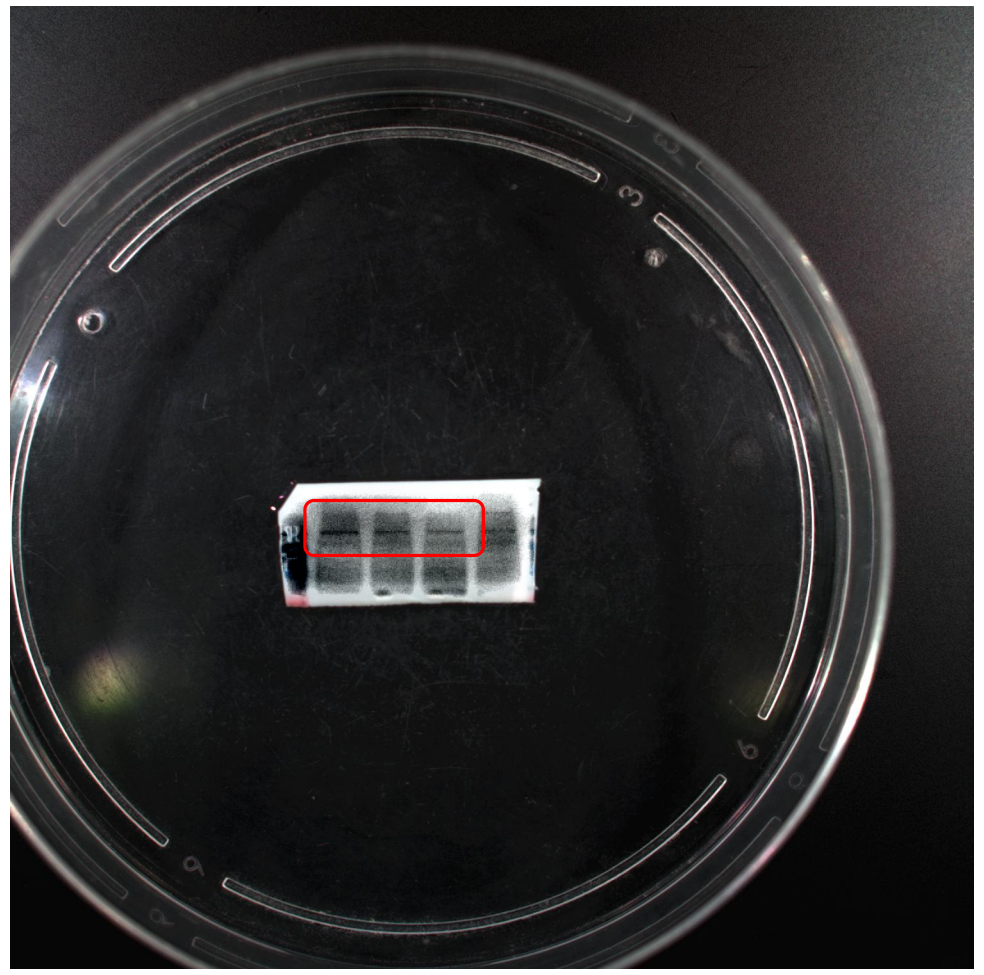

FASN

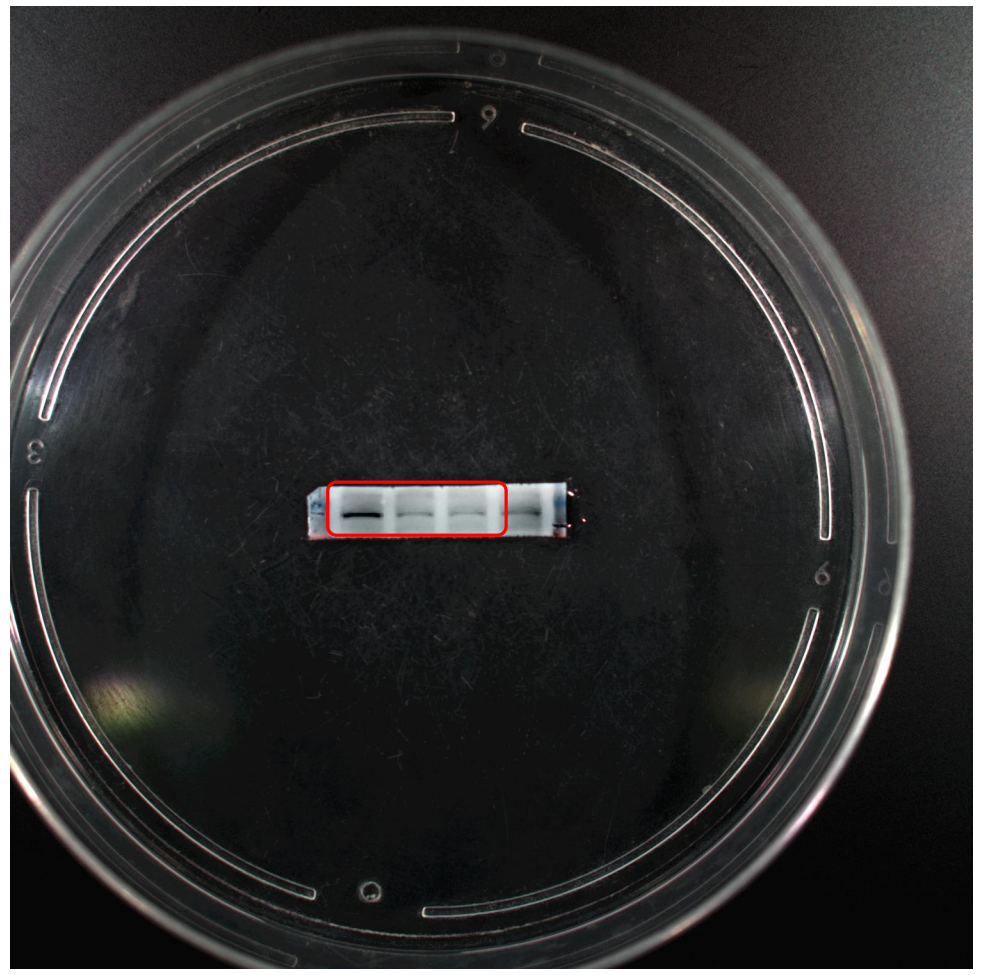

HMGCR

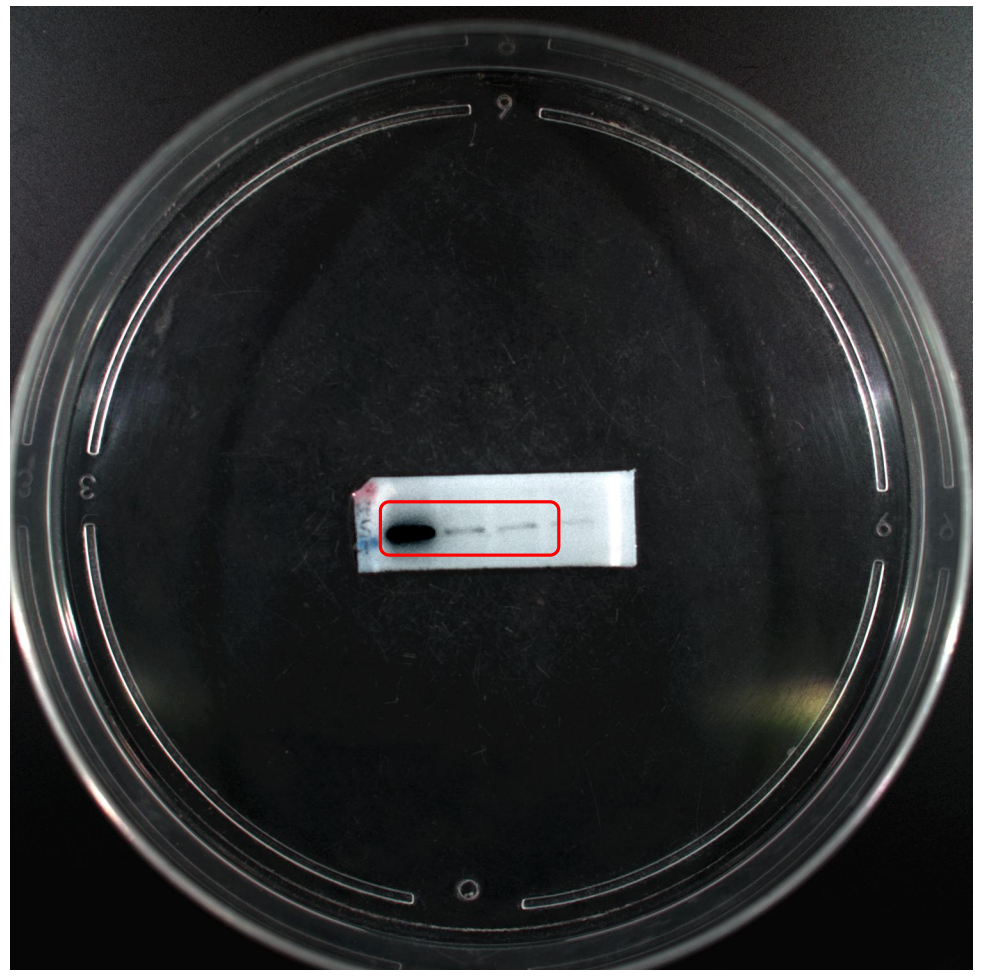

HMGCS1

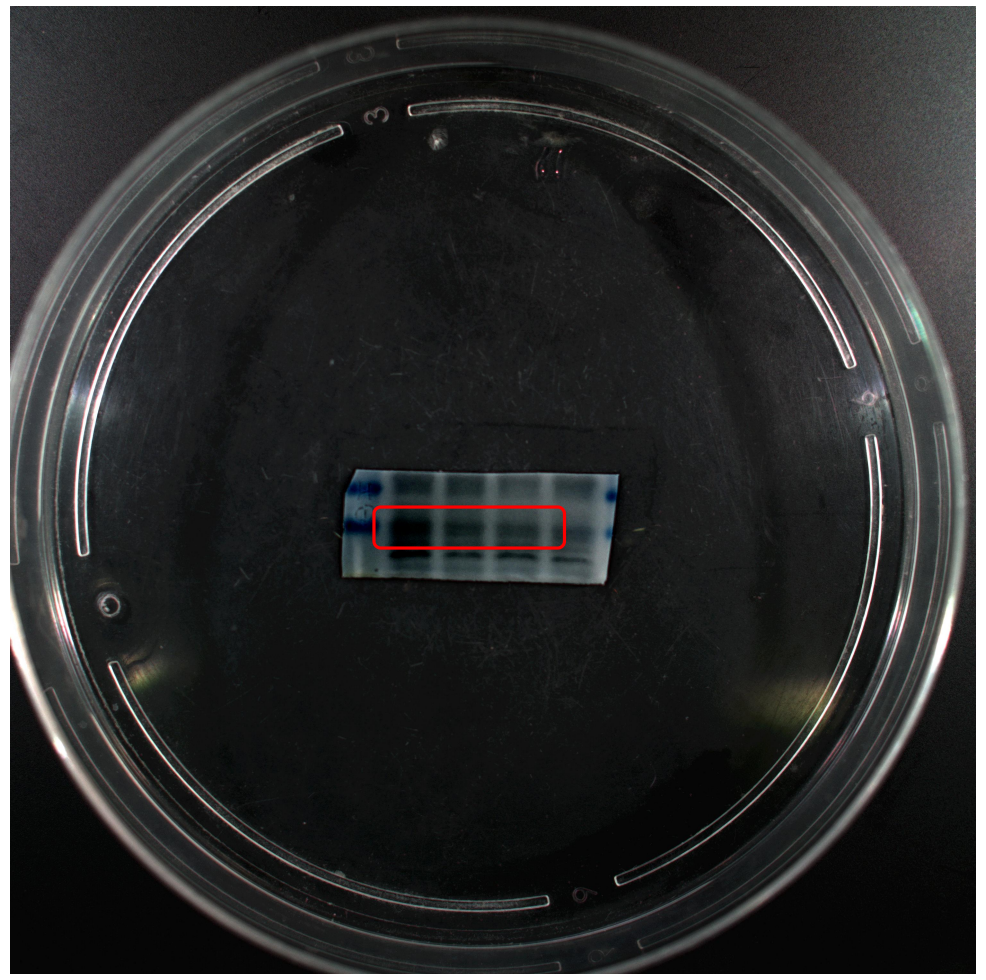

SCD

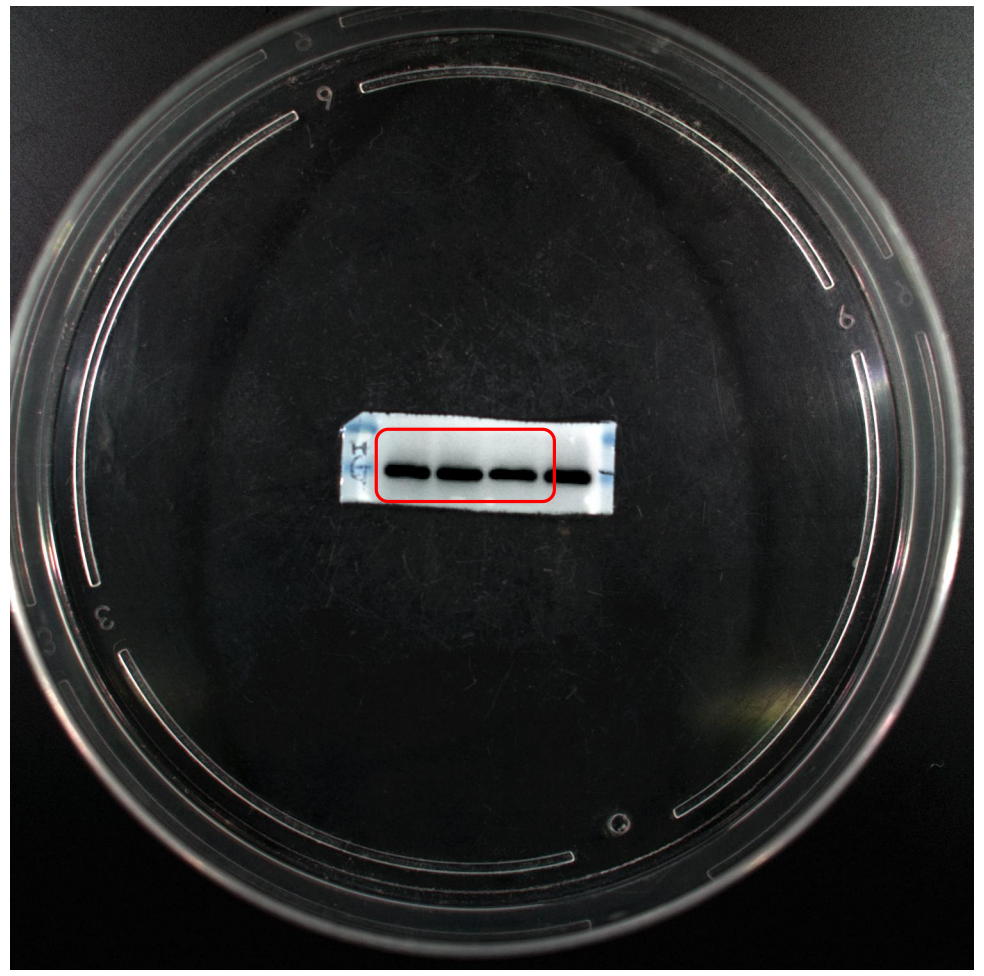

GAPDH

Figure 3E （from left to right: 0μM, 80μM,100μM）

**5-8F**

m-SREBP1

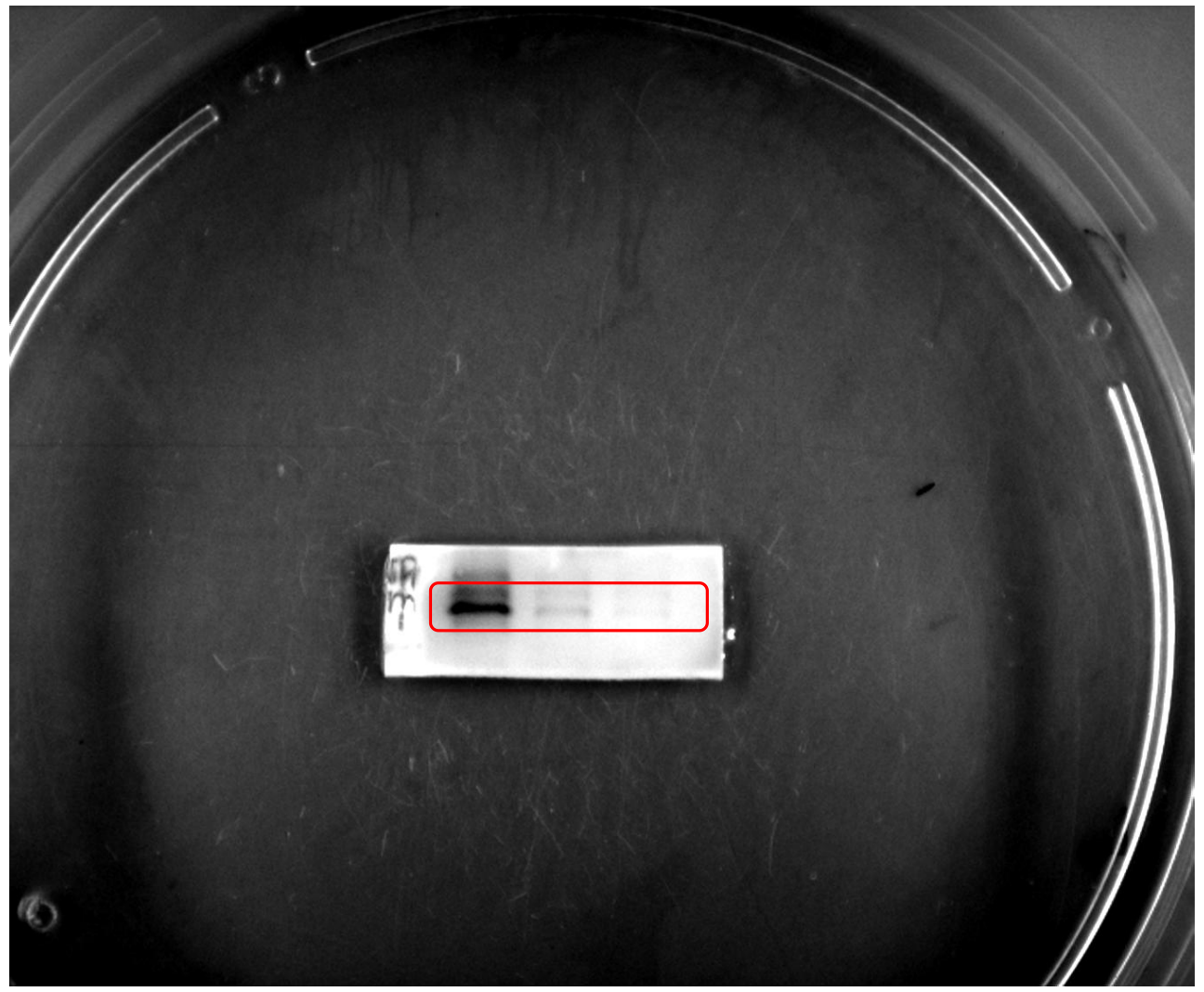

p-SREBP1

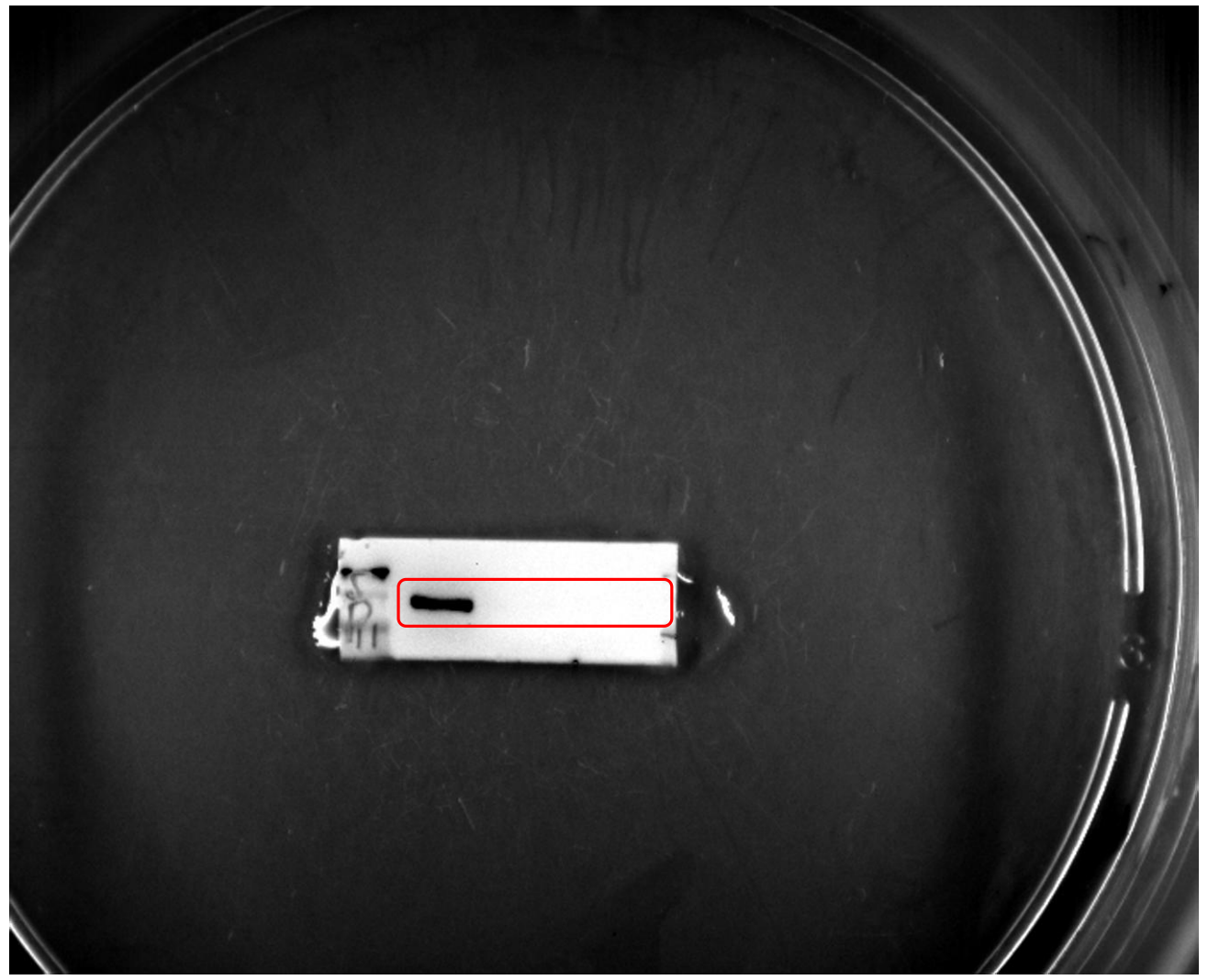

H3

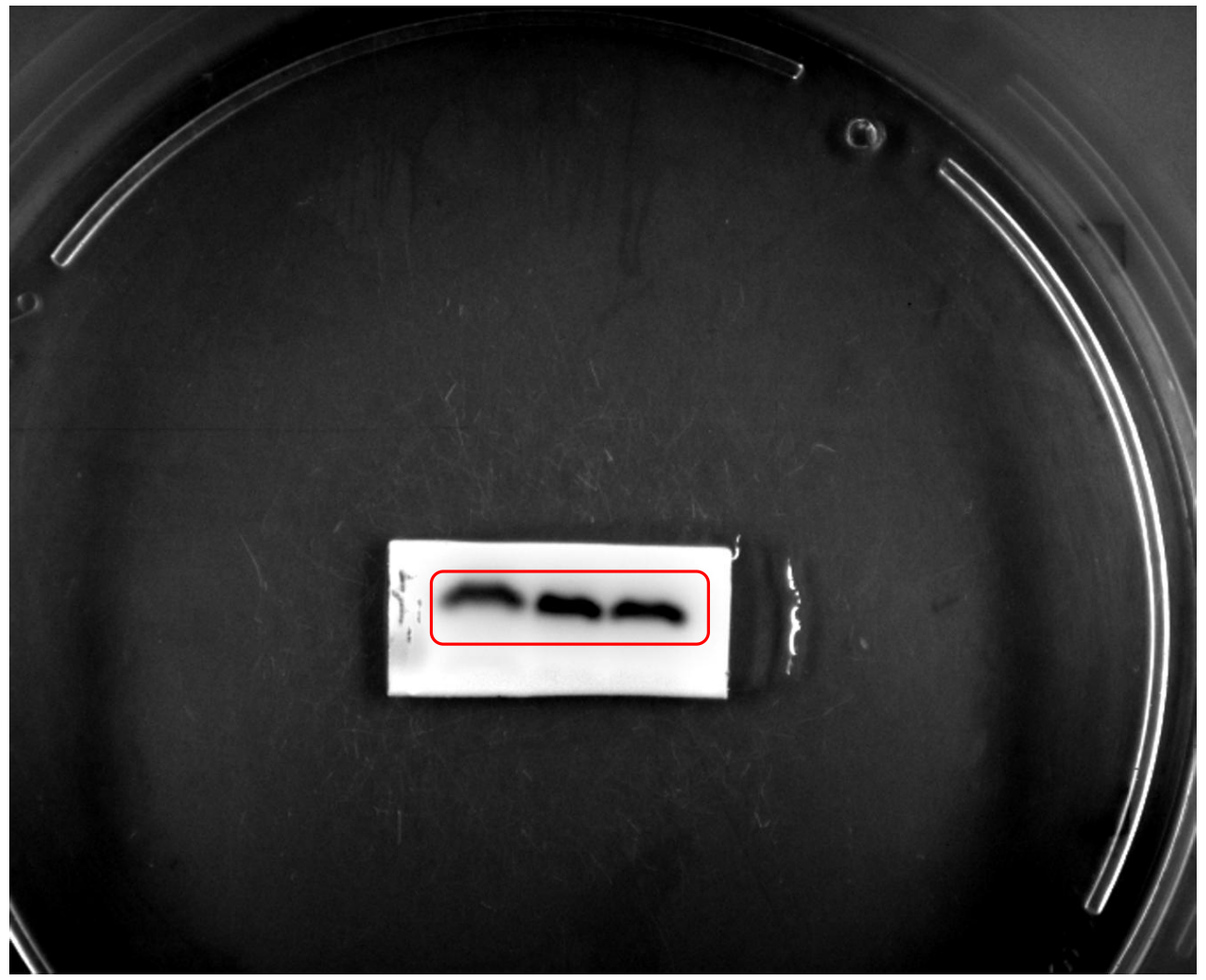

GAPDH

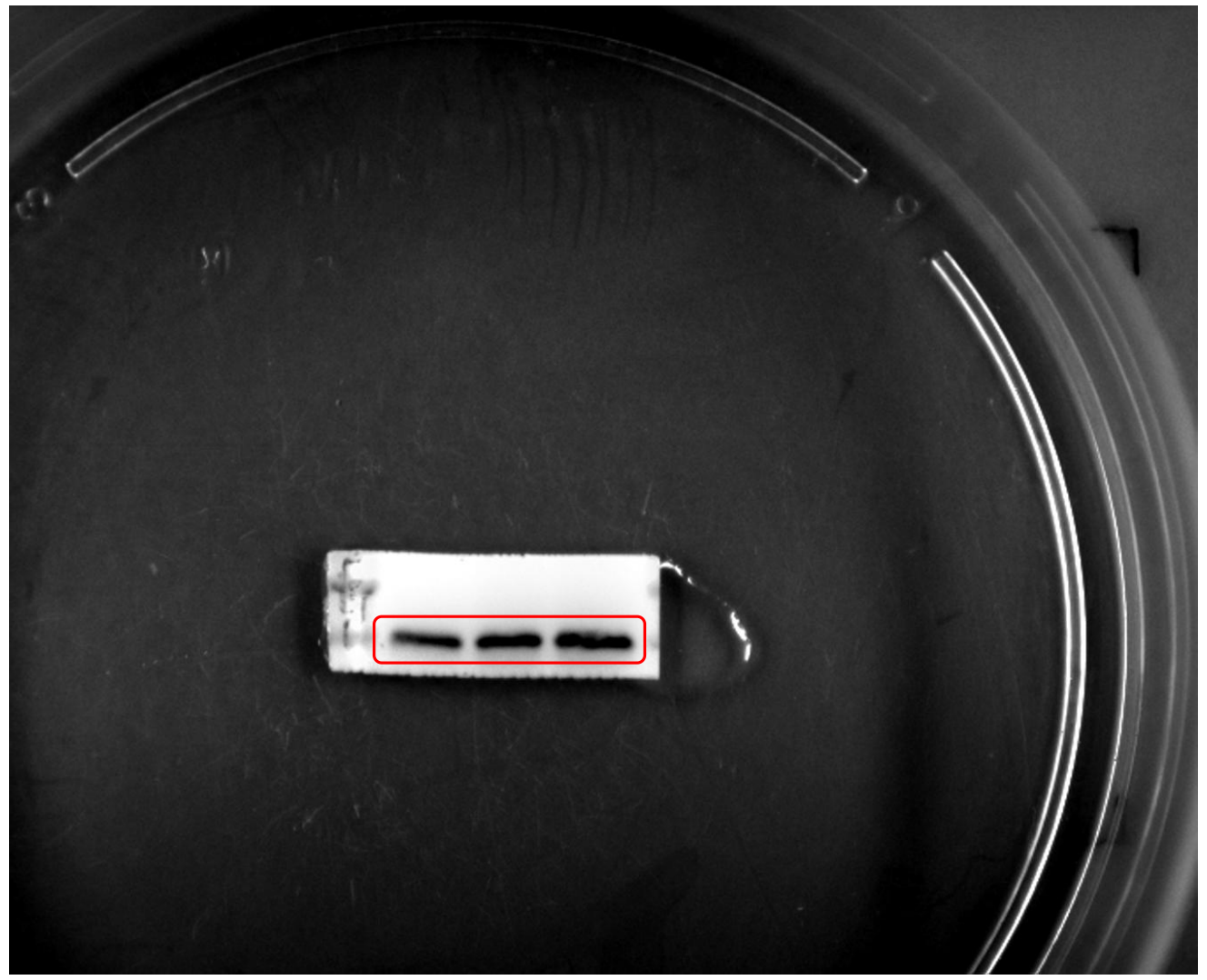

Figure 3E (from left to right: 0μM, 80μM,100μM)

**HONE1**

m-SREBP1

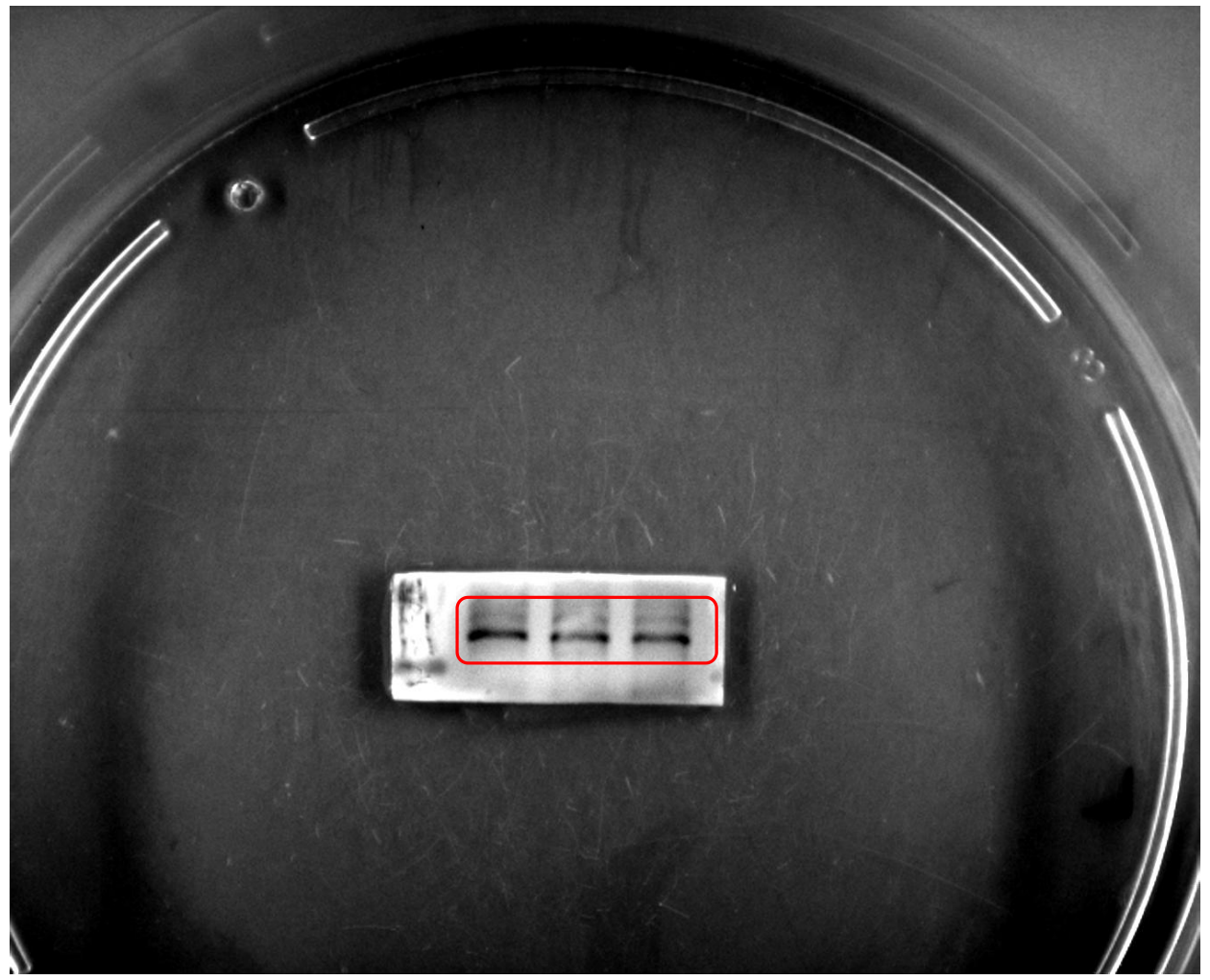

p-SREBP1

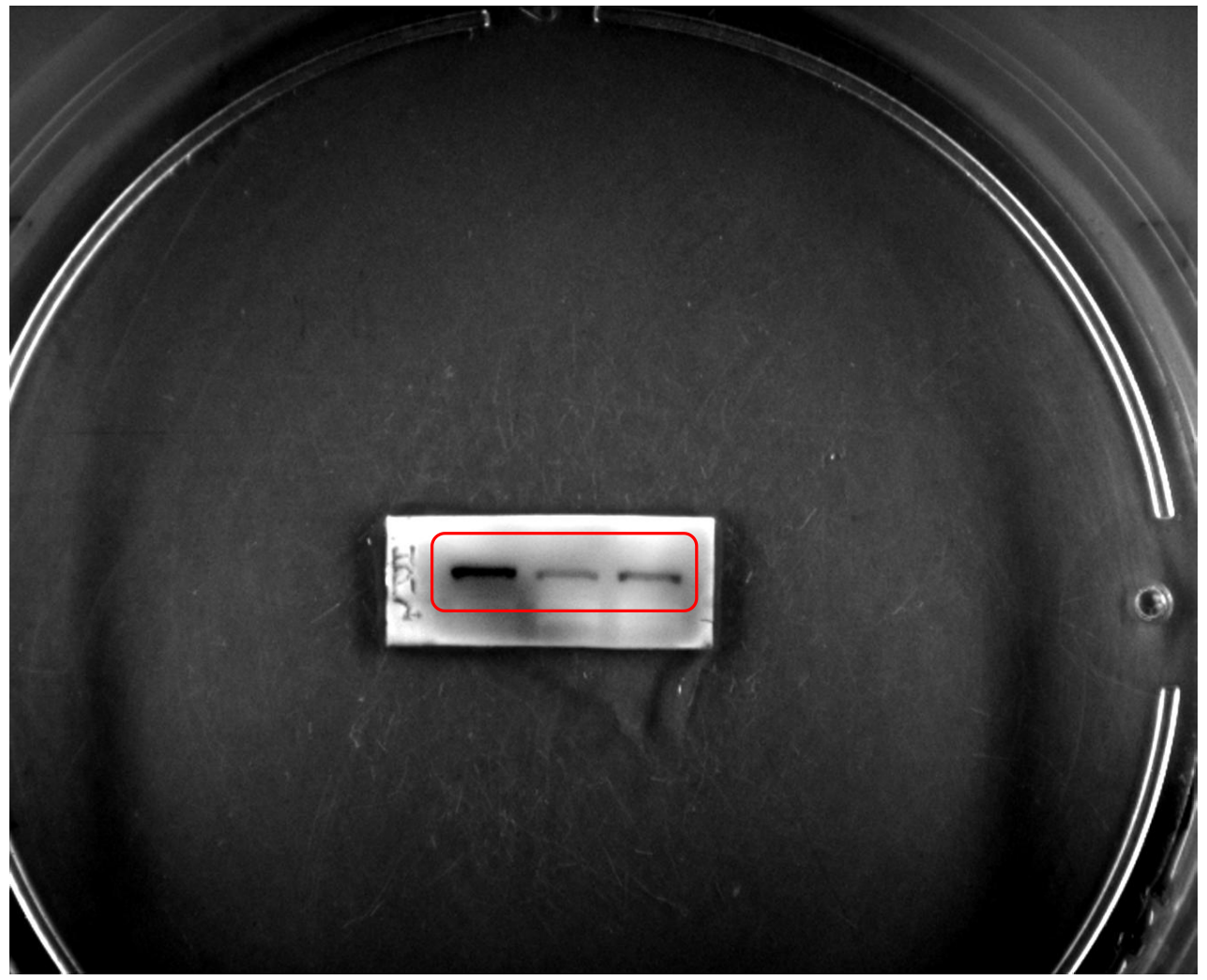

H3

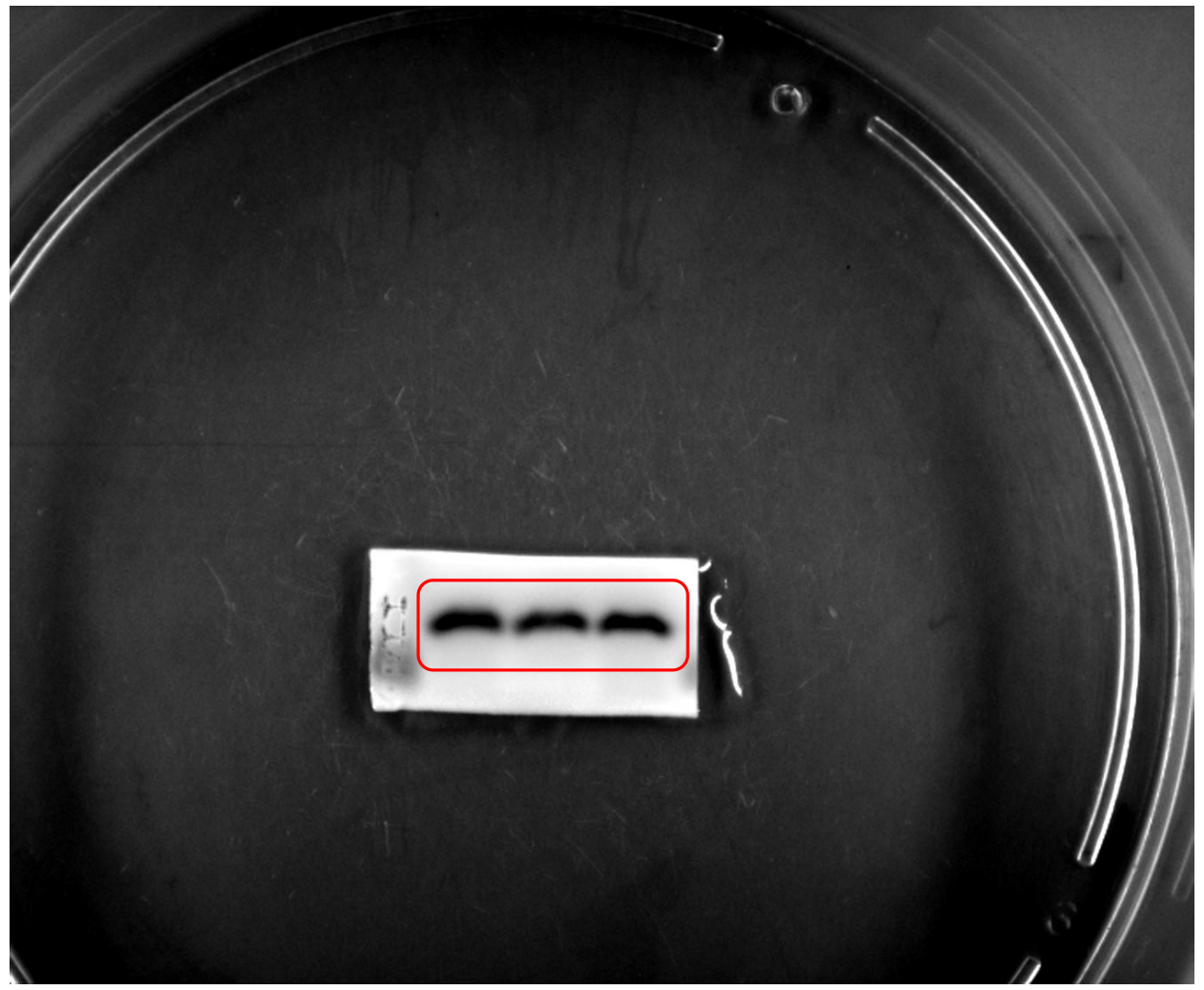

GAPDH

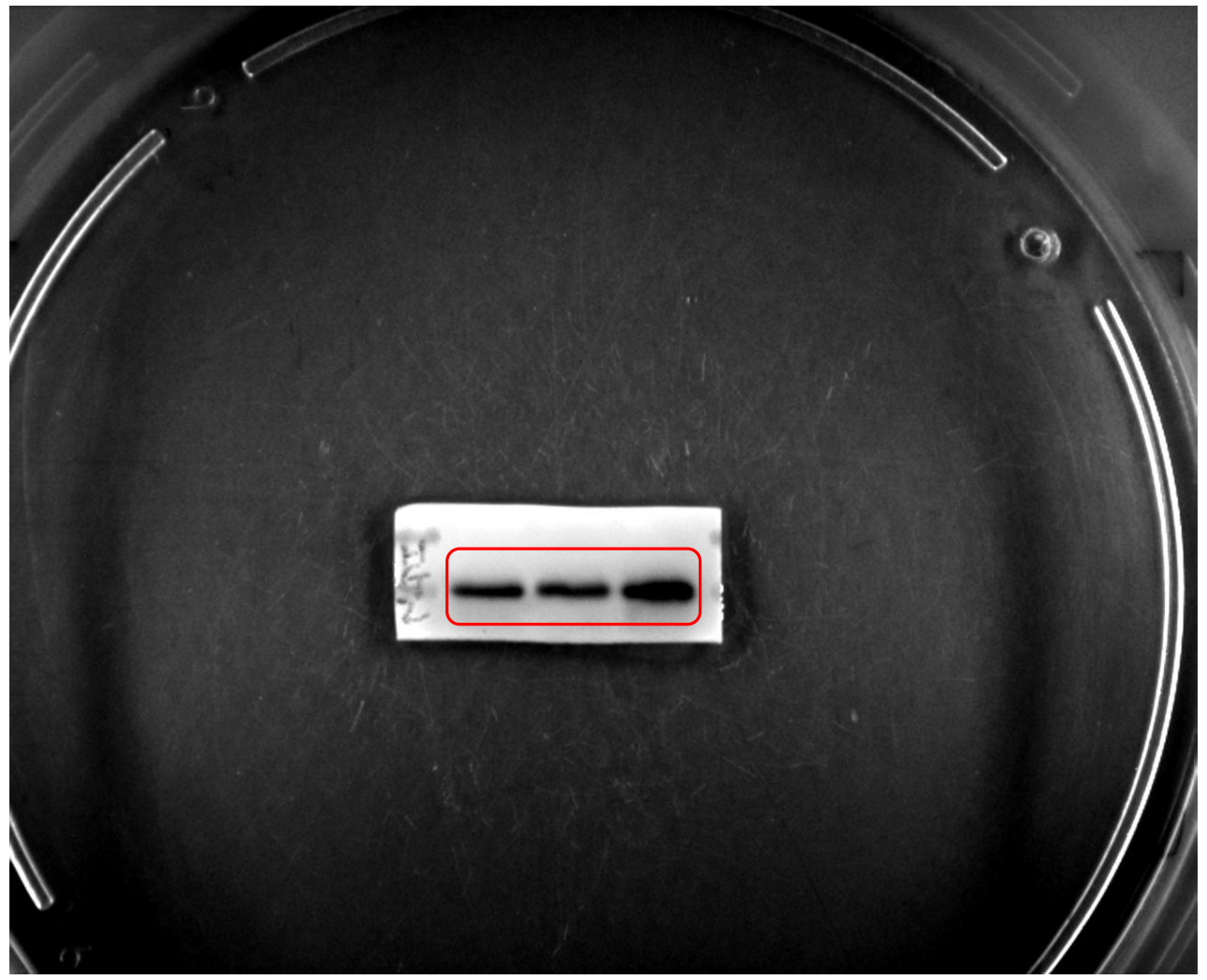

Figure 4A （from left to right:NC, si-p53-1, si-p53-2）

**5-8F**

SREBP1

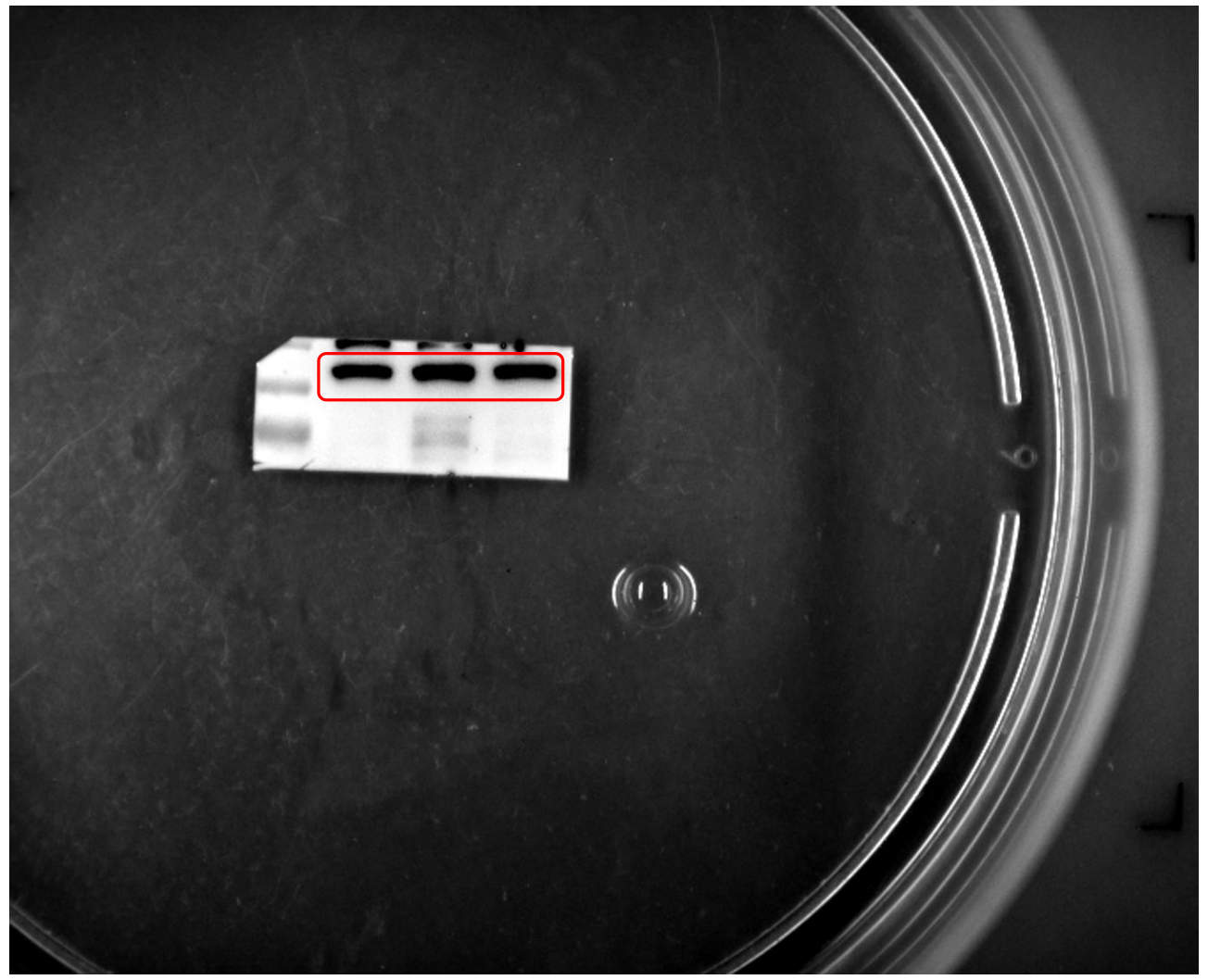

SREBP2

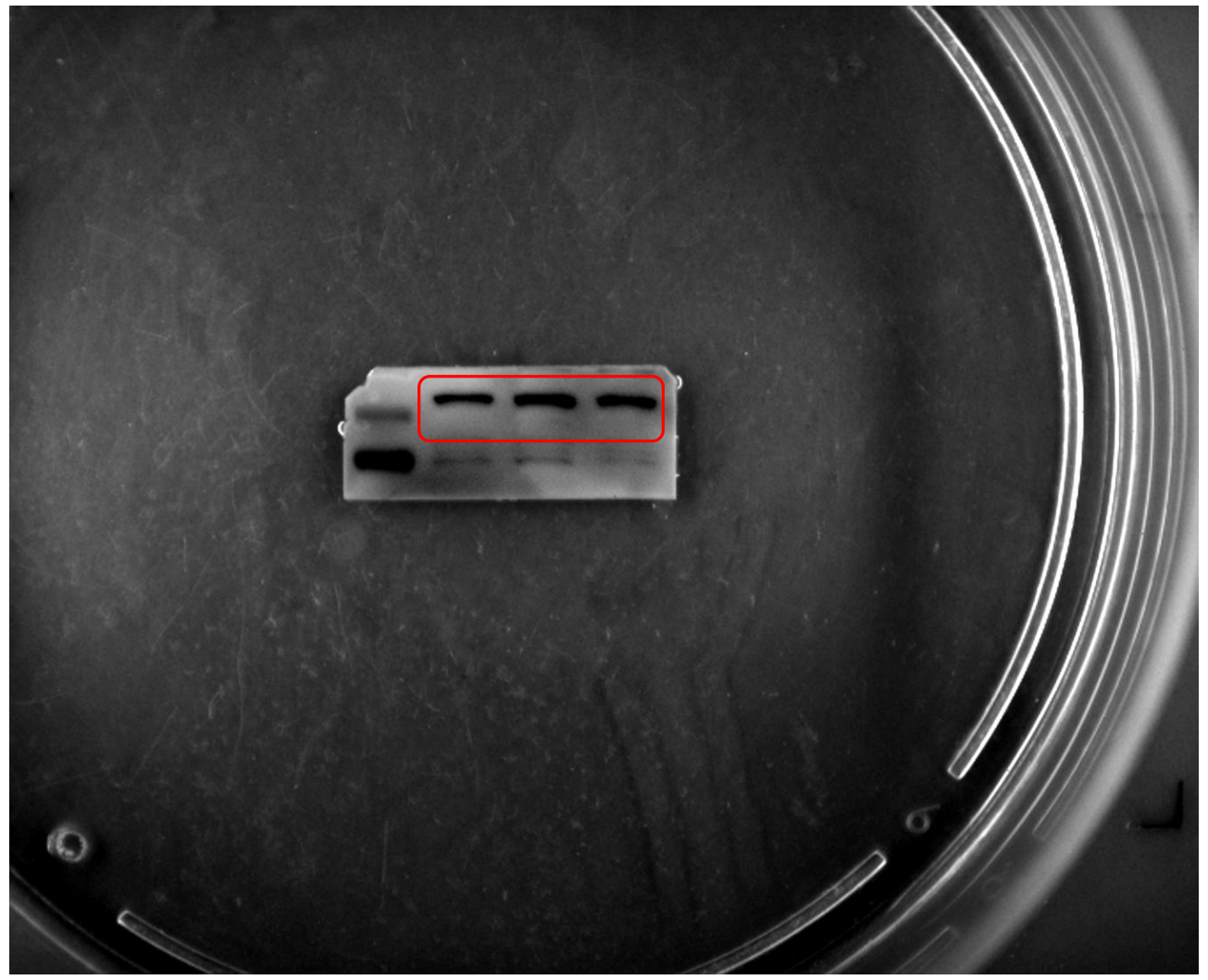

p53

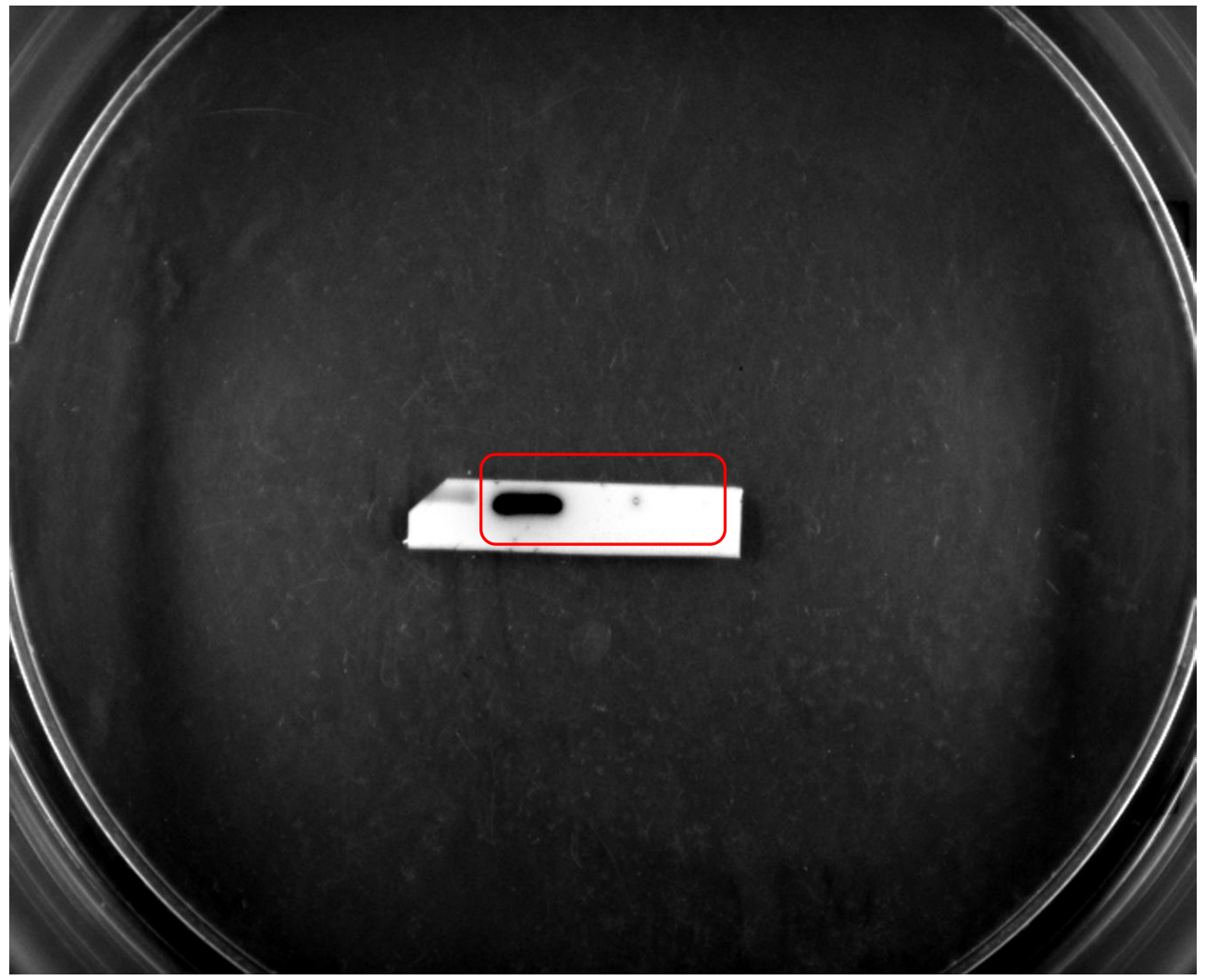

GAPDH

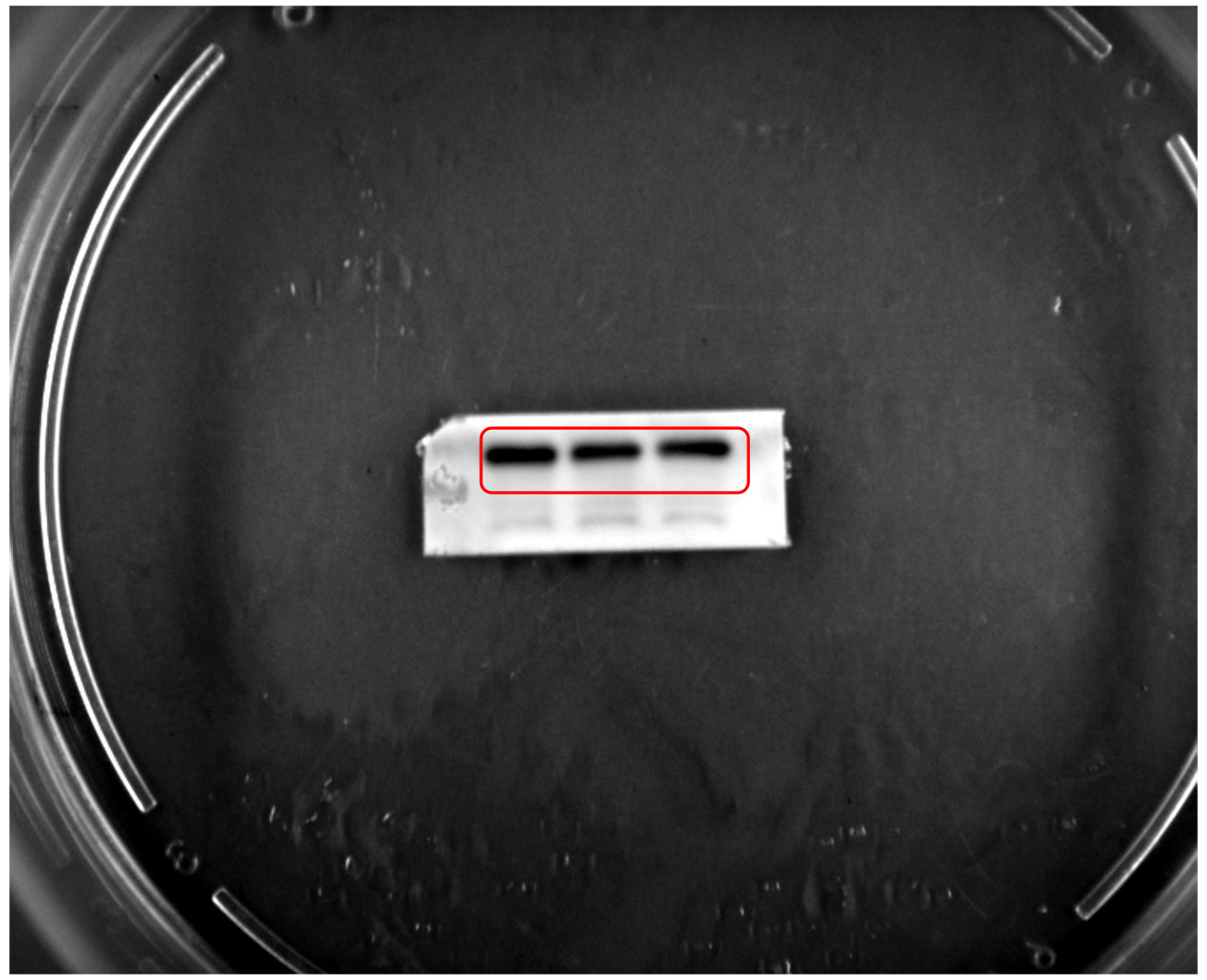

Figure 4A （from left to right: NC, si-p53-1, si-p53-2）

**HONE1**

SREBP1

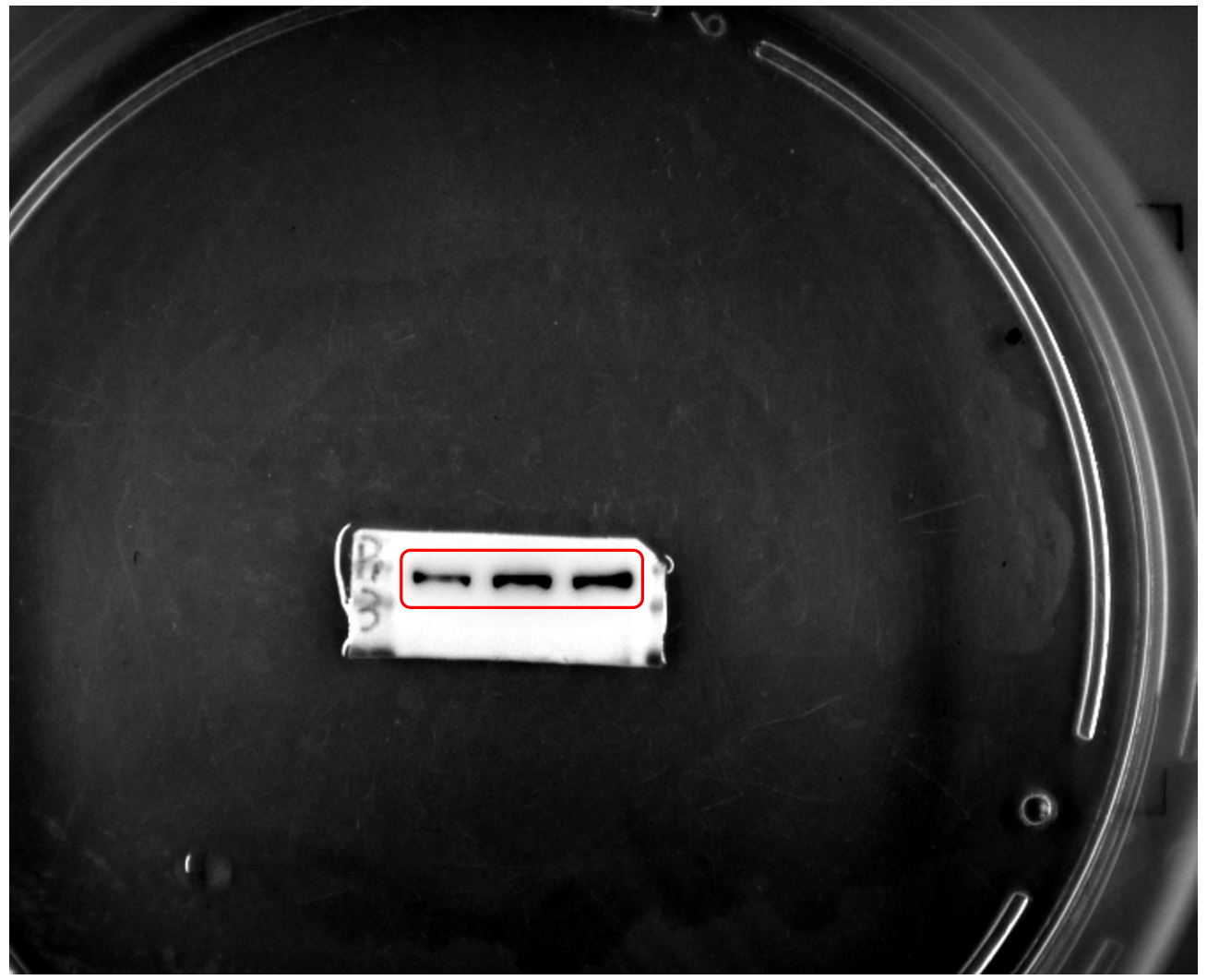

SREBP2

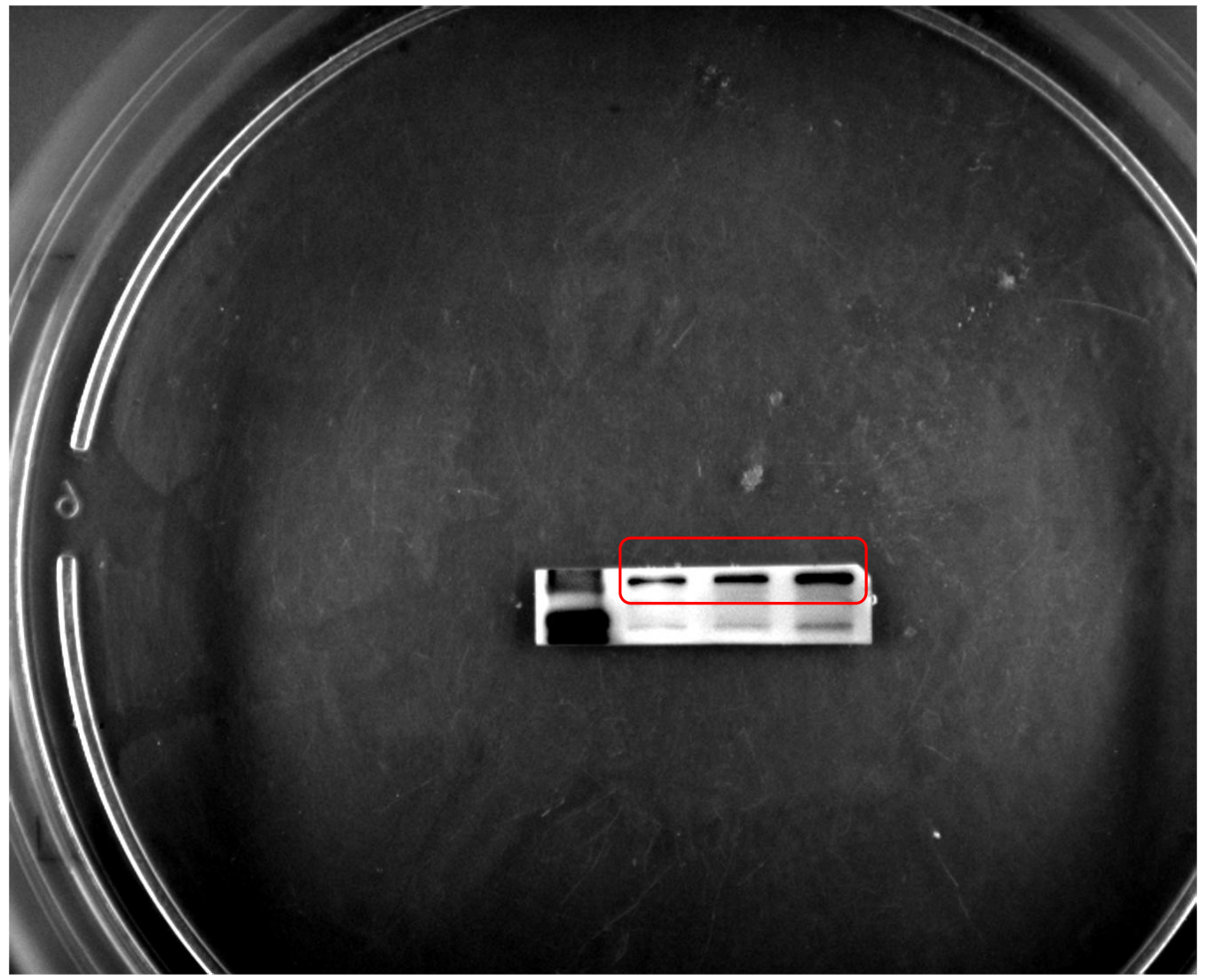

p53

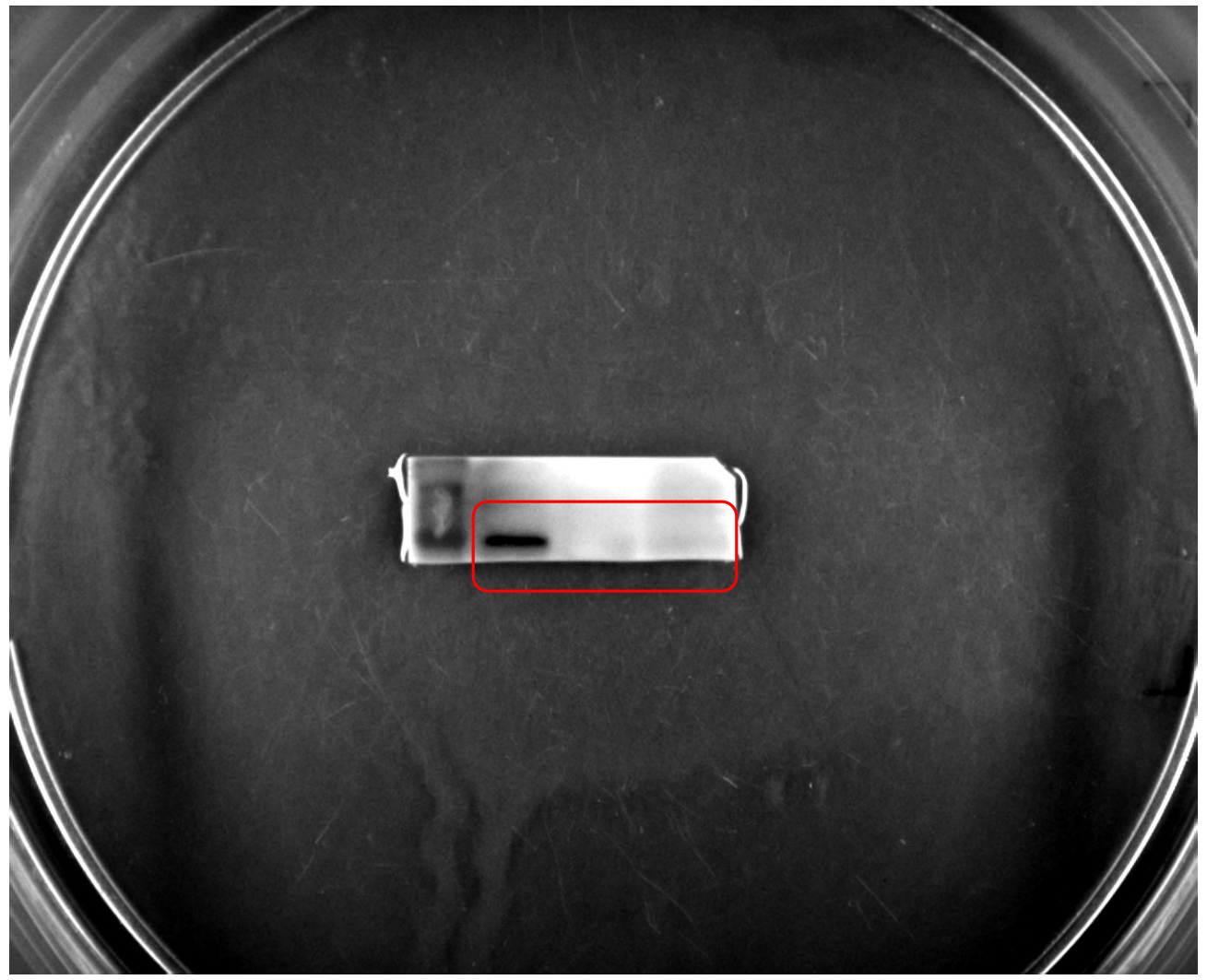

GAPDH

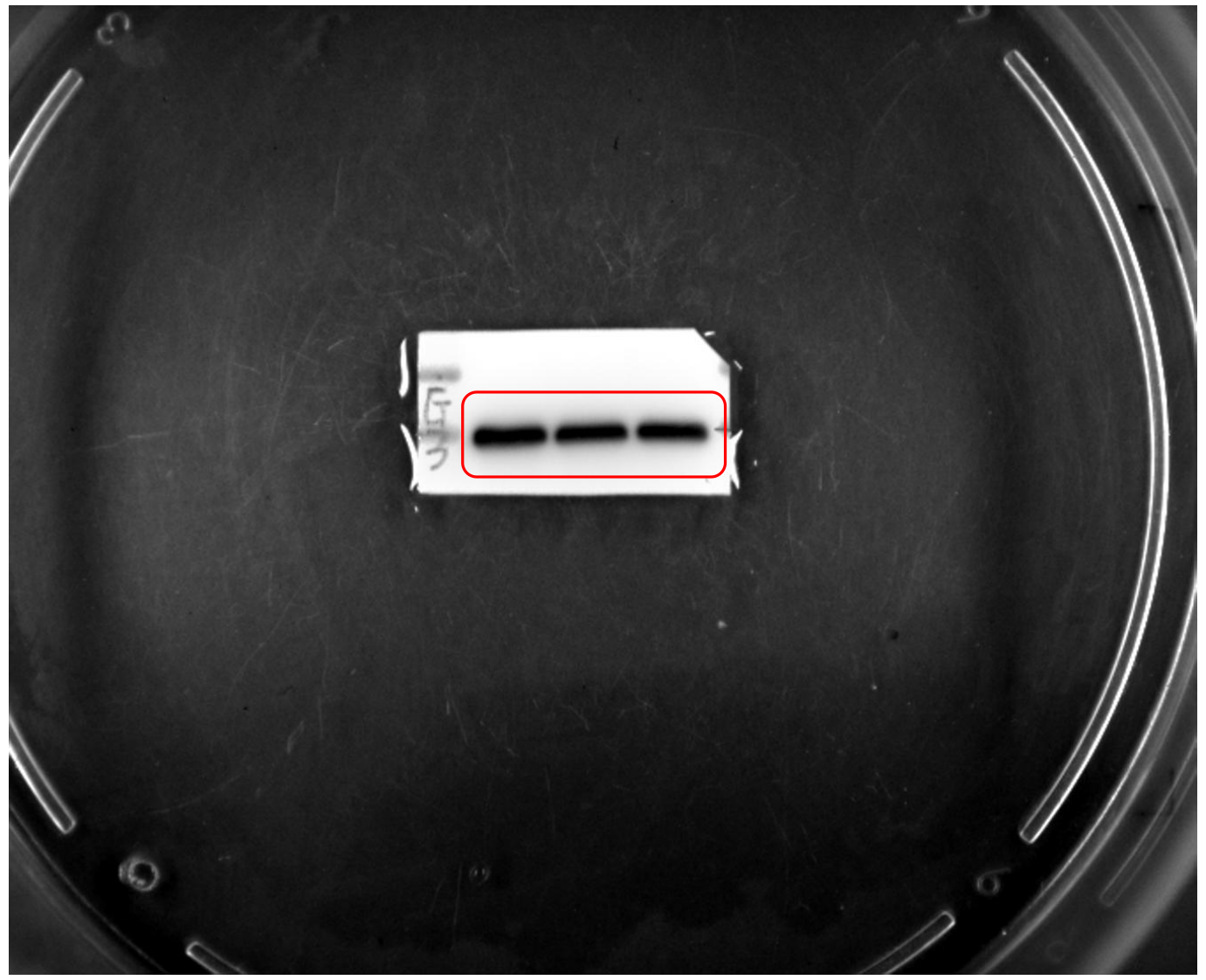

Figure 4B（from left to right: NC, 5-MF+NC, 5-MF+si-p53-1, 5-MF+si-p53-2）

**5-8F**

SREBP1

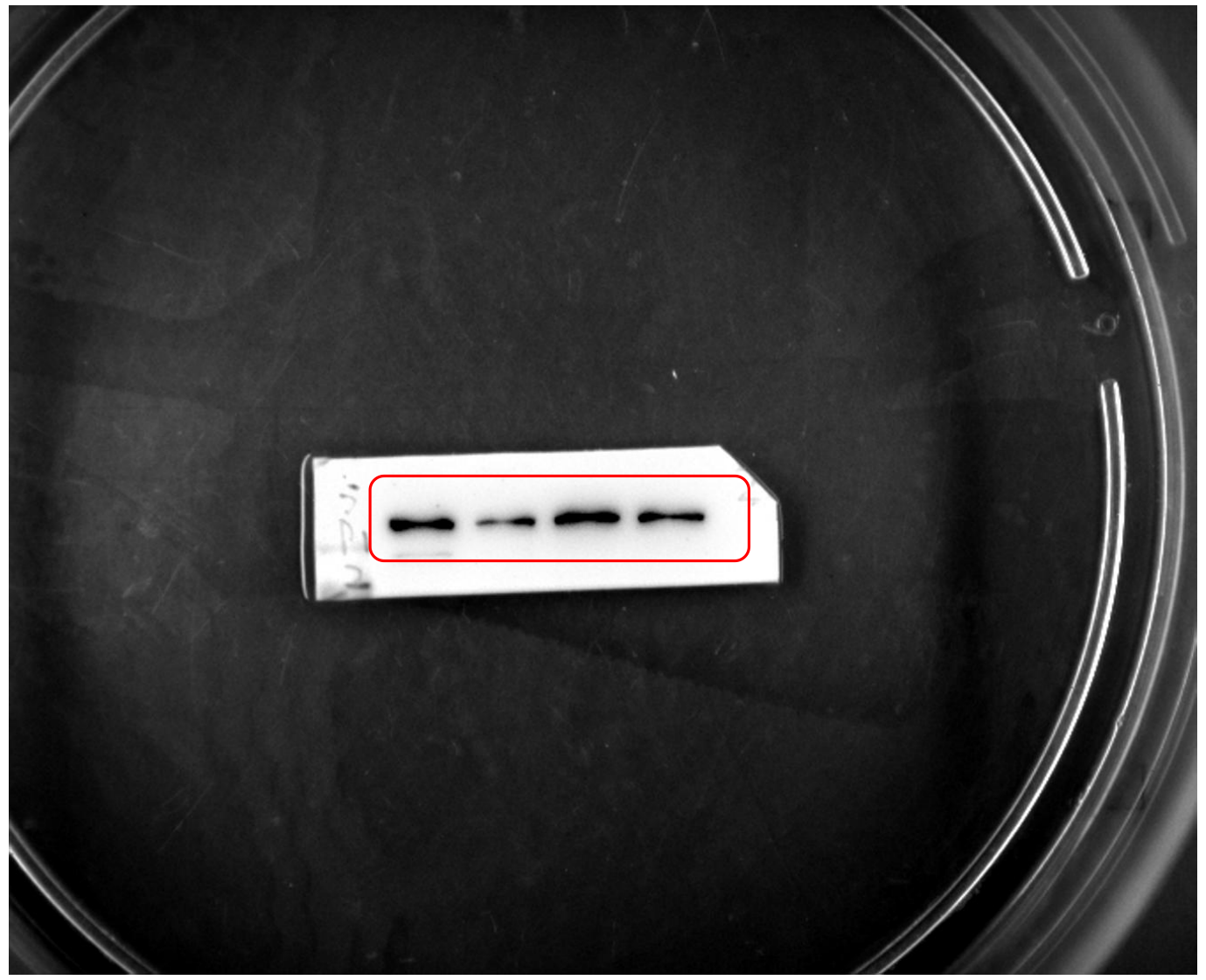

SREBP2

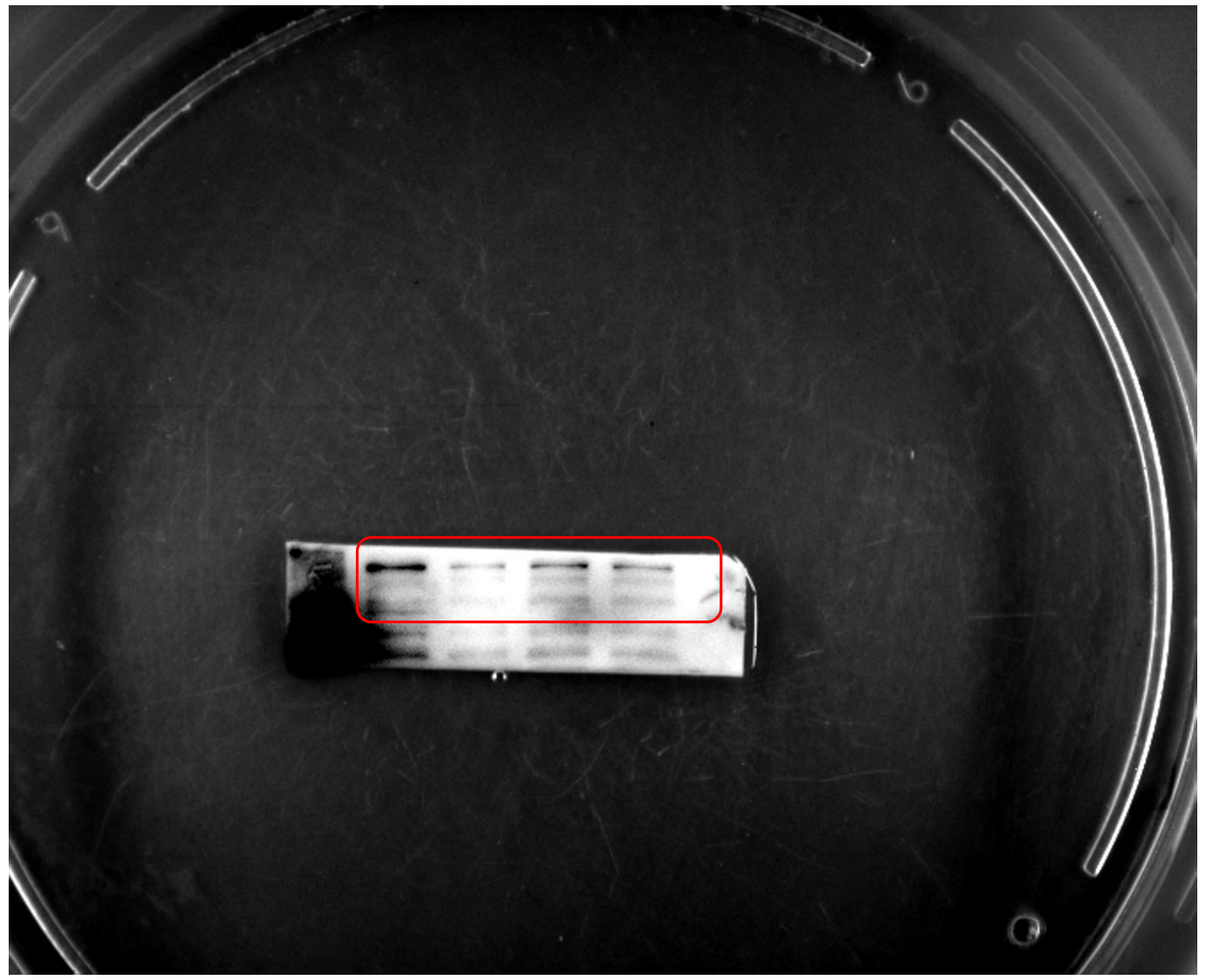

p53

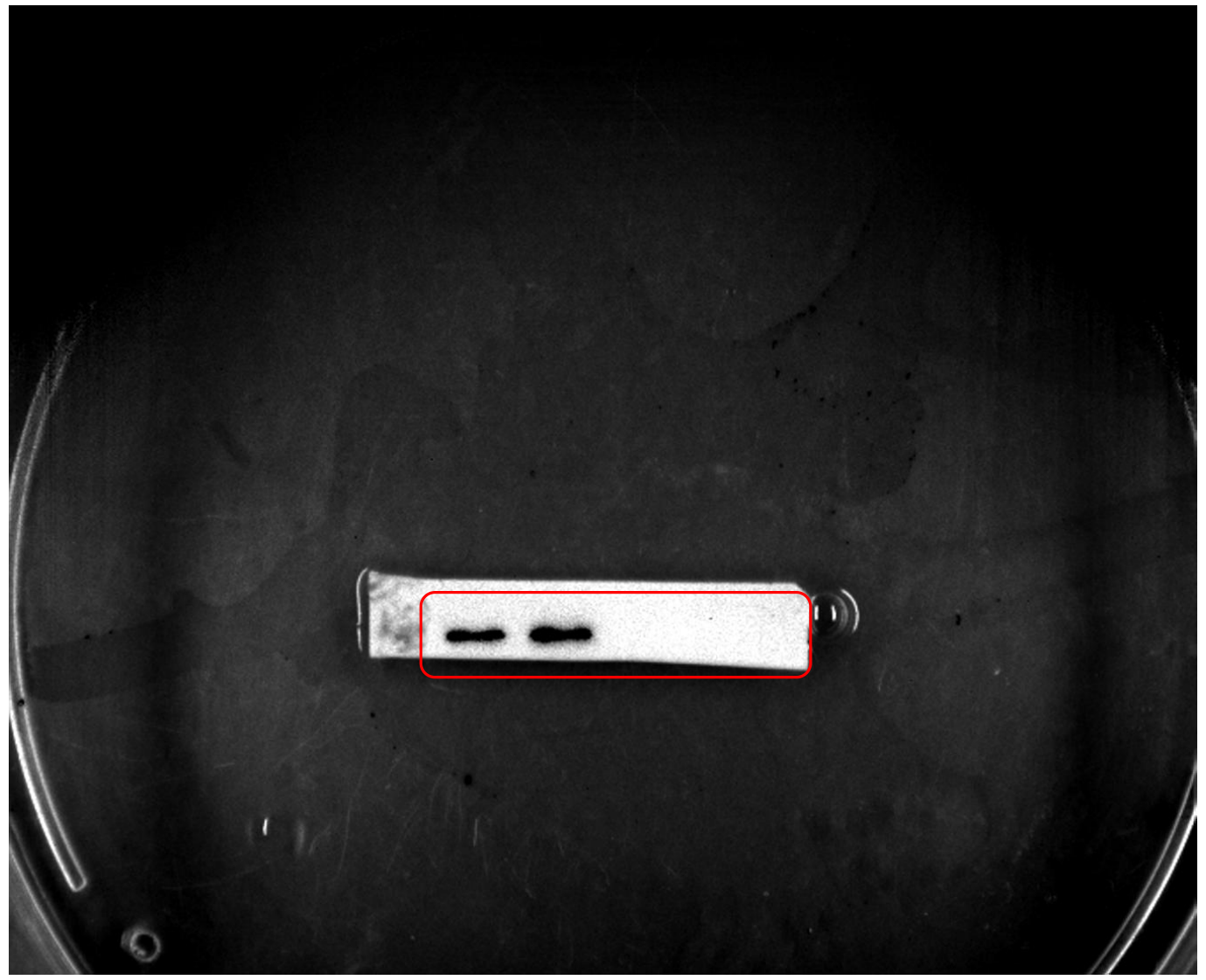

GAPDH

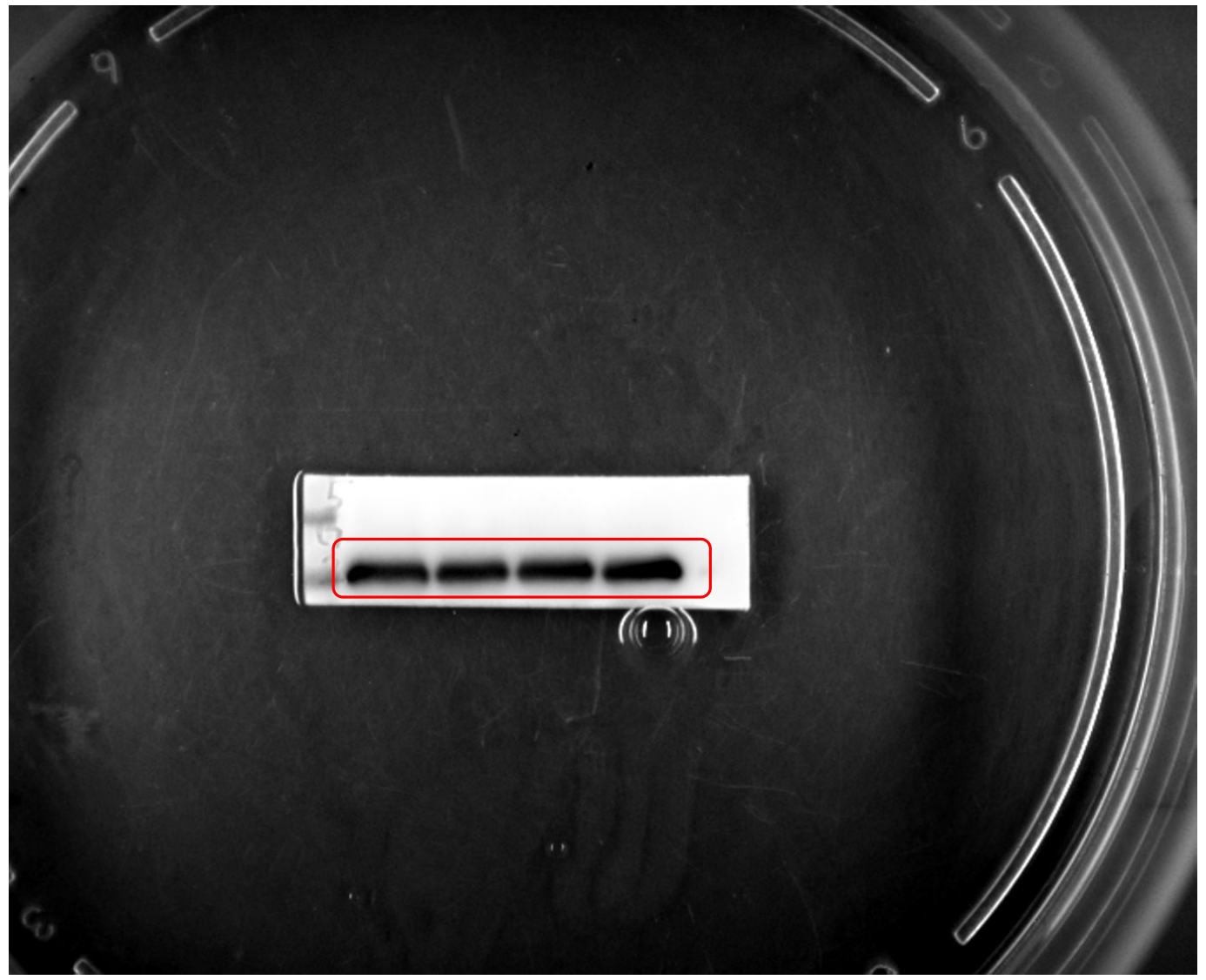

Figure 4B（from left to right: NC, 5-MF+NC, 5-MF+si-p53-1, 5-MF+si-p53-2）

**HONE1**

SREBP1

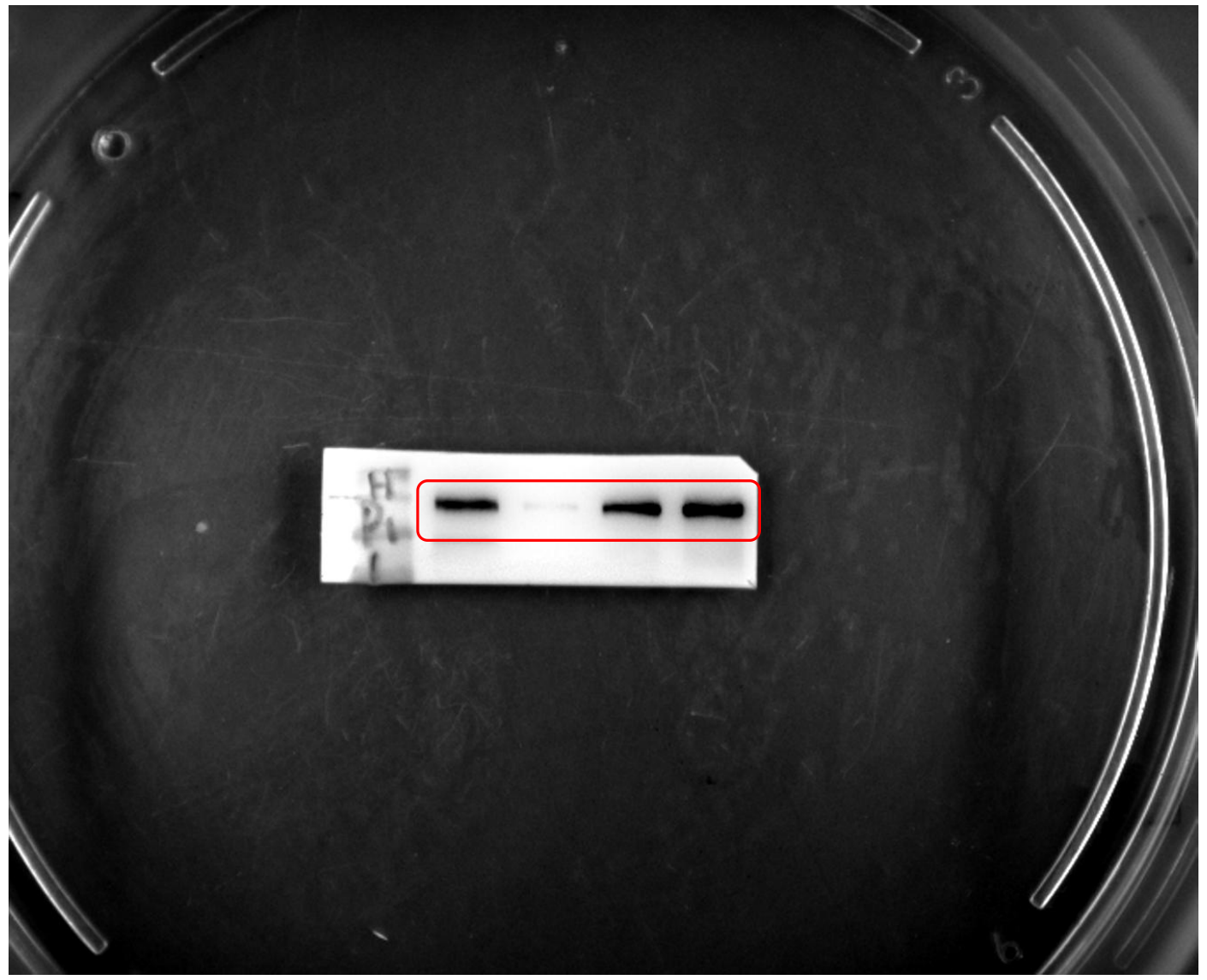

SREBP2

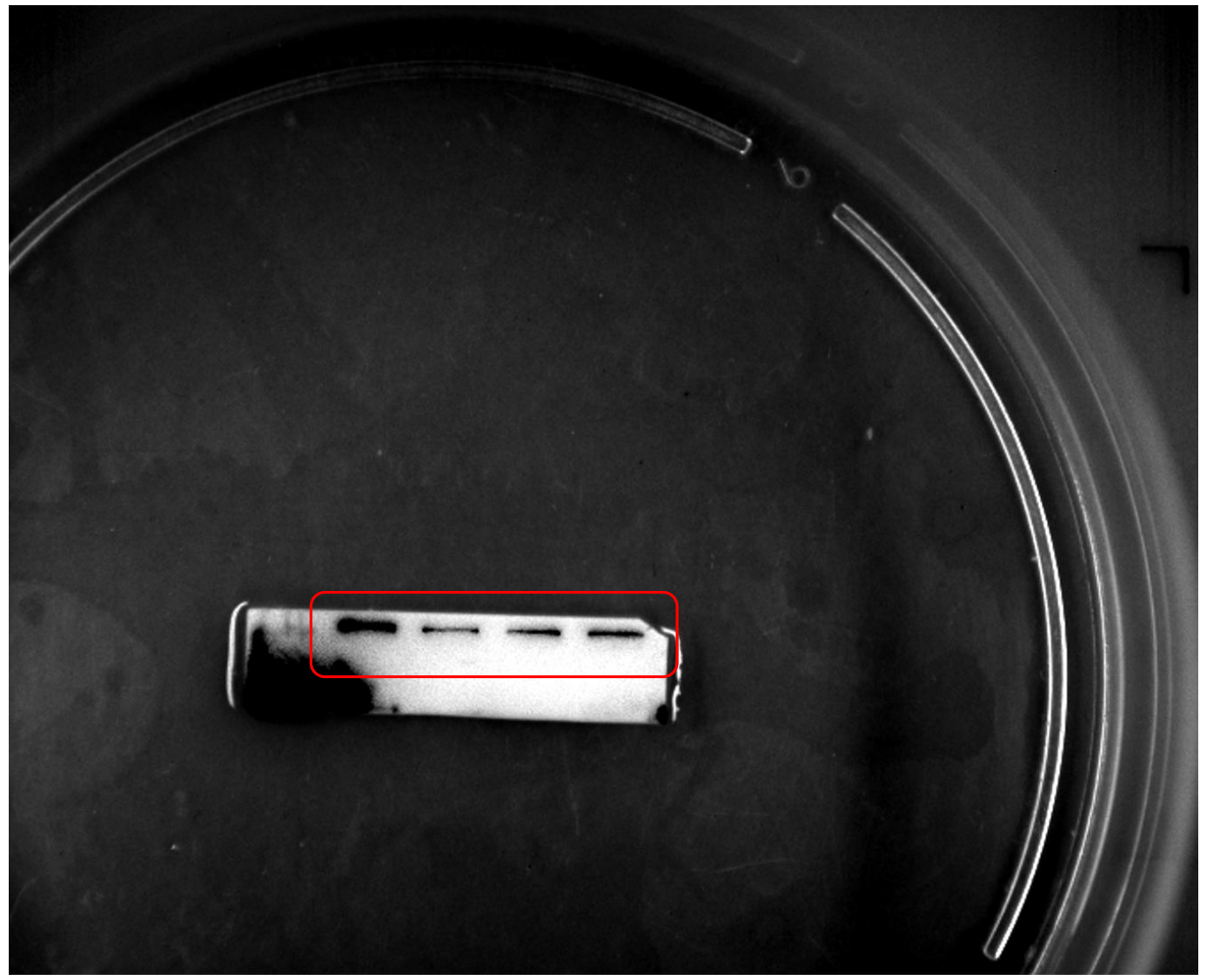

p53

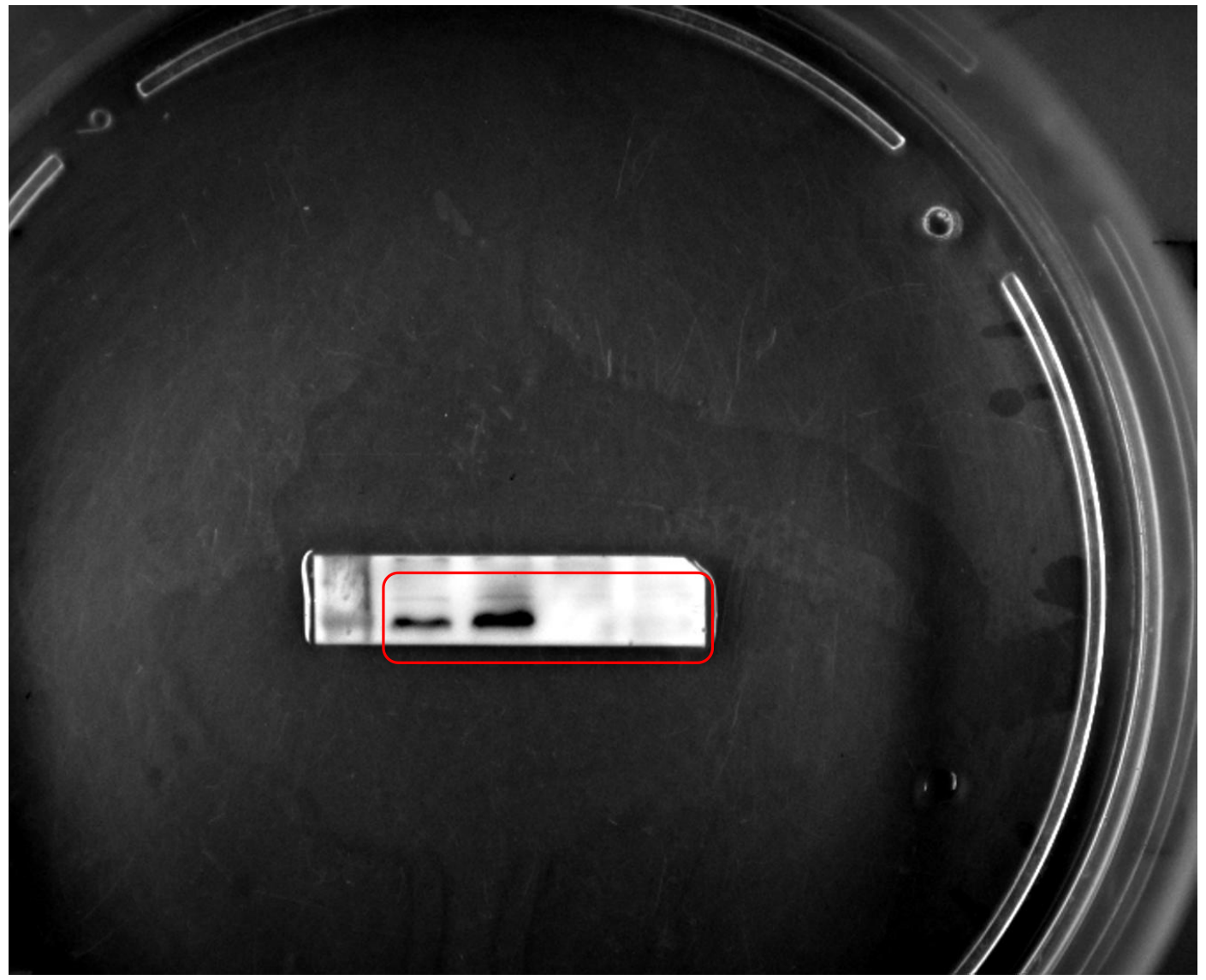

GAPDH

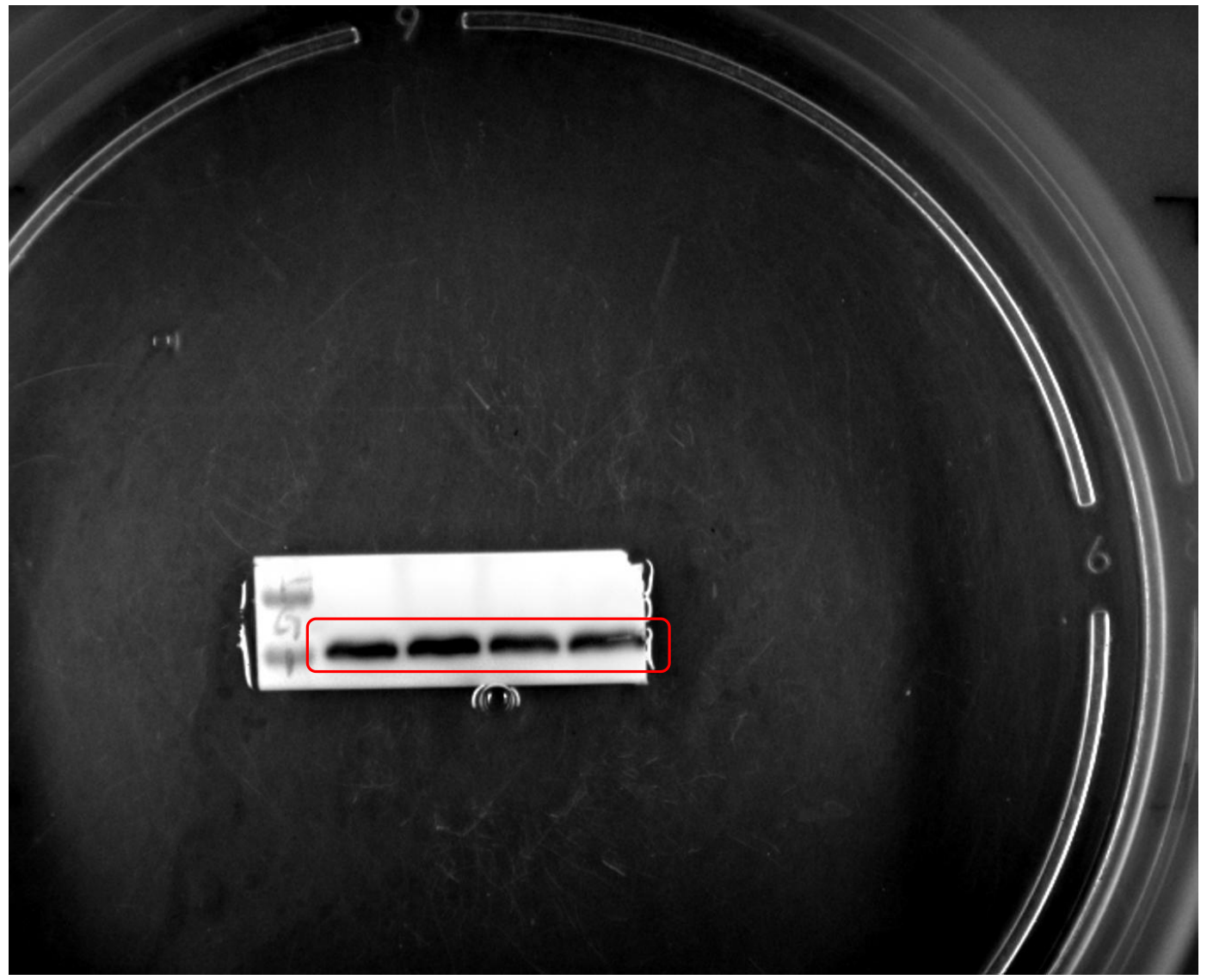

Figure 4C（from left to right: NC, 5-MF+NC, 5-MF+si-p53-1, 5-MF+si-p53-2）

**5-8F**

m-SREBP1

p-SREBP1

H3

GAPDH

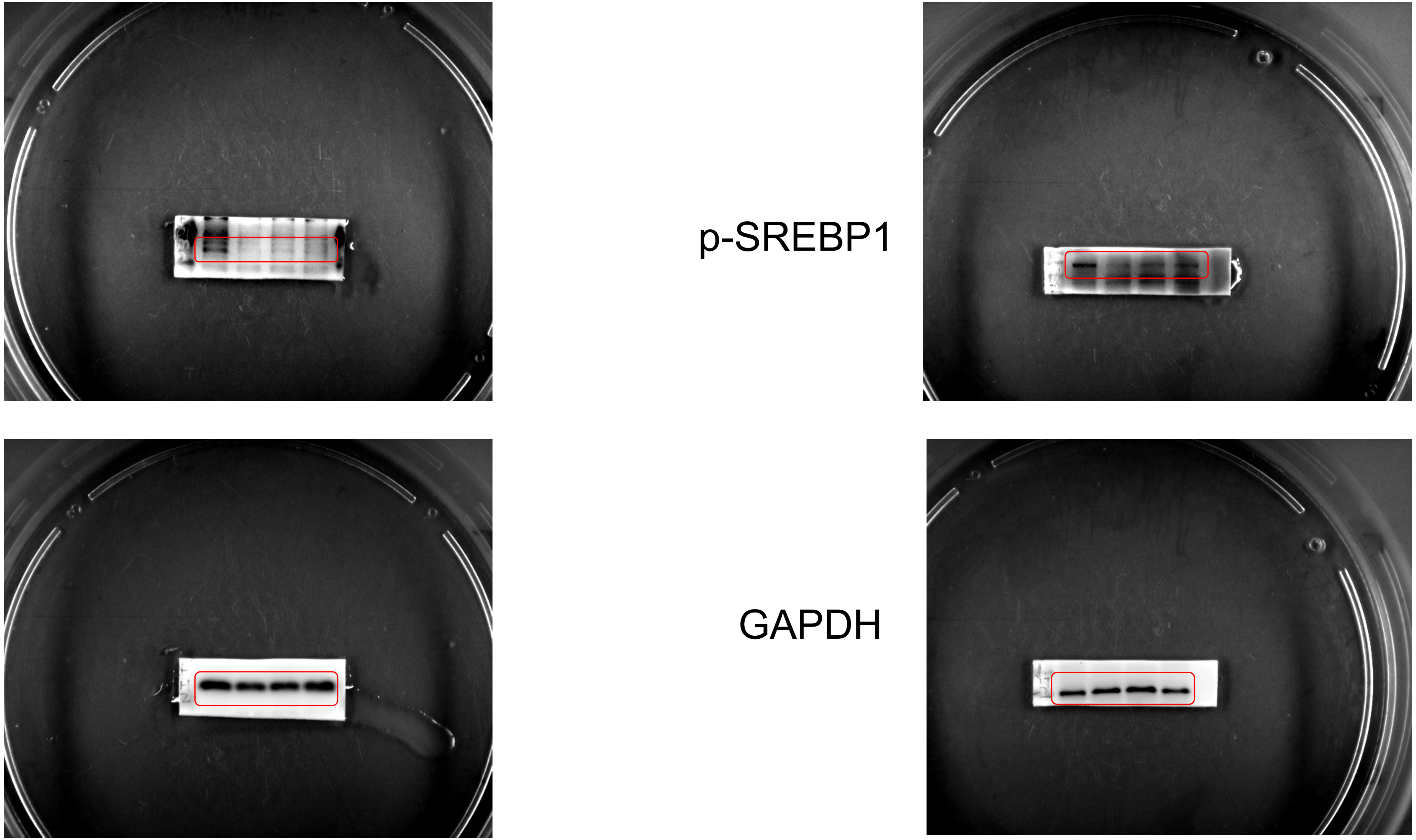

Figure 4C（from left to right: NC, 5-MF+NC, 5-MF+si-p53-1, 5-MF+si-p53-2）

**HONE1**

m-SREBP1

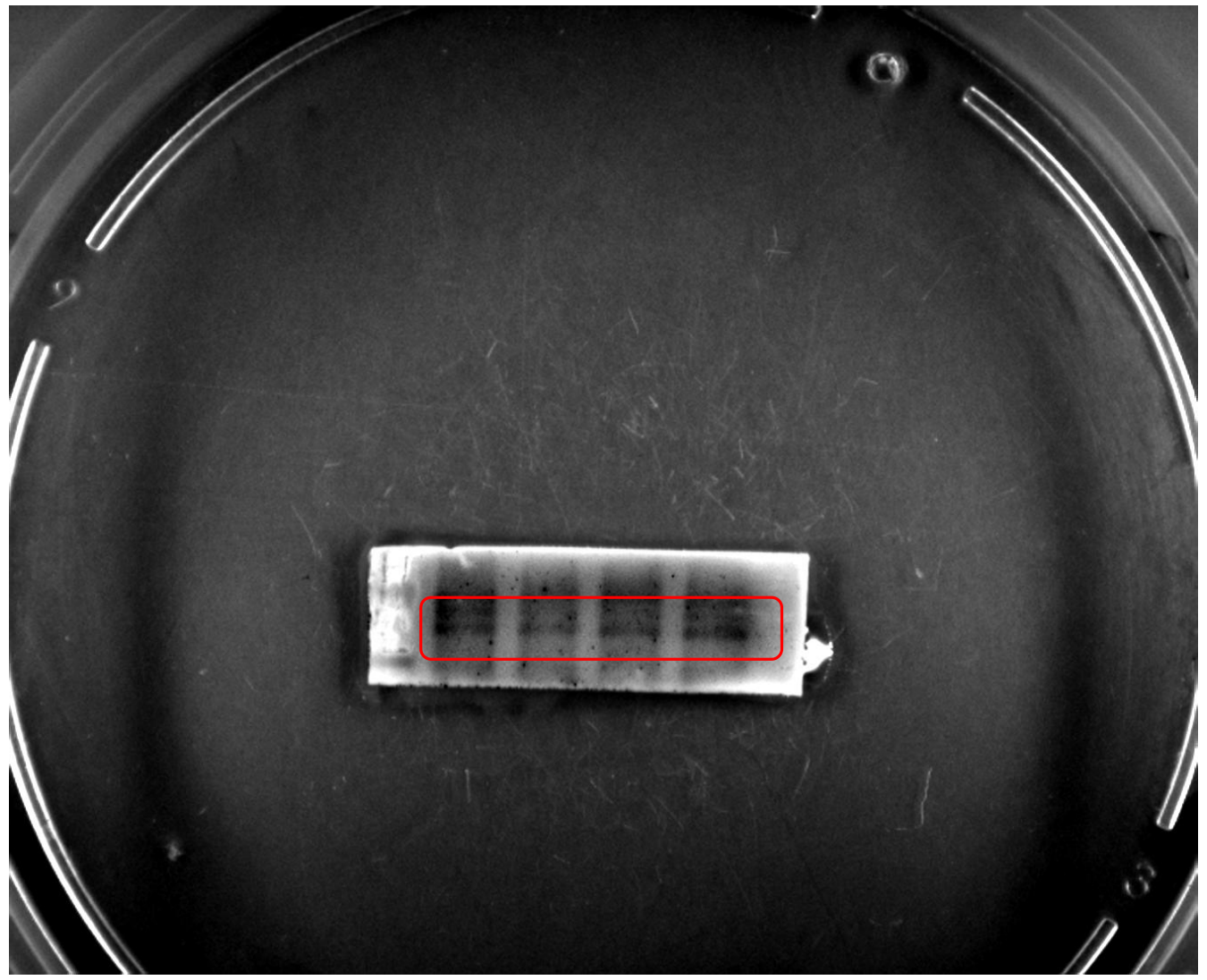

p-SREBP1

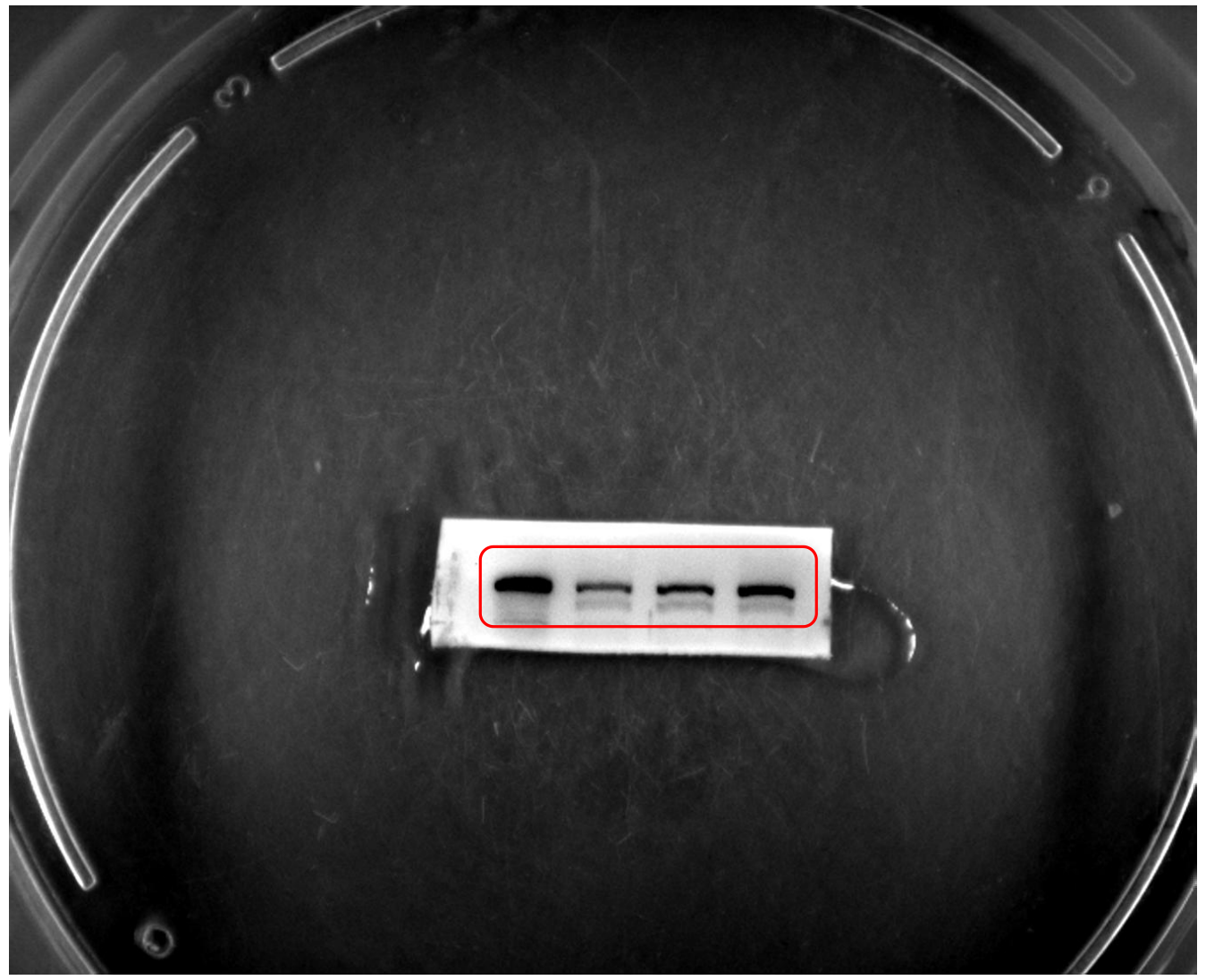

H3

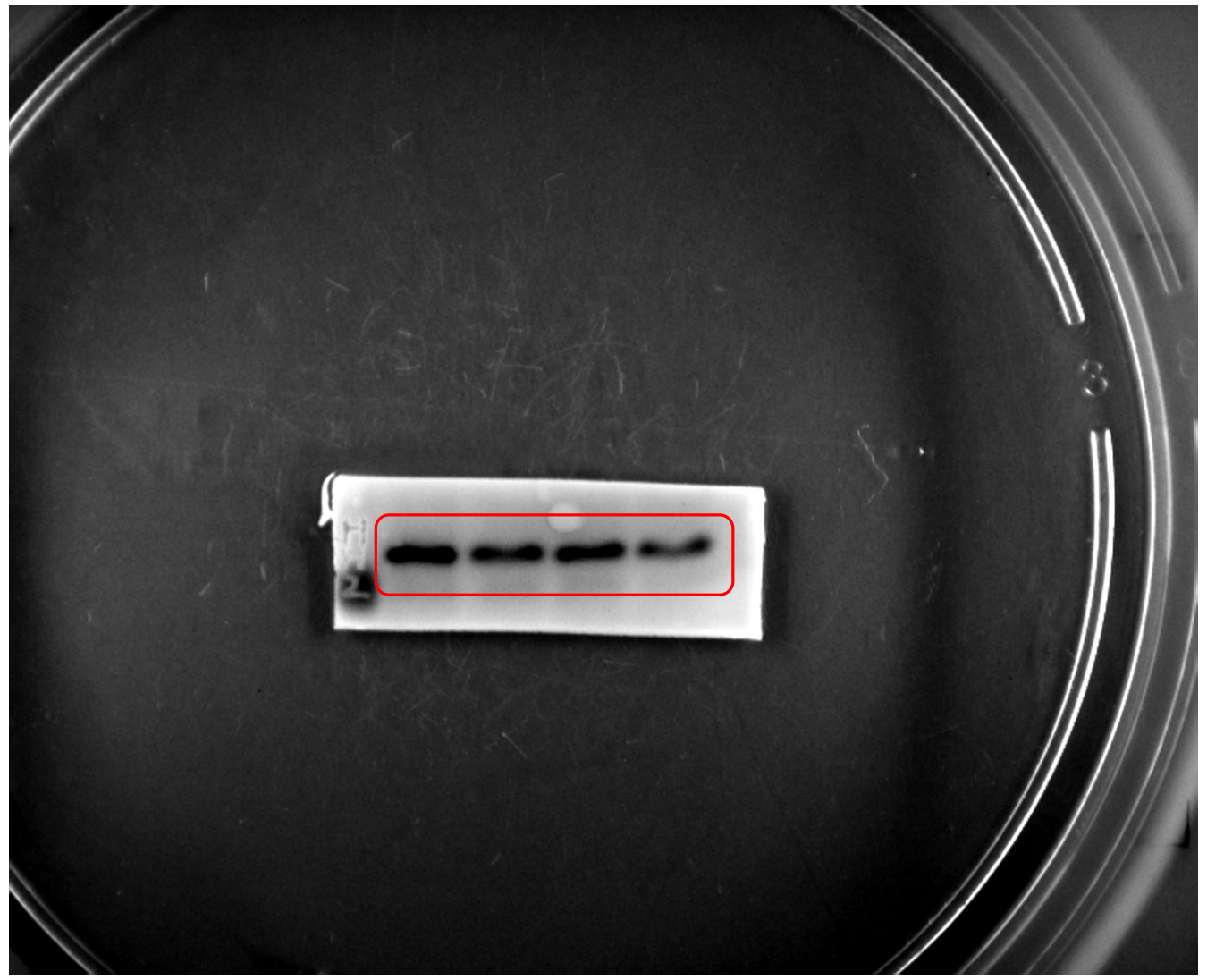

GAPDH

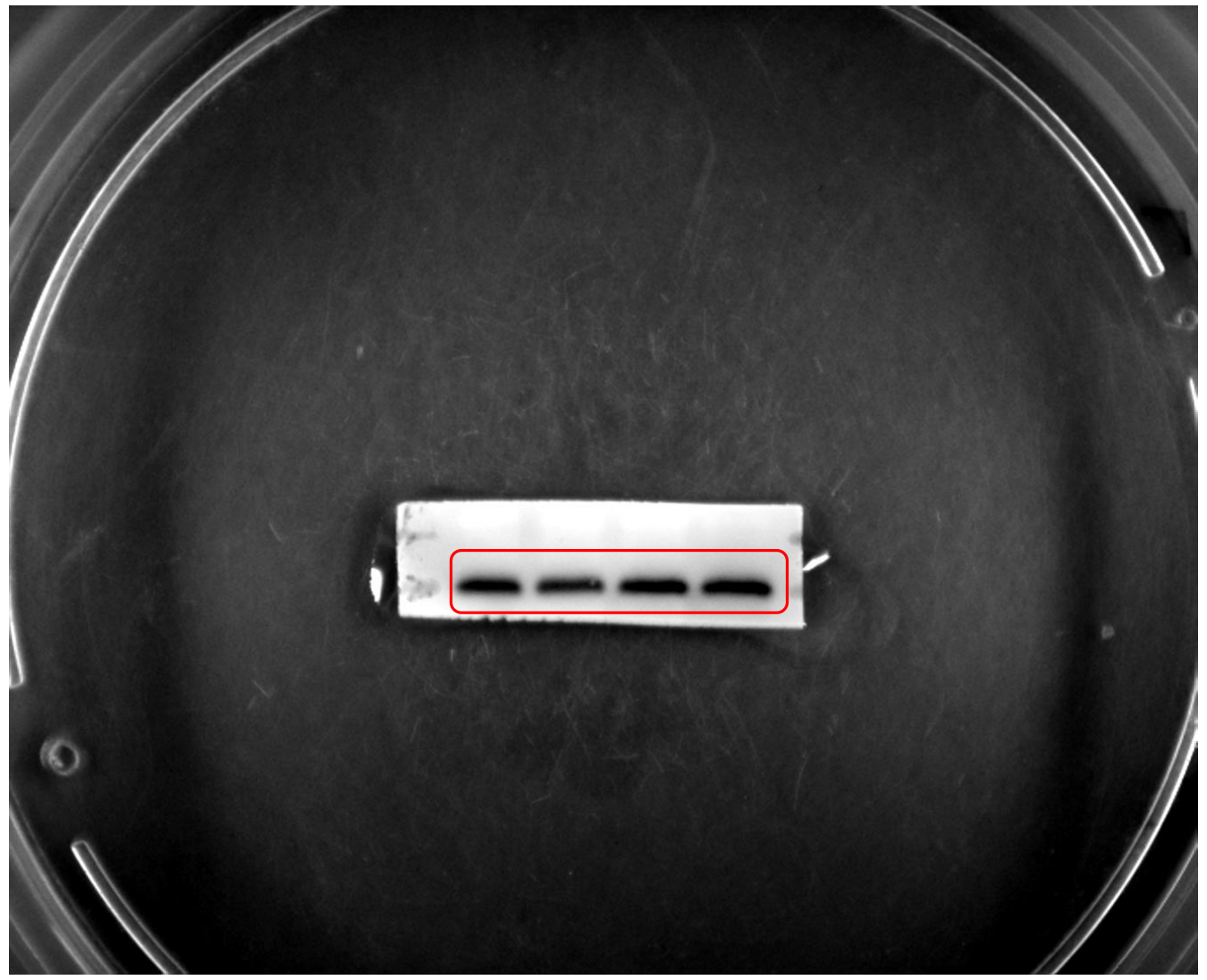

Figure 5E（from left to right: 0μM, 80μM,100μM）

**5-8F**

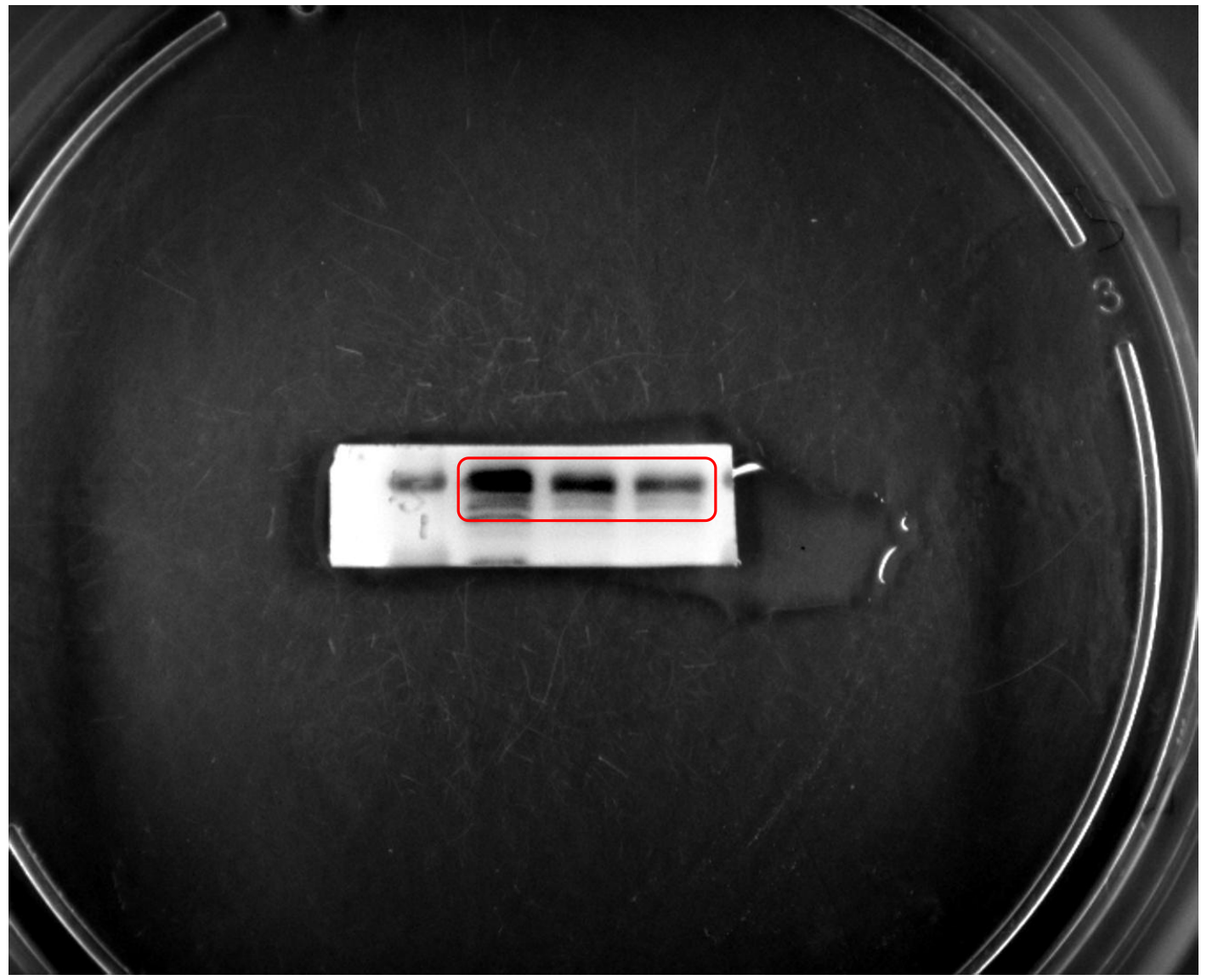

SLC7A11

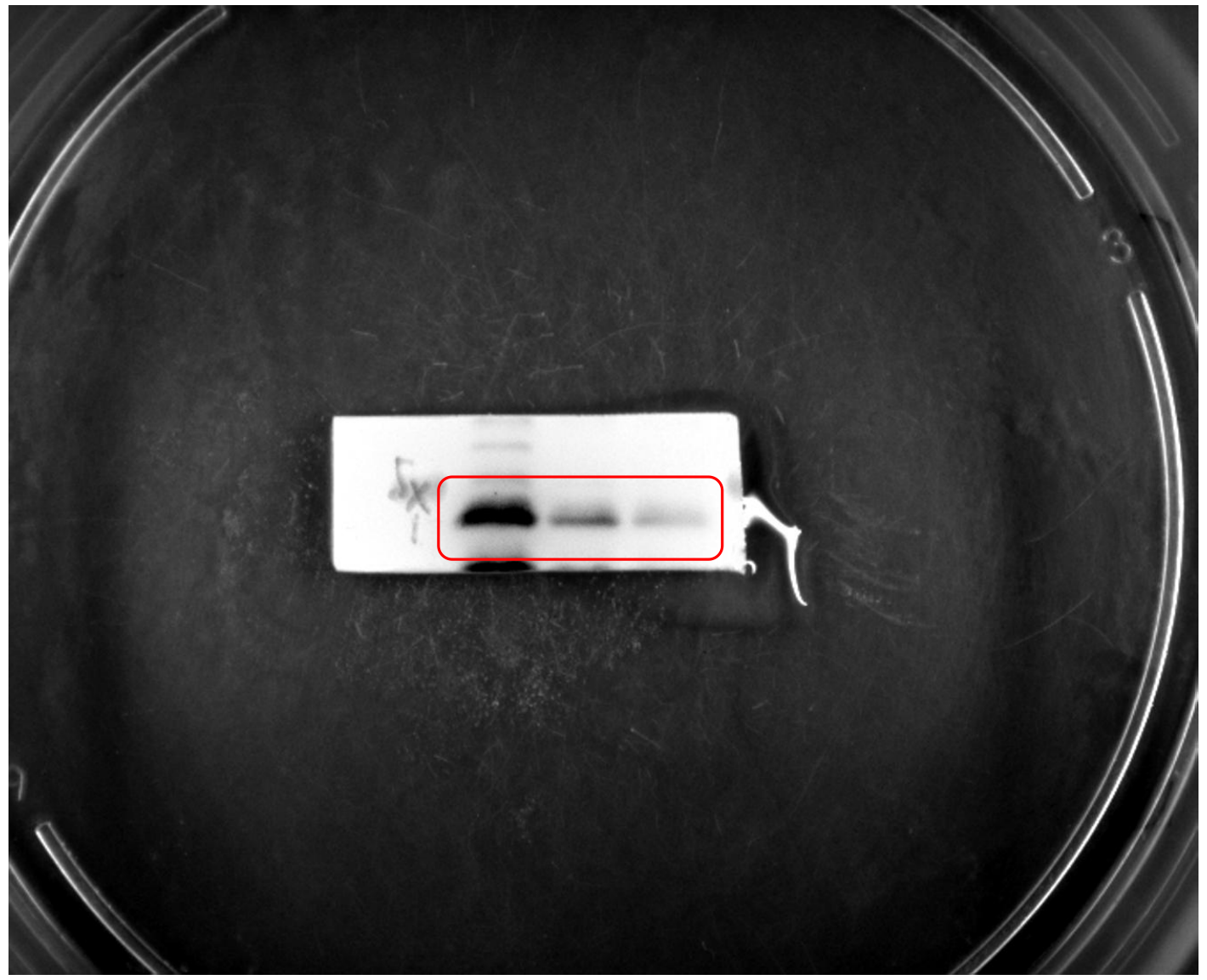

GPX4

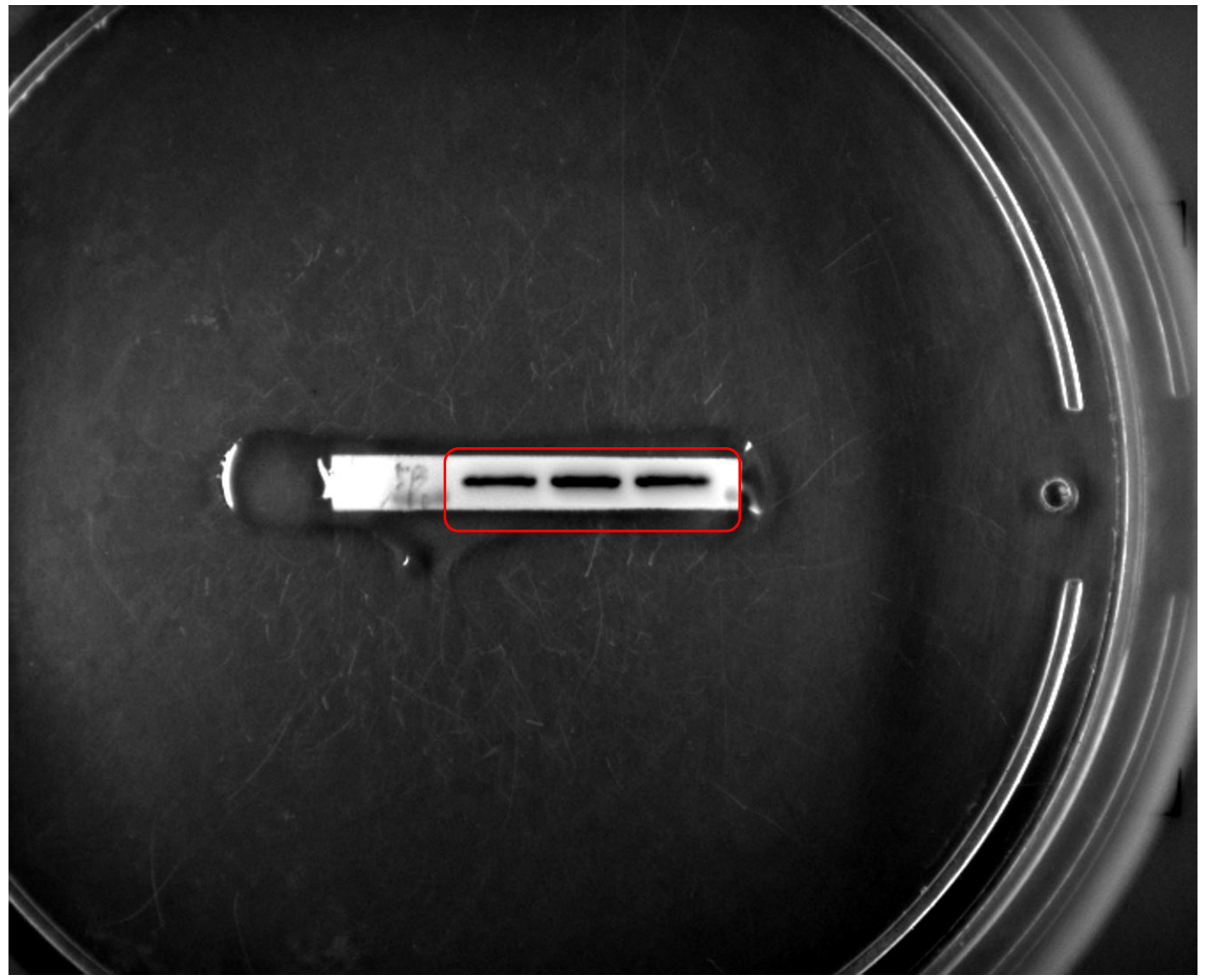

β-actin

Figure 5E（from left to right: 0μM, 80μM,100μM）

**5-8F**

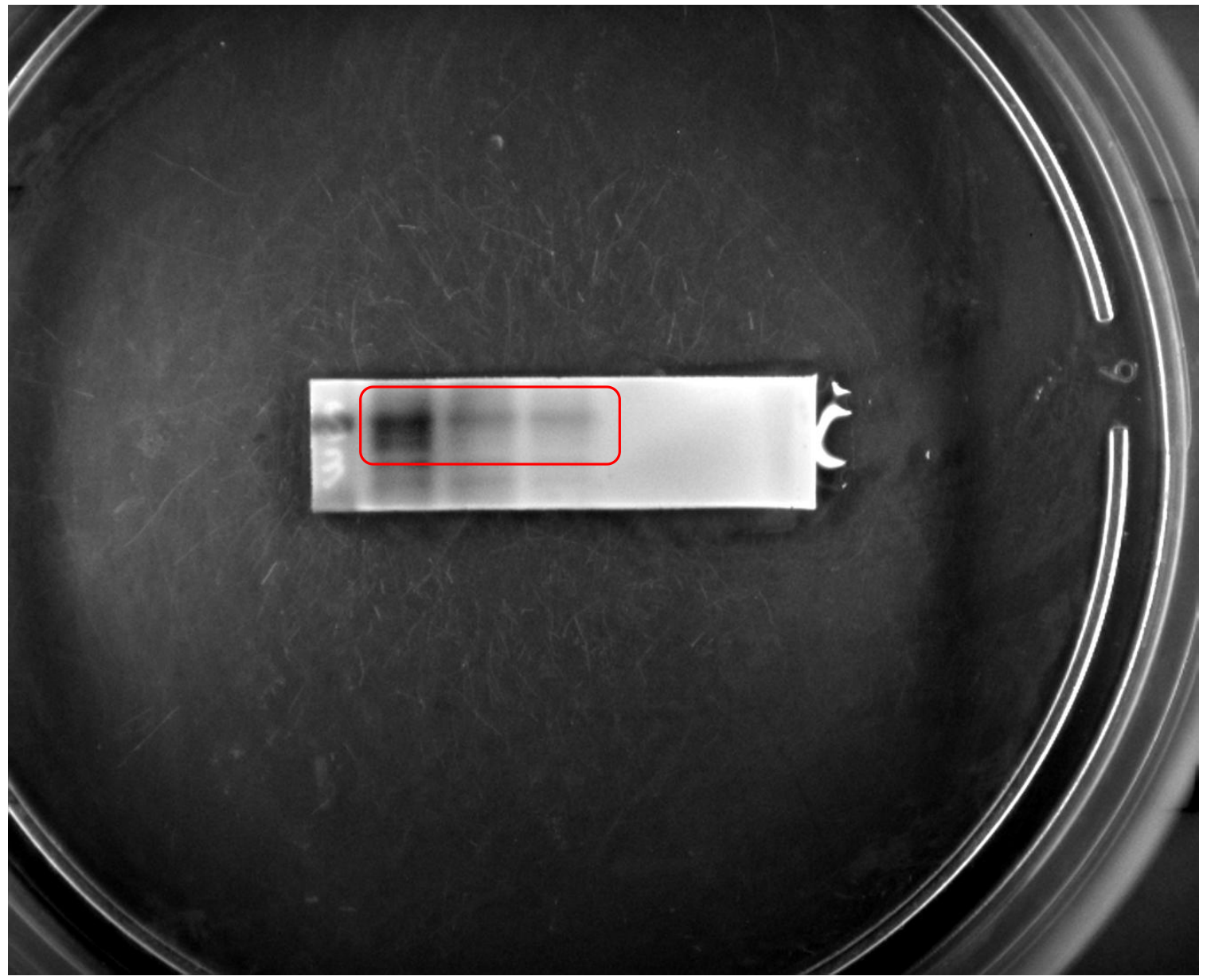

SLC7A11

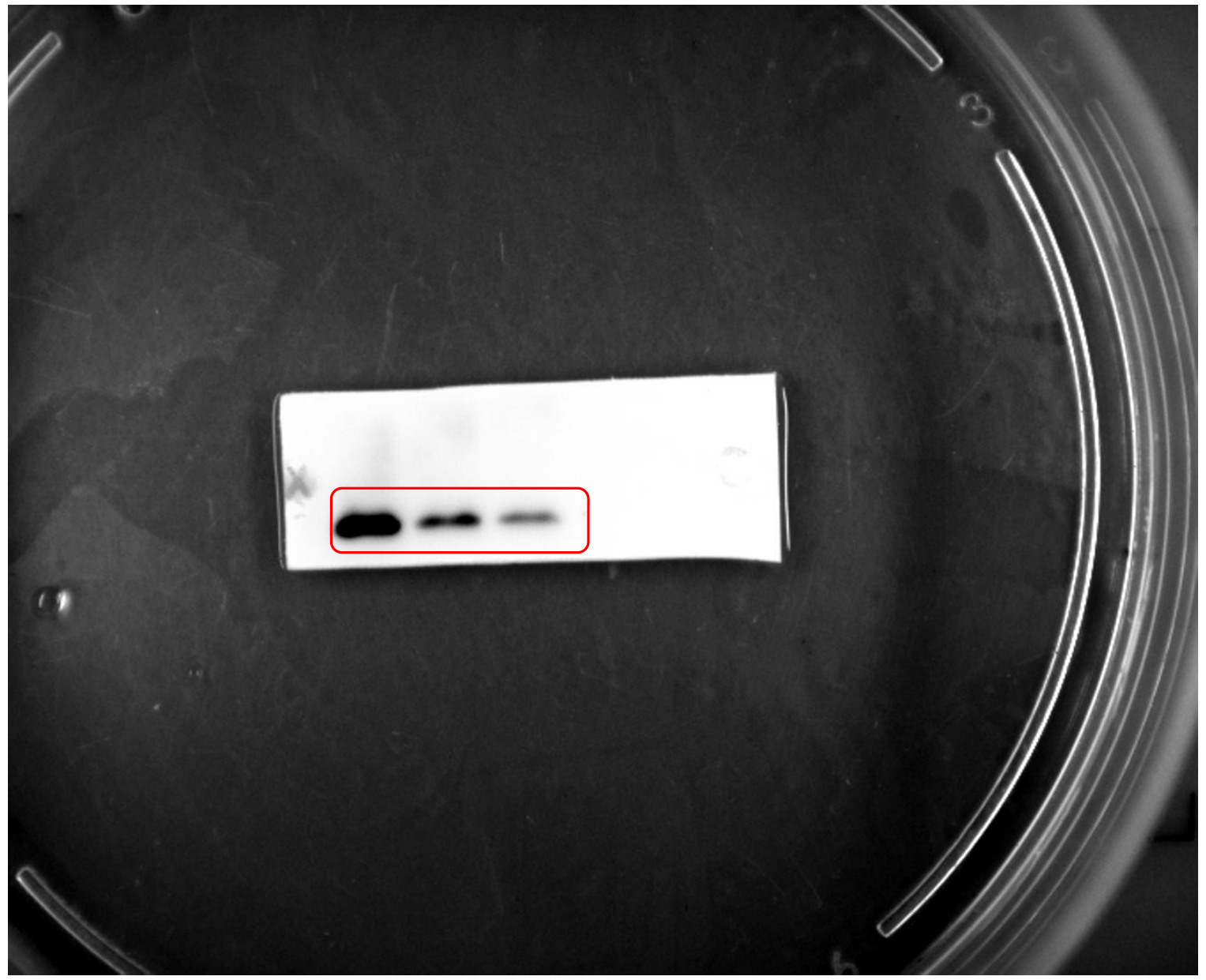

GPX4

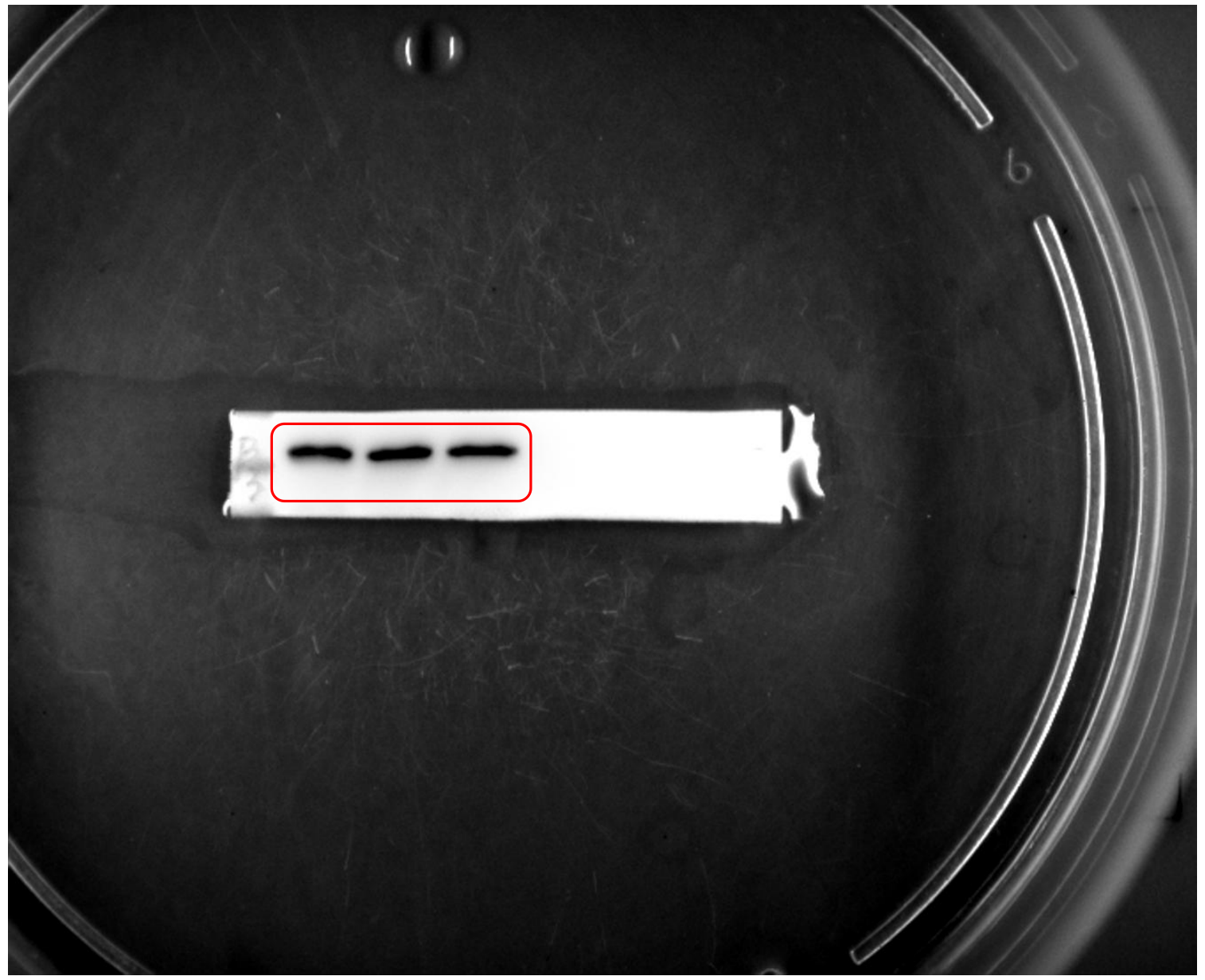

β-actin
